# A Basal Lithostrotian Titanosaur (Dinosauria: Sauropoda) with a Complete Skull: Implications for the Evolution and Paleobiology of Titanosauria

**DOI:** 10.1371/journal.pone.0151661

**Published:** 2016-04-26

**Authors:** Rubén D. F. Martínez, Matthew C. Lamanna, Fernando E. Novas, Ryan C. Ridgely, Gabriel A. Casal, Javier E. Martínez, Javier R. Vita, Lawrence M. Witmer

**Affiliations:** 1 Laboratorio de Paleovertebrados, Universidad Nacional de la Patagonia San Juan Bosco, Comodoro Rivadavia, Chubut, Argentina; 2 Section of Vertebrate Paleontology, Carnegie Museum of Natural History, Pittsburgh, Pennsylvania, United States of America; 3 Laboratorio de Anatomía Comparada y Evolución de los Vertebrados, Museo Argentino de Ciencias Naturales, Buenos Aires, Argentina; 4 Department of Biomedical Sciences, Heritage College of Osteopathic Medicine, Ohio University, Athens, Ohio, United States of America; 5 Hospital Regional de Comodoro Rivadavia, Comodoro Rivadavia, Chubut, Argentina; 6 Resonancia Magnética Borelli, Comodoro Rivadavia, Chubut, Argentina; State Natural History Museum, GERMANY

## Abstract

We describe *Sarmientosaurus musacchioi* gen. et sp. nov., a titanosaurian sauropod dinosaur from the Upper Cretaceous (Cenomanian—Turonian) Lower Member of the Bajo Barreal Formation of southern Chubut Province in central Patagonia, Argentina. The holotypic and only known specimen consists of an articulated, virtually complete skull and part of the cranial and middle cervical series. *Sarmientosaurus* exhibits the following distinctive features that we interpret as autapomorphies: (1) maximum diameter of orbit nearly 40% rostrocaudal length of cranium; (2) complex maxilla—lacrimal articulation, in which the lacrimal clasps the ascending ramus of the maxilla; (3) medial edge of caudal sector of maxillary ascending ramus bordering bony nasal aperture with low but distinct ridge; (4) ‘tongue-like’ ventral process of quadratojugal that overlaps quadrate caudally; (5) separate foramina for all three branches of the trigeminal nerve; (6) absence of median venous canal connecting infundibular region to ventral part of brainstem; (7) subvertical premaxillary, procumbent maxillary, and recumbent dentary teeth; (8) cervical vertebrae with ‘strut-like’ centroprezygapophyseal laminae; (9) extremely elongate and slender ossified tendon positioned ventrolateral to cervical vertebrae and ribs. The cranial endocast of *Sarmientosaurus* preserves some of the most complete information obtained to date regarding the brain and sensory systems of sauropods. Phylogenetic analysis recovers the new taxon as a basal member of Lithostrotia, as the most plesiomorphic titanosaurian to be preserved with a complete skull. *Sarmientosaurus* provides a wealth of new cranial evidence that reaffirms the close relationship of titanosaurs to Brachiosauridae. Moreover, the presence of the relatively derived lithostrotian *Tapuiasaurus* in Aptian deposits indicates that the new Patagonian genus represents a ‘ghost lineage’ with a comparatively plesiomorphic craniodental form, the evolutionary history of which is missing for at least 13 million years of the Cretaceous. The skull anatomy of *Sarmientosaurus* suggests that multiple titanosaurian species with dissimilar cranial structures coexisted in the early Late Cretaceous of southern South America. Furthermore, the new taxon possesses a number of distinctive morphologies—such as the ossified cervical tendon, extremely pneumatized cervical vertebrae, and a habitually downward-facing snout—that have rarely, if ever, been documented in other titanosaurs, thus broadening our understanding of the anatomical diversity of this remarkable sauropod clade. The latter two features were convergently acquired by at least one penecontemporaneous diplodocoid, and may represent mutual specializations for consuming low-growing vegetation.

## Introduction

Titanosaurian sauropod dinosaurs were extremely diverse and abundant in Upper Cretaceous continental paleoenvironments in the Gondwanan landmasses, and have been discovered throughout the world [1–6]. Titanosauria currently includes more than 60 genera and is most abundantly represented in South America, particularly in Argentina [4,5,7,8]. Most currently recognized titanosaurian taxa are represented exclusively or almost exclusively by postcranial bones.

In the years since Huene [9] described the incomplete craniomandibular remains of the Patagonian titanosaur *Antarctosaurus wichmannianus* (hereafter *Antarctosaurus*, since *A*. *wichmannianus* is the only species of this genus mentioned herein), discoveries of well-preserved titanosaurian skulls have been extraordinarily rare. At present, complete or nearly complete skulls are known only for the following taxa: *Nemegtosaurus*, from the Upper Cretaceous Nemegt Formation of Mongolia [10,11]; *Rapetosaurus*, from the Upper Cretaceous Maevarano Formation of Madagascar [12,13]; and *Tapuiasaurus*, from the Lower Cretaceous Quiricó Formation of Brazil [14]. *Tapuiasaurus* was recovered from Aptian strata, whereas *Nemegtosaurus* and *Rapetosaurus* come from Maastrichtian deposits. Moreover, several complete or nearly complete but distorted skulls of generically unidentified embryonic titanosaurs are known from the Campanian Anacleto Formation at the Auca Mahuevo locality in northern Patagonia, Argentina [15,16,17]. Fragmentary skulls or isolated skull elements are also known for a number of titanosaurs or possible titanosaurs, in addition to *Antarctosaurus*: *Ampelosaurus atacis* [18], *Ampelosaurus* sp. [19], *Bonatitan* [20–22], *Bonitasaura* [23,24], *Brasilotitan* [25], *Campylodoniscus* [9], *Dreadnoughtus* [26], *Isisaurus* [27–30], *Jainosaurus* [28,30,31], *Karongasaurus* [32], *Ligabuesaurus* [33], *Lirainosaurus* [34,35], *Malawisaurus* [32], *Maxakalisaurus* [36], *Mongolosaurus* [37,38], *Muyelensaurus* [39], *Narambuenatitan* [40], *Phuwiangosaurus* [41], *Pitekunsaurus* [42], *Quaesitosaurus* [11,43], *Quetecsaurus* [44], *Rinconsaurus* [45], *Saltasaurus* [7,46], *Tambatitanis* [47], and *Vahiny* [48]. Isolated, generically indeterminate titanosaurian cranial and mandibular elements have also been reported [7,9,21,31, 49–64]. Nevertheless, complete or even reasonably complete titanosaur skulls remain unknown from the Albian—Santonian (a roughly 30 million year span of the mid- and Late Cretaceous; see Walker et al. [65]), which represents a significant impediment to understanding of titanosaur cranial anatomy and evolution.

Here we describe a new and plesiomorphic early Late Cretaceous (Cenomanian—Turonian) titanosaurian sauropod represented by a superbly-preserved adult skull articulated with a partial cervical series. The taxon provides a wealth of new information on the early evolution of Titanosauria and the cranial anatomy of basal members of the clade. The cranium and mandible are only slightly deformed, with most bones fully articulated and all teeth preserved *in situ*; as such, the new form is one of the very few titanosaurs for which the totality of this anatomical information is available. Furthermore, the unusual anatomy of the cervical series provides novel data on the construction of the neck and tendon system of a Cretaceous sauropod.

The new titanosaur comes from an exposure of the Lower Member of the Upper Cretaceous Bajo Barreal Formation on the Estancia Laguna Palacios near the village of Buen Pasto in south-central Chubut Province, central Patagonia, Argentina. The Bajo Barreal Formation has produced a diverse continental vertebrate fauna that is among the richest of Patagonian dinosaur-bearing units [66–75]. Its outcrops are widely distributed in southern Chubut and northernmost Santa Cruz provinces. Palynological data from a subsurface equivalent of the Bajo Barreal Formation, the Caleta Olivia Member of the Cañadón Seco Formation, initially suggested a late Albian—Cenomanian age for these deposits [76]. Subsequently, Bridge et al. [77] reported Ar—Ar radiometric dates from tuffs of the Bajo Barreal Formation that range in age from 95.8 to 91.0 Ma, corresponding to the middle Cenomanian—middle Turonian of the current Geologic Time Scale [65]. Most recently, Suárez et al. [78] obtained radiometric ages from zircons that further support a Cenomanian age for the Lower Member. Clyde et al. [79] argued for a much younger (Campanian) age for the Bajo Barreal Formation on the basis of magnetostratigraphic and biostratigraphic evidence, but the strata they investigated—exposed in the regions of Lago Colhué Huapi and the Río Chico in southeastern Chubut—have recently been reassigned to a newly identified and significantly younger geologic unit (the Lago Colhué Huapi Formation [80]). As noted by Canale et al. [81], the vertebrate fauna of the Bajo Barreal Formation is closely comparable to that of the lower Cenomanian [82] Candeleros Formation of the Neuquén Basin in northern Patagonia, suggesting that the two units may be correlative.

The titanosaur was preserved in a green sandstone horizon that pertains to the upper part of the Lower Member of the Bajo Barreal Formation. This section of the Lower Member is lithologically characterized by these green sandstones, which were deposited in multiepisodic, interlaced fluvial channel systems [83]. The vast majority of the tetrapod fossils from the Bajo Barreal Formation have been recovered from these sandstones, which exhibit taphonomic and sedimentological properties that were conducive to vertebrate preservation [84]. The skull and cervical series of the titanosaur were articulated and preserved in a fluvial overflow deposit with a high sedimentary load composed of medium-grained sandstones with abundant pelitic matrix. The degree of articulation and lack of evidence of subaerial weathering of the specimen suggest that it was buried rapidly.

## Materials and Methods

### Paleontological Ethics Statements

The specimen described in this paper (specimen number MDT-PV 2) is permanently reposited and accessible to all qualified researchers in the fossil vertebrate collection of the Museo Desiderio Torres in Sarmiento, Chubut Province, Argentina. Detailed locality information for the specimen is on file at the Museo Desiderio Torres and is available to qualified researchers upon request. All necessary permits were obtained for the described study, which complied with all relevant regulations.

### Institutional Abbreviations

AMNH, American Museum of Natural History, New York, New York, United States of America; ANS, Academy of Natural Sciences of Drexel University, Philadelphia, Pennsylvania, United States of America; CCMGE, Chernyshev’s Central Museum of Geological Exploration, Saint Petersburg, Russia; CM, Carnegie Museum of Natural History, Pittsburgh, Pennsylvania, United States of America; FGGUB, Facultatea de Geologie şi Geofizică a Universită ii din Bucureşti, Bucharest, Romania; GCP, Grupo Cultural Paleontológico de Elche, Museo Paleontológico de Elche, Elche, Spain; GSI, Geological Survey of India, Kolkata, India; ISI, Indian Statistical Institute, Kolkata, India; MAL, Malawi Department of Antiquities Collection, Lilongwe and Nguludi, Malawi; MB, Museum für Naturkunde der Humboldt-Universität, Berlin, Germany; MCF-PVPH, Museo ‘Carmen Funes,’ Colección de Paleontología de Vertebrados, Plaza Huincul, Neuquén, Argentina; MCSPv, Museo de Cinco Saltos, Cinco Saltos, Río Negro, Argentina; MCZ, Museum of Comparative Zoology, Harvard University, Cambridge, Massachusetts, United States of America; MDT-PV, Museo Desiderio Torres-Paleovertebrados, Sarmiento, Chubut, Argentina; MGPIFD-GR, Museo de Geología y Paleontología del Instituto de Formación Docente Continua de General Roca, General Roca, Río Negro, Argentina; MML, Museo Municipal de Lamarque, Lamarque, Río Negro, Argentina; MPCA, Museo Provincial ‘Carlos Ameghino,’ Cipolletti, Río Negro, Argentina; MUCPv, Museo de Geología y Paleontología de la Universidad Nacional del Comahue, Neuquén, Neuquén, Argentina; MZSP-PV, Museu de Zoologia da Universidade de São Paulo, São Paulo, Brazil; TMM, University of Texas Memorial Museum, Austin, Texas, United States of America; UNPSJB-PV, Universidad Nacional de la Patagonia San Juan Bosco, Colección Paleontología de Vertebrados, Comodoro Rivadavia, Chubut, Argentina; USNM, National Museum of Natural History, Washington, District of Columbia, United States of America; ZPAL, Institute of Paleobiology, Polish Academy of Sciences, Warsaw, Poland.

### Anatomical Abbreviations

a, angular; af, adductor fossa; al, alveolus; aof, antorbital fenestra; arm, ascending ramus of maxilla; awf, apical wear facet; ax, axis; b, bulge; bna, bony nasal aperture; bo, basioccipital; bpt, basipterygoid process; bs, basisphenoid; bt, basal tuber; bwf, beveled wear facet; C, cervical vertebra; c, cochlea; car, canal for cerebral carotid artery; cc, crus communis; cd, condyle; cde, caudal dural expansion; cer, cerebral hemisphere; cor, coronoid; cprs, centroprezygapophyseal ‘strut’; csc, caudal (vertical) semicircular canal; csf, caudal surangular foramen; cts, cerebrotectal (sphenoparietal) venous sinus; d, dentary; df, dental foramen; dwf, distal wear facet; ec, ectopterygoid; ed, endolymphatic duct; f, frontal; fc, fenestra cochleae (fenestra rotunda); fi, fibroblasts; floc, cerebellar flocculus (auricle); fom, foramen magnum; fv, fenestra vestibuli (fenestra ovalis); itf, infratemporal fenestra; j, jugal; l, lacrimal; lab, labial surface; labyr, endosseous labyrinth; lgr, large groove; lin, lingual surface; ls, laterosphenoid; lsc, lateral (horizontal) semicircular canal; lwf, lingual wear facet; m, maxilla; mg, Meckelian groove; mgr, mesial groove; n, nasal; nf, narial fossa; nvf, neurovascular foramen; ns, neural spine; ob, olfactory bulb; occ sin, occipital (dural venous) sinus; ocv, canal for orbitocerebral vein; orb, orbit; os, orbitosphenoid; oto, otoccipital; p, parietal; paof, preantorbital foramen; pf, pneumatic fossa; pfo, pituitary (hypophyseal) fossa; pl, palatine; pm, premaxilla; po, postorbital; podl, postzygodiapophyseal lamina; pof, postorbital foramen; poz, postzygapophysis; pra, prearticular + articular; prdl, prezygodiapophyseal lamina; prf, prefrontal; prz, prezygapophysis; pt, pterygoid; q, quadrate; qf, quadrate fossa; qj, quadratojugal; r, rib; rde, rostral dural expansion; rmf, rostral maxillary foramen; rsc, rostral (vertical) semicircular canal; rsca, ampulla of rostral (vertical) semicircular canal; rsf, rostral surangular foramen; sa, surangular; snf, subnarial foramen; so, supraoccipital; sof, suborbital fenestra; sp, splenial; spha, canal for sphenopalatine artery; sprl, spinoprezygapophyseal lamina; sq, squamosal; stf, supratemporal fenestra; sym, mandibular symphysis; t, tooth; ts, transverse (dural venous) sinus; ttv, canal for transversotrigeminal (rostral middle cerebral) vein; tz, transitional zone; v, vomers; ve, vestibule of inner ear; II, canal for optic nerve; III, canal for oculomotor nerve; IV, canal for trochlear nerve; V_1_, canal for ophthalmic branch of trigeminal nerve; V_1-SO?_, canal possibly for the supraorbital nerve (a branch of CN V_1_); V_2_, canal for maxillary branch of trigeminal nerve; V_3_, canal for mandibular branch of trigeminal nerve; VI, canal for abducens nerve; VII, canal for facial nerve; VIII, canal for vestibulocochlear nerve; IX–XI, shared canal for glossopharyngeal, vagus, and accessory nerves and accompanying vessels; XII, canal for hypoglossal nerve.

### Nomenclatural Acts

The electronic edition of this article conforms to the requirements of the amended International Code of Zoological Nomenclature (ICZN), and hence the new names contained herein are available under that Code from the electronic edition of this article. This published work and the nomenclatural acts it contains have been registered in ZooBank, the online registration system for the ICZN. The ZooBank Life Science Identifiers (LSIDs) can be resolved and the associated information viewed through any standard web browser by appending the LSID to the prefix “http://zoobank.org/.” The LSID for this publication is: urn:lsid:zoobank.org:pub:3B8C51B9-C0C2-4562-81D4-0AF58E186B31. The electronic edition of this work was published in a journal with an ISSN, and has been archived and is available from the following digital repositories: PubMed Central and LOCKSS.

## Results

### Systematic Paleontology

Saurischia Seeley 1887 [85]

Sauropodomorpha Huene 1932 [86]

Sauropoda Marsh 1878 [87]

Titanosauriformes Salgado, Coria, and Calvo 1997 [88]

Titanosauria Bonaparte and Coria 1993 [89]

Lithostrotia Upchurch, Barrett, and Dodson 2004 [90]

*Sarmientosaurus* gen. nov.

urn:lsid:zoobank.org:act:537DFE26-54EC-4978-AC86-E83A04FA74DE

*Sarmientosaurus musacchioi* sp. nov.

urn:lsid:zoobank.org:act:C1090B8D-D051-44F3-B869-8B4A0C802176

#### Holotype

MDT-PV 2, an originally articulated cranial and cervical skeleton consisting of the nearly complete skull, the partial axis associated with its rib from the right side and articulated with the cranial part of the third cervical vertebra, a fragment of the fifth cervical vertebra, the nearly complete sixth cervical vertebra and its right rib, the partial seventh cervical vertebra, and a section of ossified cervical tendon.

#### Etymology

Sarmiento, for the Patagonian town and the administrative department in which it is located, the latter of which has yielded numerous Cretaceous dinosaur fossils; saurus, Greek, ‘lizard.’ The specific name honors the late Dr. Eduardo Musacchio, a model scientist and educator at the Universidad Nacional de la Patagonia San Juan Bosco in Comodoro Rivadavia, Argentina.

#### Locality and horizon

**Fig 1:**
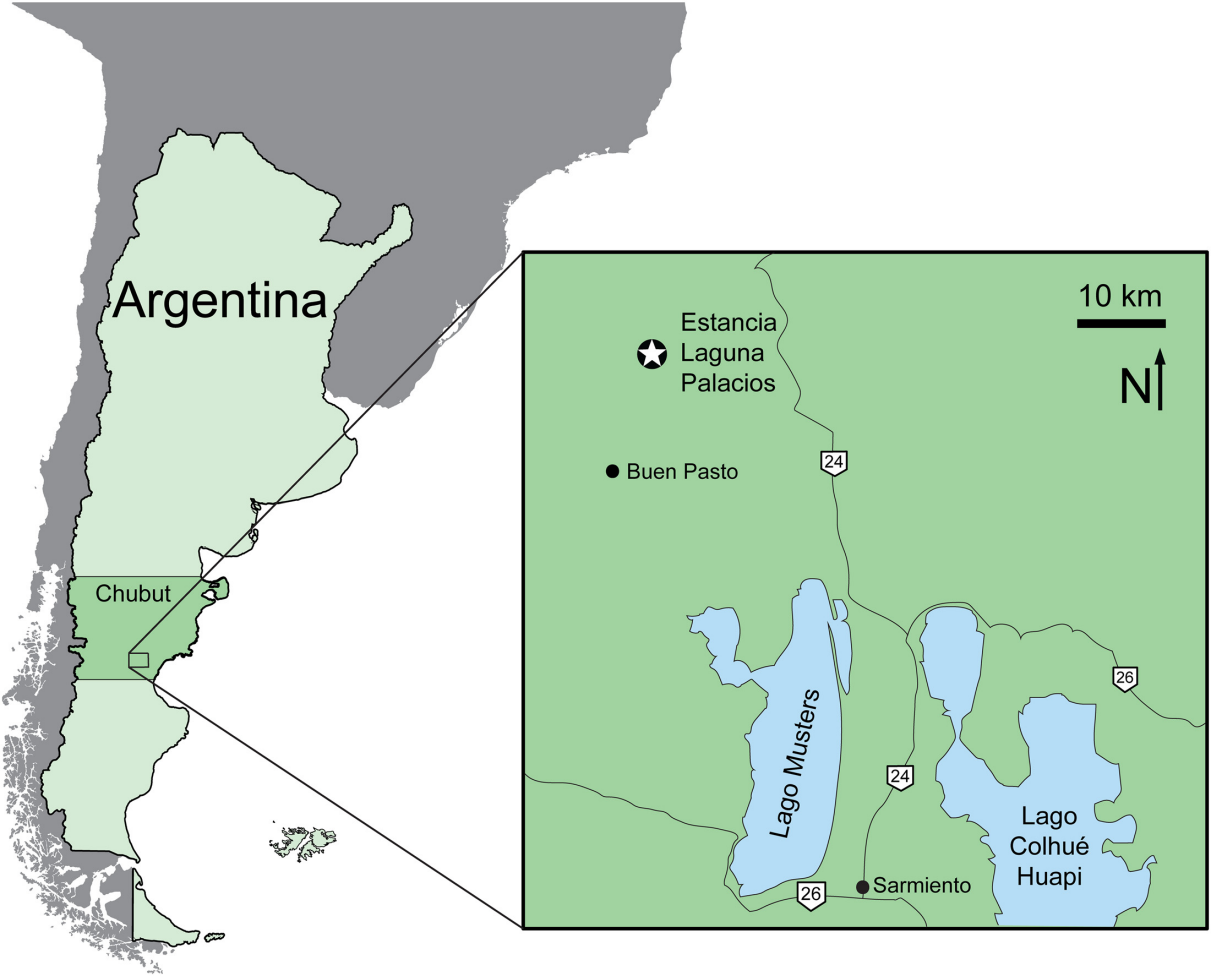
Map of Chubut Province, central Patagonia, Argentina, showing location of the Estancia Laguna Palacios, the type locality of *Sarmientosaurus musacchioi* gen. et sp. nov. (modified from Ibiricu et al. [232]).

Estancia Laguna Palacios (44°54'11.6'' S, 69°22'56.7'' W), Sierra Nevada Anticline, Golfo San Jorge Basin, south-central Chubut Province, central Patagonia, Argentina (Fig 1). Uppermost section of the Lower Member of the Upper Cretaceous Bajo Barreal Formation, Chubut Group. The specimen was found *in situ* in a tuffaceous sandstone that is regarded as Cenomanian—Turonian in age [69,72,76–78,80].

#### Diagnosis

Basal lithostrotian titanosaurian sauropod diagnosed by the following autapomorphies: (1) maximum (rostroventral—caudodorsal) diameter of orbit nearly 40% rostrocaudal length of cranium (as measured from tip of snout to occipital condyle); (2) complex maxilla—lacrimal articulation, with ascending ramus of maxilla embedded in and bordered laterally and medially by lacrimal dorsal process; (3) medial edge of caudal sector of maxillary ascending ramus bordering bony nasal aperture with low but well-defined ridge; (4) ‘tongue-like’ ventral process of quadratojugal that overlaps quadrate caudally; (5) separate foramina for all three branches of the trigeminal nerve; (6) absence of median venous canal connecting infundibular region to ventral part of brainstem; (7) premaxillary teeth subvertical, maxillary teeth procumbent, and dentary teeth recumbent; (8) middle cervical vertebrae with ‘strut-like’ (as opposed to ‘sheet-like’) centroprezygapophyseal laminae; (9) extremely elongate and slender ossified tendon extending along cervical series ventrolateral to vertebrae and ribs.

#### Preservation

**Fig 2:**
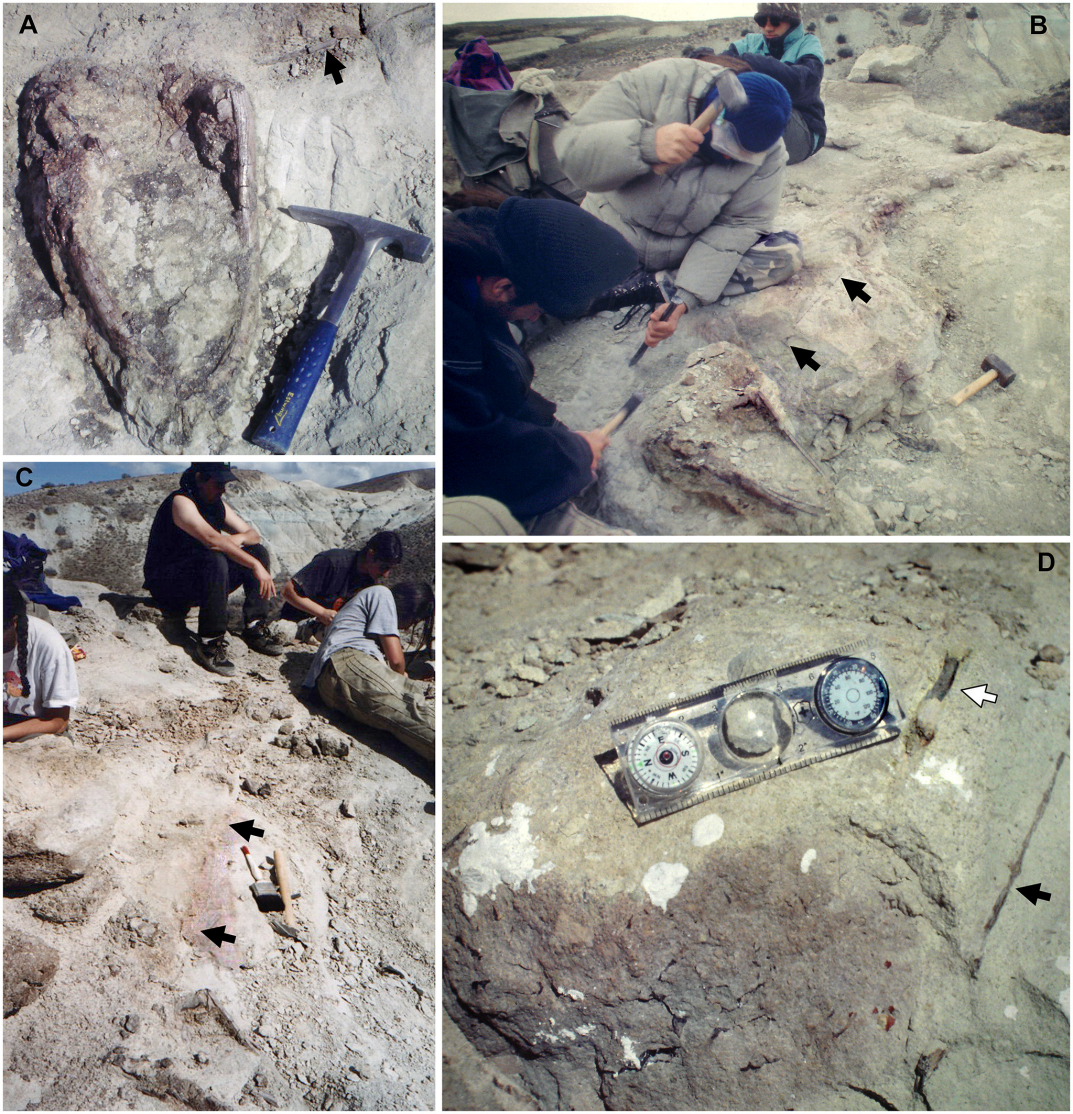
Disposition of the type specimen of *Sarmientosaurus musacchioi* gen. et sp. nov. (MDT-PV 2) upon discovery. (**A**) Articulated skull in ventral view, showing close association of ossified cervical tendon (arrow) with occipital region of cranium. (**B**, **C**) Two views of articulated skull and partial cervical series in ventral view, showing considerable craniocaudal extent and consistently narrow diameter of ossified cervical tendon (arrows). (**D**) Relationship of a cervical rib (white arrow) with the ossified cervical tendon (black arrow).

The cranium, mandible, and all preserved cervical vertebrae and ribs of the new titanosaur were originally found in articulation (Fig 2). Nevertheless, during the course of laboratory preparation, we were only able to recover the skull, parts of the articulated axis and third cervical vertebra, most of the sixth and seventh cervical vertebrae, and pieces of the fifth cervical vertebra and the second and sixth cervical ribs from the right side. Unfortunately, the remainder of the collected vertebrae (the atlas and cervical four) and ribs were too poorly preserved and damaged by weathering to be salvageable.

In the field, we observed a very slender, dark, cylindrical structure situated adjacent to and oriented parallel to the right ventrolateral area of the articulated cervical vertebrae and ribs (Fig 2). This structure extended from near the right occipital region of the skull and past several vertebrae without changing diameter. Although we observed the structure on only the right side of the specimen, we assume that it was bilaterally symmetrical in the living animal. Therefore, given that the right side of the specimen is generally better preserved than the left, the equivalent structure on the left side presumably eroded away prior to discovery.

Given the extraordinary length attained by the cervical ribs of some sauropods (e.g., mamenchisaurids [91,92]), including other titanosauriforms (e.g., *Giraffatitan* [93], *Sauroposeidon* [94]), it is conceivable that this structure might represent the caudal end of one of these ribs. Nevertheless, as observed in the field, the structure maintained its same, diminutive diameter alongside several cervical vertebrae, and its cranial extreme was situated immediately caudal to the skull, morphologies that are inconsistent with known sauropod cervical ribs. Furthermore, because the skull, cervical vertebrae, and ribs were all fully articulated, the identification of this structure as a displaced cervical rib shaft seems unlikely. We therefore interpret the structure as an ossified tendon that is distinct from the cervical ribs.

Unlike the situation in *Nemegtosaurus* [10,11] and *Tapuiasaurus* [14], the skull of *Sarmientosaurus* was not strongly affected by taphonomic distortion. Instead, the skull is only moderately deformed in its caudodorsal and dorsal areas. Pressure applied to these regions apparently caused the jugal processes of both postorbitals to slide slightly rostrally over the postorbital processes of the corresponding jugals. Nevertheless, these modest alterations demonstrate that the caudal part of the skull was not significantly rostrally displaced relative to more rostral regions. There is no evidence of dorsoventral compression of the snout; indeed, in this area of the skull, only the dorsal parts of the premaxilla and maxilla are damaged, presumably due to pre-diagenetic erosion. The sixth and seventh cervical vertebrae have suffered some lateral deformation, which has mainly affected parts of the neural arches such as the prezygapophyses.

During the excavation of the *Sarmientosaurus* holotype, an abelisaurid tooth was discovered only a few centimeters from the occipital region of the skull, raising the possibility that this titanosaurian specimen was scavenged by this theropod. This is, however, ambiguous, as the *Sarmientosaurus* bones do not exhibit tooth marks or other feeding traces.

### Description and Comparisons

#### Anatomical Terminology

In our description of the dentition of *Sarmientosaurus*, we employ the terms used by García and Cerda [61].

### Cranium

The cranium of *Sarmientosaurus* is 43 cm in length as measured from the rostral tip of the articulated premaxillae to the occipital condyle. It is approximately 24 cm wide across the postorbitals and 24 cm tall from the dorsal margin of the frontal to the ventral end of the quadrate on the right side (see Table 1). Extreme fusion of many cranial bones, as in specimens of *Ampelosaurus* [18,19] and *Saltasaurus* [7,46], indicates that the specimen probably corresponds to a skeletally mature (and possibly very old) individual.

**Table 1:** Measurements (mm) of the skull of MDT-PV 2, the holotype of *Sarmientosaurus musacchioi* gen. et sp. nov. Abbreviations: L, left; R, right. * = element incomplete, measurement as preserved.

Dimension, element	Measurement
Rostrocaudal length, cranium	430
Dorsoventral height, cranium (from quadrate)	240
Transverse width, cranium (maximum, in occipital region)	245
Rostrocaudal length, orbit	88L, 94R
Dorsoventral height, orbit	150L, 157R
Transverse width, external narial opening (maximum)	118
Rostrocaudal length, infratemporal fenestra	107R
Mediolateral width, supratemporal fenestra	70L, 78R
Rostrocaudal length, supratemporal fenestra	25L, 31R
Rostrocaudal length, alveolar margin of premaxilla	70L, 80R
Rostrocaudal length, alveolar margin of maxilla	170L, 180R
Length, squamosal	95R
Length, lacrimal (maximum)	76L, 93R
Rostrocaudal length, orbital sector of frontal	38L, 35R
Mediolateral width, parietal	108L, 117R
Length, parietal crest	65L, 77R
Dorsoventral height, supraoccipital	34
Transverse width, supraoccipital	31
Dorsoventral height, foramen magnum	33
Transverse width, foramen magnum	24
Rostrocaudal length, mandible (in dorsal view)	283L*, 355R
Rostrocaudal length, mandible (along curve)	300L*, 390R
Dorsoventral height, mandible (maximum, @ adductor fossa)	62L*, 81R
Dorsoventral height, mandible (minimum, @ 11^th^ alveolus)	52L, 48R
Rostrocaudal length, alveolar margin of dentary	190L, 191R
Dorsoventral height, symphysis	46L, 43R

### External Cranial Fenestrae

In the cranium of *Sarmientosaurus*, three large openings are clearly visible in lateral view: from rostral to caudal, these are the antorbital fenestra, the orbit, and the infratemporal fenestra. As preserved, the bony nasal apertures (= ‘external nares’ of many paleontological works) open rostrodorsally in a confluent fenestra, as in *Rapetosaurus*; nevertheless, it appears that, in life, these openings would have been separated by a bony lamina formed by the premaxillae and nasals (the internarial bar). Although this structure has been mostly destroyed by taphonomic processes, the caudally-incomplete narial flange of the premaxillae and a broken rostral projection of the nasals attest to its former existence. Ventral to the rostral end of each antorbital fenestra is a minute, poorly preserved opening that we interpret as the homolog of the preantorbital fenestra; this foramen is discussed further in of our description of the maxilla below.

The antorbital fenestra of the new Patagonian taxon is small, and its long axis is aligned obliquely with respect to that of the skull. It is teardrop-shaped, with the wider, rounded terminus situated rostroventrally and the pointed end positioned caudodorsally. The antorbital fenestra of *Sarmientosaurus* resembles that of the Jurassic brachiosaurid *Giraffatitan* [95] but differs from those of the basal macronarians *Camarasaurus* [96] and *Europasaurus* [97] and the Cretaceous titanosauriforms *Abydosaurus* [98] and *Euhelopus* [99–101], which are oriented more vertically. The fenestra of *Sarmientosaurus* also differs from those of *Nemegtosaurus* [10,11] and *Tapuiasaurus* [14], which are larger, and especially that of *Rapetosaurus*, which is extremely large and rostrocaudally elongate [13]. A greatly enlarged antorbital fenestra also appears to be present in an isolated sauropod (presumably titanosaurian [102]) maxilla from the Maastrichtian Lameta Formation of India (ISI R K 27/528; see Huene and Matley [31]:fig. 19). The shape of the rostral edge of the lacrimal of the Late Cretaceous titanosaur *Bonitasaura* indicates that the caudodorsal margin of the antorbital fenestra was smoothly rounded in this taxon [24] rather than sharply acute as in *Sarmientosaurus*. The antorbital fenestra of the new Patagonian taxon is oriented at an angle of approximately 45° relative to the rostrocaudal axis of the skull, comparable to the condition in *Giraffatitan* and that reconstructed for *Bonitasaura*.

The orbit of *Sarmientosaurus* is proportionally very large, rostroventrally—caudodorsally elongate, and rounded at its caudodorsal and rostroventral margins, with the caudodorsal end rostrocaudally longer than the rostroventral end. As in many dinosaurs, the orbit is regionally divisible into a dorsal ocular portion (that housed the eyeball and its adnexa) and a ventral non-ocular portion that was occupied by various soft-tissues (e.g., adductor muscles, vessels, nerves). The orbit differs from those of *Camarasaurus*, *Giraffatitan*, *Nemegtosaurus*, *Rapetosaurus*, and *Tapuiasaurus*, which are smaller and shaped differently. Although the orbit of *Abydosaurus* is also proportionally large, it is not as large as in the new Bajo Barreal titanosaur; furthermore, it is subtriangular rather than ovate in contour.

The supratemporal fenestra is bordered caudally by a prominent flange (the transverse nuchal crest), and its long axis is oriented mediolaterally, as in *Europasaurus*, *Giraffatitan*, and *Rapetosaurus*. The infratemporal fenestra is rostrocaudally narrow throughout its dorsoventral extent, and its long axis is oriented roughly parallel to that of the orbit, as in *Nemegtosaurus* and *Tapuiasaurus*. This contrasts the conditions in *Abydosaurus*, *Camarasaurus*, *Euhelopus*, and *Giraffatitan*, in which this fenestra is subtriangular and rostrocaudally wide, especially ventrally.

### Dermal Cranial Bones

#### Premaxilla

**Fig 3:**
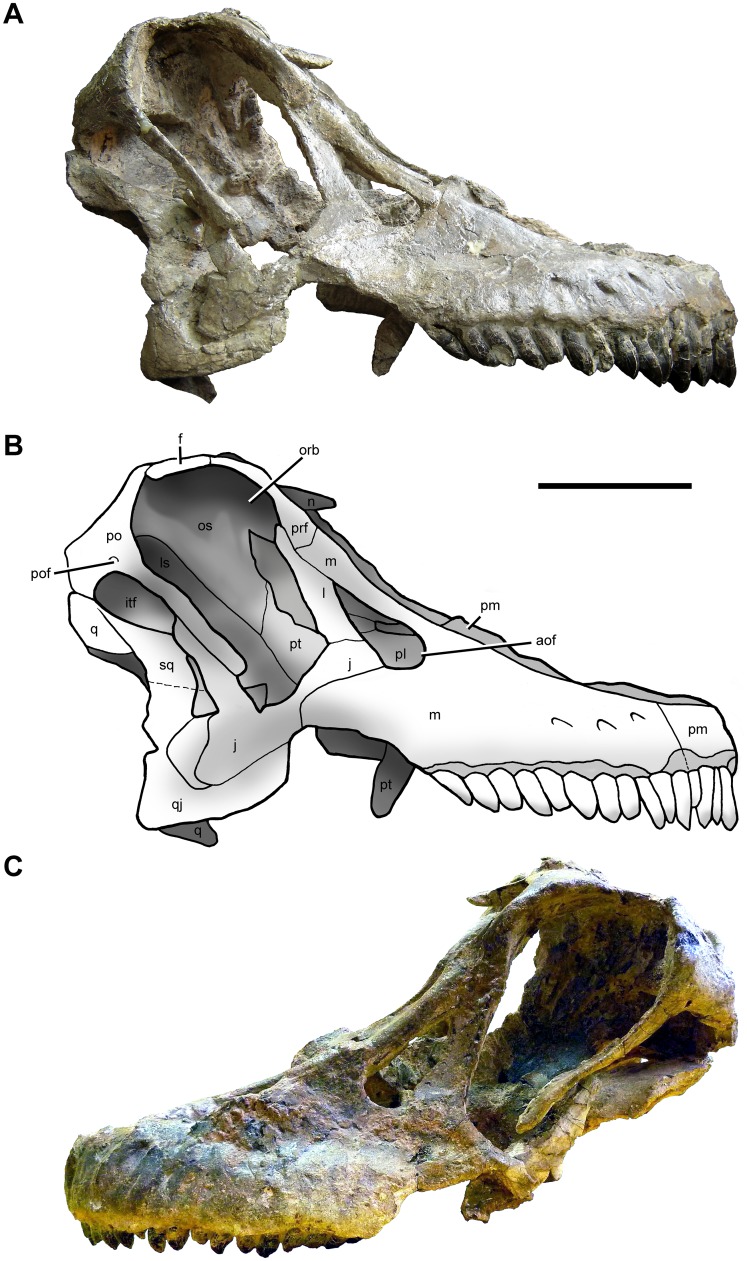
Cranium of *Sarmientosaurus musacchioi* gen. et sp. nov. (MDT-PV 2). Photographs (**A**, **C**) and interpretive drawing (**B**) in right lateral (**A**, **B**) and left lateral (**C**) views. Abbreviations see text. Scale bar = 10 cm.

**Fig 4:**
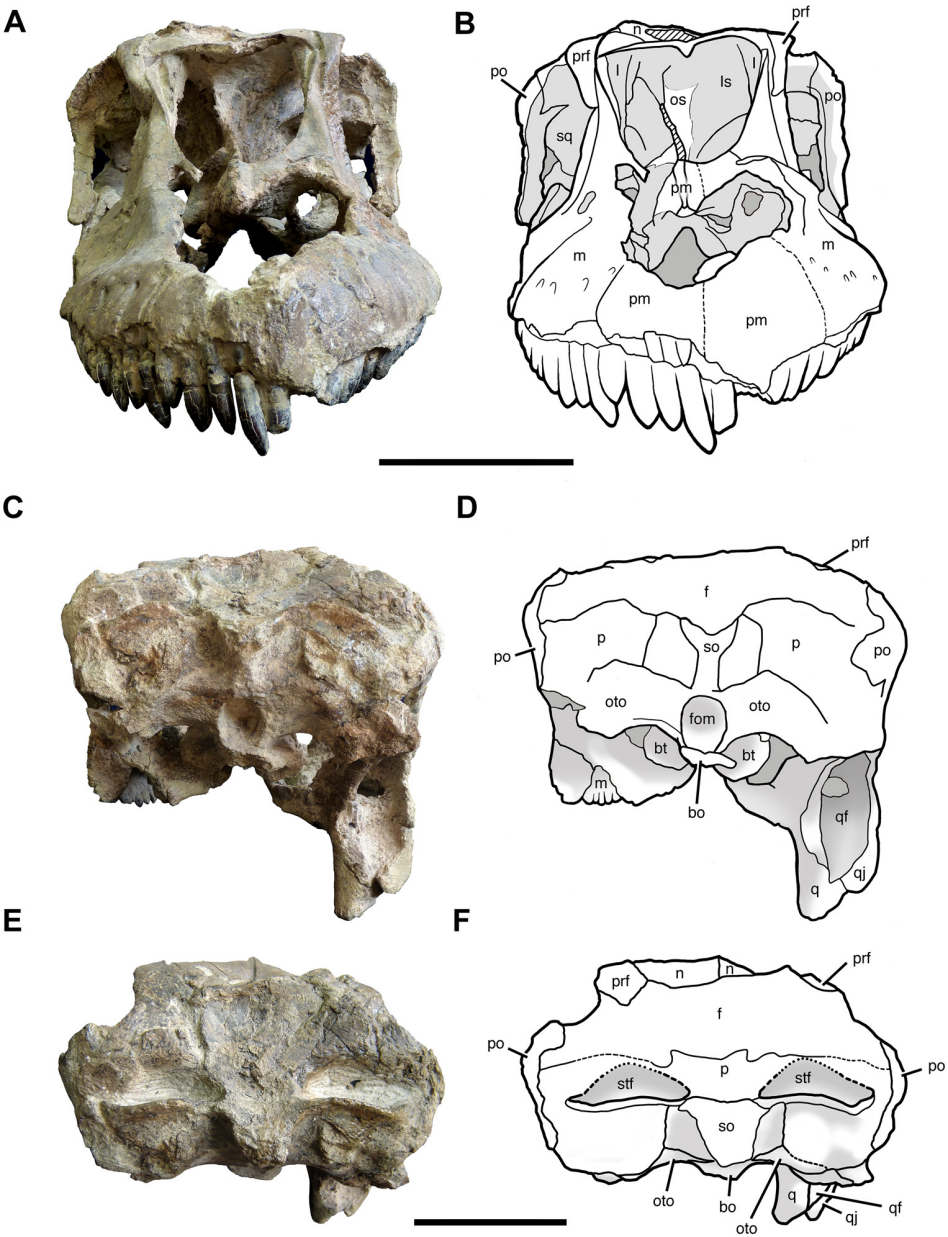
Cranium of *Sarmientosaurus musacchioi* gen. et sp. nov. (MDT-PV 2). Photographs (**A**, **C**, **E**) and interpretive drawings (**B**, **D**, **F**) in rostral (**A**, **B**), caudal (**C**, **D**), and caudodorsal (**E**, **F**) views. Abbreviations see text. Scale bars = 10 cm.

**Fig 5:**
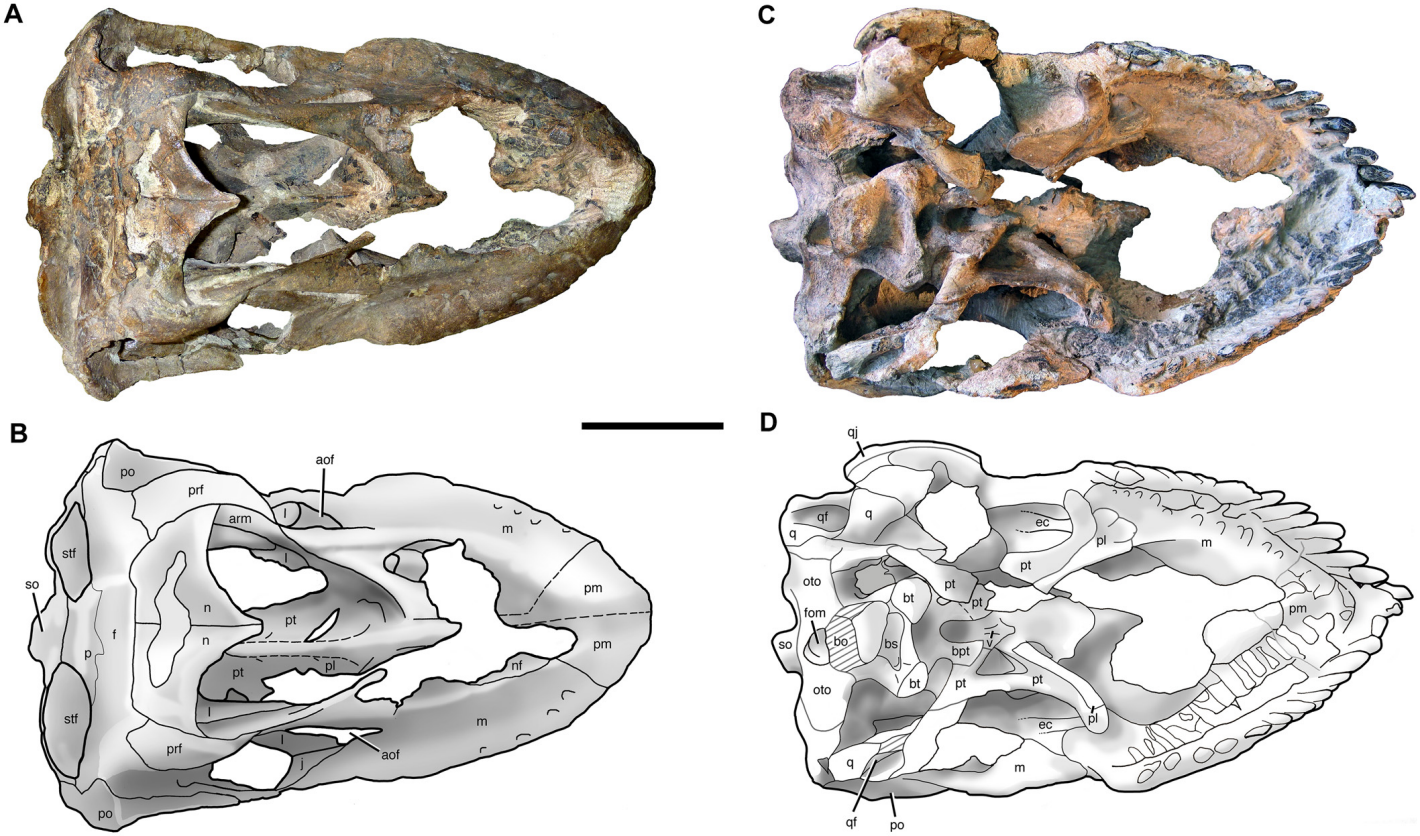
Cranium of *Sarmientosaurus musacchioi* gen. et sp. nov. (MDT-PV 2). Photographs (**A**, **C**) and interpretive drawings (**B**, **D**) in dorsal (**A**, **B**) and ventral (**C**, **D**) views. Abbreviations see text. Scale bars = 10 cm.

**Fig 6:**
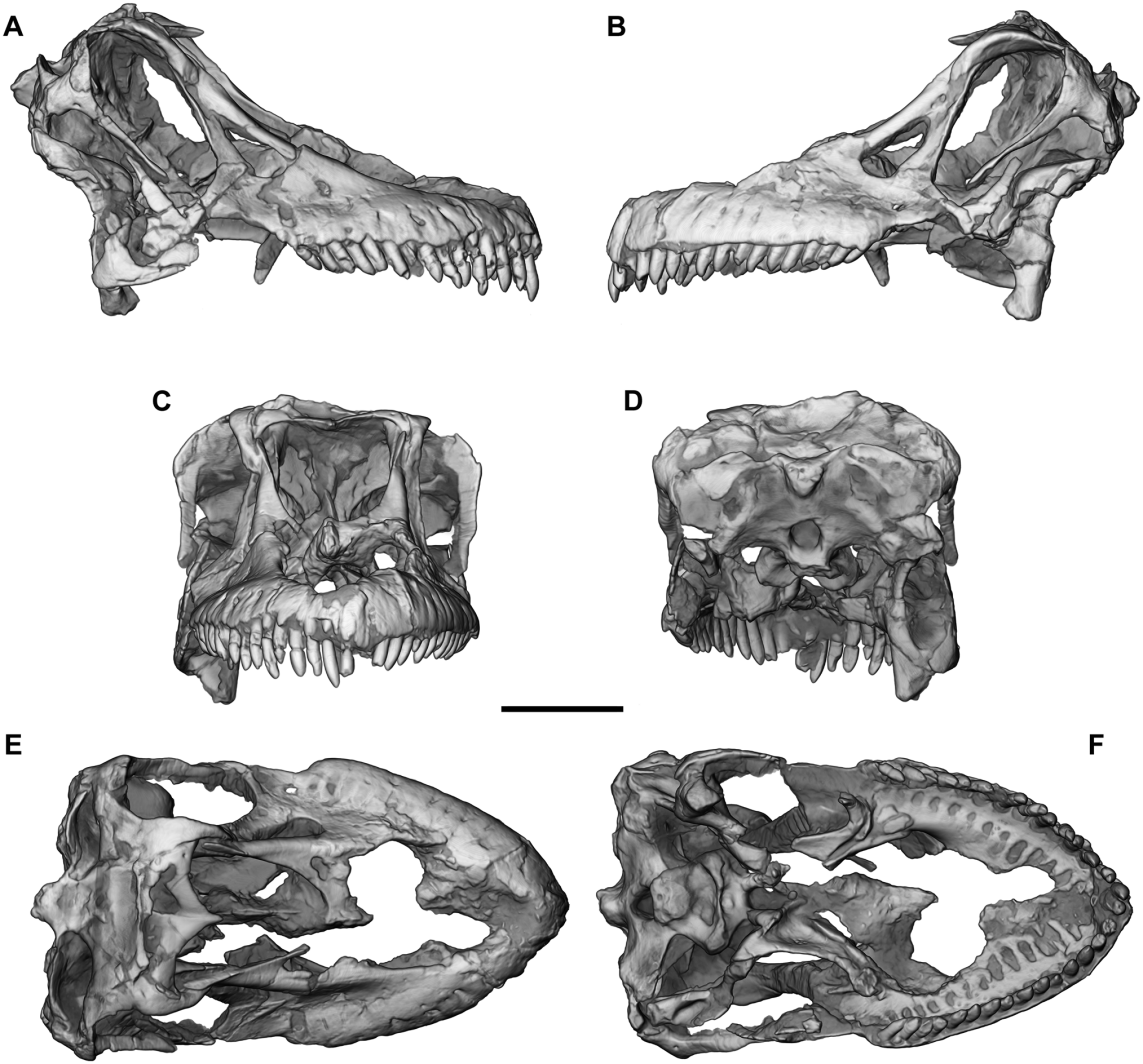
Cranium of *Sarmientosaurus musacchioi* gen. et sp. nov. (MDT-PV 2). Computed tomography-based digital visualization in right lateral (**A**), left lateral (**B**), rostral (**C**), caudal (**D**), dorsal (**E**), and ventral (**F**) views. Scale bar = 10 cm.

**Fig 7:**
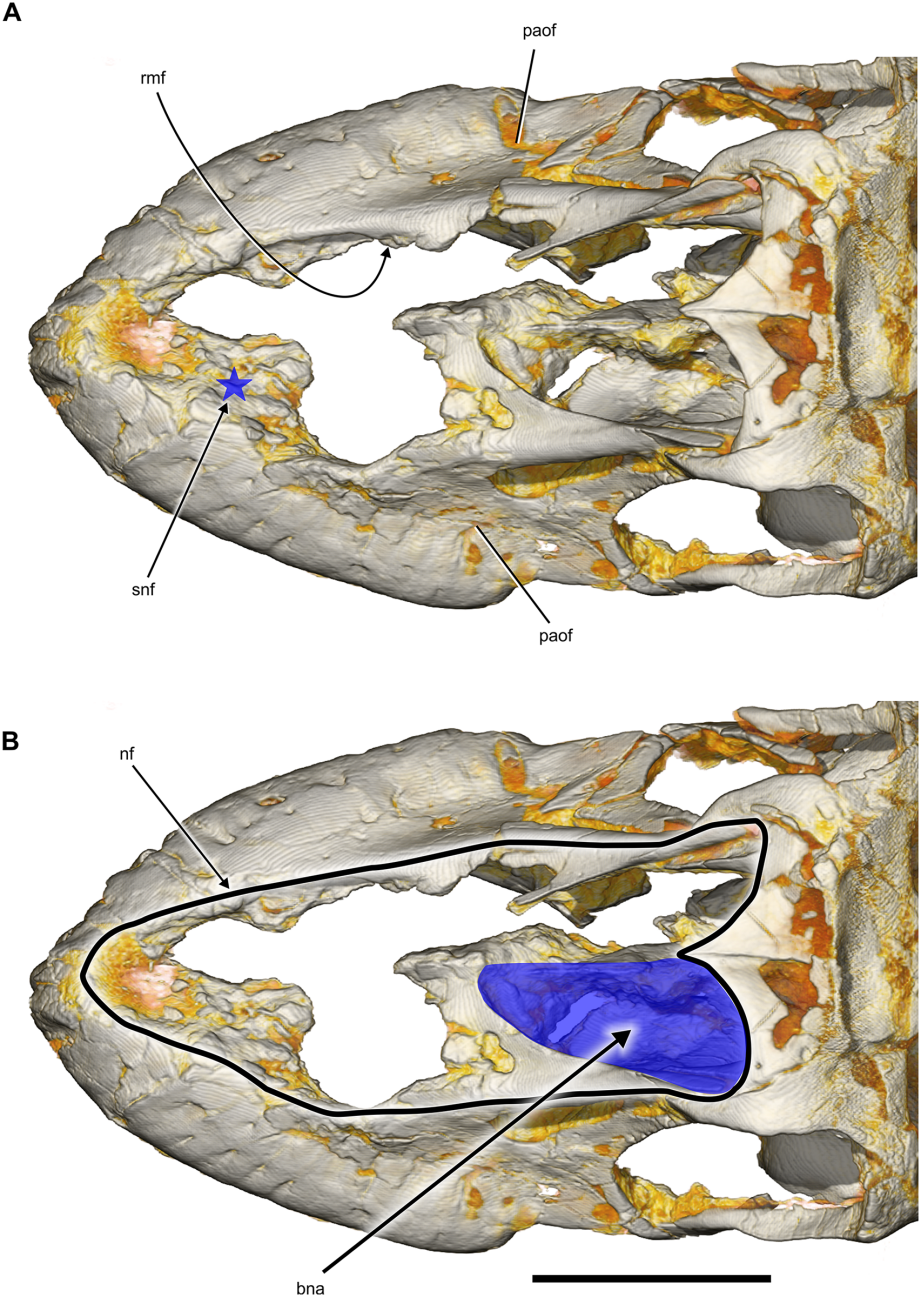
Snout and narial region of *Sarmientosaurus musacchioi* gen. et sp. nov. (MDT-PV 2). Computed tomography-based digital visualization in dorsal view, showing locations of preantorbital, rostral maxillary, and subnarial (blue star) foramina (**A**), and left bony nasal aperture (blue overlay) and narial fossa (black line) (**B**). Abbreviations see text. Scale bar = 10 cm.

Both premaxillae of *Sarmientosaurus* are preserved (Figs 3–7; S1 Fig; S1, S2, S3 and S4 Movies. They articulate rostromedially, rendering the rostral end of the snout convex in lateral view, as in *Abydosaurus*, *Europasaurus*, *Giraffatitan*, *Nemegtosaurus*, *Quaesitosaurus*, and *Tapuiasaurus*. This contrasts the morphologies in *Malawisaurus* (Jacobs et al. [102]:fig. 1a; Gomani [32]:fig. 4a) and *Narambuenatitan* (Filippi et al. [40]:fig. 4a), where the more vertical nasal process lends the rostral margin of the premaxilla a taller, straighter lateral profile. The premaxilla of *Euhelopus* seems intermediate between these convex and subvertical conditions [101]. The premaxillae of *Sarmientosaurus* continue caudodorsally to the bony nasal apertures; only the left premaxilla preserves the rostral margin of the aperture, however. Along this margin, the area of the interpremaxillary articulation shows remnants of a sagittal crest that probably corresponds to the rostroventral base of the internarial bar. The premaxillae articulate with the maxillae caudally; in life, they would presumably have also contacted the nasals caudodorsally.

The rostroventral end of the suture between the premaxilla and maxilla is clearly discernible toward the tip of the snout, far rostral to the bony nasal aperture. In *Malawisaurus* [32,102], *Narambuenatitan* [40], and probably ISI R K 27/528 [31,102], by contrast, this suture lies ventral to the rostral end of the nasal aperture, indicating that these apertures were not retracted in these titanosaurs. Nevertheless, this condition may well have varied through titanosaurian ontogeny, as unretracted bony nasal apertures are also present in the embryonic skulls from Auca Mahuevo [15–17].

Remnants of the narial fossa of *Sarmientosaurus* are evident in more dorsal areas of the premaxillary—maxillary contact. This fossa takes the form of a slight depression of the snout, the perimeter of which has been damaged by erosion. The narial fossa is better preserved on the right side, where it can be seen to reach rostrally to the region of the premaxillary—maxillary contact (Fig 7). Thus, the narial fossa was much larger than the bony nasal aperture, as is the case in most sauropods [103,104]. A comparable narial fossa is present in *Abydosaurus* and *Giraffatitan*. The lateral surface of each premaxilla is rostrocaudally short and shows small, irregular traces, some of which may be artifacts of the erosion that has affected more dorsal regions of these bones. There is a bony lamina lateral to the premaxillary teeth that is also present in the maxilla. The suture between the premaxilla and maxilla should bear the subnarial foramen, an aperture that transmitted blood vessels between the narial region and palate. This foramen is found in virtually all saurischians [105] and is enlarged in many sauropods [95,103,104]. The relevant region is not well preserved in MDT-PV 2, but it is present on the left side. Here, gaps in the preserved bone fragments indicate the likely position of the subnarial foramen (Fig 7), which would be consistent with that in other sauropods. Given that inferred position and the extent of the narial fossa, it is likely that, in life, *Sarmientosaurus* had a rostrally positioned fleshy nostril as has been reconstructed for other sauropods [104].

Each premaxilla bears four alveoli, as in all other sauropods. Medially, the ventral margin of the premaxilla exhibits a continuous ridge situated close to the teeth, which is contiguous with a similarly-positioned ridge on the maxilla.

#### Maxilla

**Fig 8:**
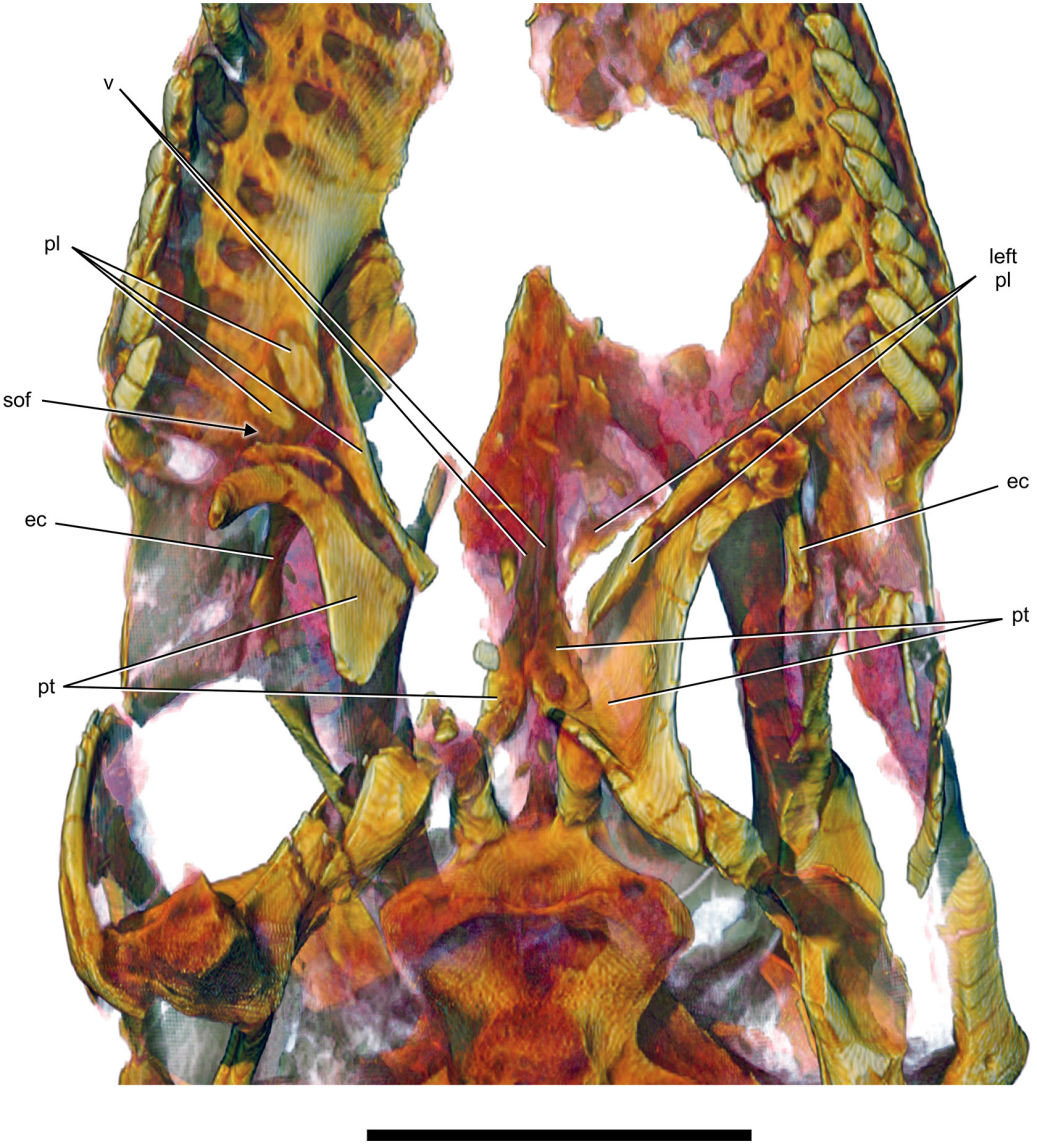
Palate of *Sarmientosaurus musacchioi* gen. et sp. nov. (MDT-PV 2). Computed tomography-based digital visualization in ventral view indicating palatal bones (ectopterygoids, palatines, pterygoids, and vomers) and the right suborbital fenestra. Abbreviations see text. Scale bar = 10 cm.

The maxilla of *Sarmientosaurus* (Figs 3–8; S1 Fig; S1, S2, S3 and S4 Movies) is a stout, rostrocaudally elongate element. Its gently convex lateral surface is pierced by neurovascular foramina that open into prominent grooves, rendering the surface slightly undulatory; these grooves are mediolaterally oriented, as in *Nemegtosaurus* [10,11] and to a lesser extent in *Giraffatitan* [95]. The holotypic maxilla fragment of *Campylodoniscus*—which, like *Sarmientosaurus*, was recovered from the Upper Cretaceous Chubut Group of southern Chubut Province—appears to be proportionally taller than that of the new taxon (see Huene [9]:82). Furthermore, *Campylodoniscus* was regarded as a *nomen vanum* by Bonaparte and Gasparini [106] and a *nomen dubium* by Powell [7]. In *Sarmientosaurus*, the ascending ramus of the maxilla forms a bar that separates the bony nasal aperture from the antorbital fenestra. The ramus arises near the rostrocaudal midline of the maxilla, further caudally than the ascending rami of *Euhelopus* [101], *Narambuenatitan*, ISI R K 27/528, UNPSJB-PV 583 (an isolated titanosaur maxilla from the Bajo Barreal Formation [53]), and especially *Rapetosaurus*, but similar to the conditions in *Abydosaurus*, *Nemegtosaurus*, and *Tapuiasaurus*.

The phylogenetic distribution of the preantorbital fenestra, a large accessory opening in the maxilla that is characteristic of Diplodocidae [107–109], was widened considerably when Wilson and Sereno [103] homologized a neurovascular foramen that occurs in various sauropods with the definitive preantorbital fenestra of diplodocids. In taxa such as *Camarasaurus*, *Europasaurus*, and *Giraffatitan*, the homologous structure is a relatively inconspicuous foramen, such that the term ‘preantorbital fenestra’ does not seem appropriate, even if the homologous foramen is elaborated into a large opening in other taxa. Although Witmer [109] and Wilson and Sereno [103] regarded the preantorbital fenestra of diplodocids as relating to the pneumaticity associated with the antorbital cavity, more recent studies [110] have suggested that the structure is vascular in origin. Derived lithostrotian titanosaurs such as *Nemegtosaurus* [11], *Rapetosaurus* [13], and *Tapuiasaurus* [14] apparently converged on diplodocids in expanding this neurovascular foramen into a relatively large opening that is here termed the preantorbital foramen.

*Sarmientosaurus* lacks a true preantorbital foramen or fenestra, but probably possesses the homologous neurovascular foramen. Criteria for establishing the homologies of these openings have not previously been established, but include the following: (1) the foramen/fenestra is located dorsal to the maxillary palatal shelf, where it communicates with the canal for the maxillary neurovascular bundle (traceable in computed tomographic [CT] scan data); (2) the foramen/fenestra is in the vicinity of the suborbital fenestra, where the palatine and ectopterygoid unite with the maxillary palatal shelf; and (3) the foramen/fenestra is generally just caudal to the alveolar tooth chamber (and this chamber, housing the replacement teeth, may extend somewhat caudal to the most distal [= caudal] erupted tooth position). Applying these criteria to *Sarmientosaurus* reveals a credible (albeit poorly preserved) foramen on each side of the cranium that we regard as the homolog of the preantorbital foramen/fenestra (Figs 3–7). This small, otherwise unremarkable opening is located ventral to the rostral end of the antorbital fenestra, similar to its position in *Giraffatitan* [95] and the Auca Mahuevo embryos [17]. In *Abydosaurus* [98] and especially *Nemegtosaurus* [11], the preantorbital foramen or its homolog is positioned more rostrally on the snout, whereas in *Euhelopus* (Wilson and Upchurch [111]:fig. 6) and an immature specimen of *Camarasaurus* (CM 11338; Wilson and Sereno [103]:fig. 7a), it is situated more caudally. In *Rapetosaurus*, the preantorbital foramen is placed comparatively rostrally, on the rostroventral base of the jugal process of the maxilla, ventrolateral to the rostral end of the greatly enlarged antorbital fenestra [13]. The extremely large preantorbital foramen of *Tapuiasaurus* is also placed rostrally relative to that of *Sarmientosaurus* [14]. The homolog of the preantorbital foramen/fenestra is reduced or absent in adult individuals of *Camarasaurus* (Wilson and Sereno [103]:46).

Wilson and Sereno (1998) highlighted another neurovascular feature in sauropods: the rostral (= anterior) maxillary foramen, which opens within the narial fossa caudal (or lateral, in diplodocids) to the subnarial foramen. In a sense, this structure is a counterpart to the preantorbital foramen/fenestra in that both are associated with the canal for the maxillary neurovascular bundle and transmitted branches thereof in life [110]. The CT scan data of *Sarmientosaurus* clearly show (especially on the right side of the cranium) the course of the maxillary neurovascular bundle through the maxilla and where this bundle gives off the branch that leads to the rostral maxillary foramen before continuing rostrally through the bone. The rostral maxillary foramen opens medially into the narial fossa just inside the rim of the fossa, near the base of the maxillary ascending ramus. As preserved, the foramen is modest in size, comparable in relative scale to that observed in *Camarasaurus* (CM 11338) and *Giraffatitan* (MB R.2223.1).

Caudally, the ventral edge of the maxilla of *Sarmientosaurus* forms a marked, roughly semicircular embayment (the ‘postdental emargination’ of Gallina and Apesteguía [24]:fig. 7) that is also present in other titanosaurians such as *Nemegtosaurus* [11], *Rapetosaurus* [13], *Tapuiasaurus* [14], and the Auca Mahuevo embryos [17], and even in the brachiosaurid *Giraffatitan* [95] and the basal macronarian *Europasaurus* [97]. The development of this structure in *Sarmientosaurus* is intermediate between the incipient conditions in *Europasaurus*, *Giraffatitan*, and *Nemegtosaurus* (see Wilson [11]:fig. 16a) and the much larger, deeper embayments of *Tapuiasaurus* and especially *Rapetosaurus*. The maxilla articulates with the jugal caudoventrally and the lacrimal and prefrontal caudodorsally. Caudal to the tooth row, it exhibits a robust caudoventral process with a jugal articulation that is more elongate than that in *Nemegtosaurus*, *Rapetosaurus*, and probably *Tapuiasaurus* [14]. The medial edge of the maxillary ascending ramus bordering the bony nasal aperture has a low but well-defined ridge that we tentatively consider an autapomorphy of *Sarmientosaurus*, although a similar or the same structure may also be incipiently developed in *Tapuiasaurus* (see Zaher et al. [14]:fig. 1c).

The medial surface of the maxilla is longitudinally concave and exhibits the same continuous bony flange that is present in the premaxilla. There are 12 teeth in the left maxilla of the *Sarmientosaurus* holotype and 11 in the right. The tooth row encompasses 64% of the length of the maxilla, a condition that is intermediate between those in *Camarasaurus* and *Giraffatitan* (75%) on one hand and *Abydosaurus* (52%), *Tapuiasaurus* (46%), and *Nemegtosaurus* (34%) on the other. The relatively long tooth row in *Sarmientosaurus* may relate to the plesiomorphic (i.e., unexpanded) condition of the homolog of the preantorbital foramen as well as the intermediate condition of the ‘postdental emargination.’ In other words, the restriction of the teeth to the rostral end of the snout in more advanced titanosaurs may be correlated with the increased development of both these features.

#### Nasal

The nasal (Figs 3–7; S1 Fig; S1, S2, S3 and S4 Movies) is a planar bone that is roughly quadrangular in dorsal view. It is rostrocaudally longest medially, at the internasal articulation, and extends rostrally as a process that presumably would have articulated with the ascending ramus of the premaxilla to form the missing internarial bar. The curved rostrolateral edge of the nasal forms the caudal border of the bony nasal aperture, and continues to expand caudally at an approximately straight lateral margin that borders the prefrontal and frontal. The caudal margin of the nasal has a straight, mediolaterally-oriented suture with the frontal. The caudal ends of both nasals are damaged near their contact with the frontals.

#### Lacrimal

The lacrimal (Figs 3–7; S1 Fig; S1, S2, S3 and S4 Movies) is a dorsoventrally elongate and gently caudodorsally-inclined bone that separates the antorbital fenestra from the orbit. As observed in rostral view, the lacrimal is oriented dorsomedially—ventrolaterally (i.e., its dorsal end is positioned slightly more medially than its ventral end in the articulated skull). The lacrimal is expanded rostroventrally at its contact with the jugal. Caudodorsally, the lacrimal articulates with the ascending ramus of the maxilla, the prefrontal, and the nasal. Its dorsal end possesses a very subtle rostral process that is comparable to those of *Giraffatitan* and *Nemegtosaurus* but much less developed than in *Abydosaurus*, *Rapetosaurus*, *Tapuiasaurus*, and especially *Bonitasaura*. The maxilla—lacrimal articulation is complex in that the ascending ramus of the maxilla is embedded in and bordered laterally and medially by the dorsal process of the lacrimal, a feature that we provisionally regard as an autapomorphy of *Sarmientosaurus*, though a similar or the same morphology may also be present in *Tapuiasaurus* (see Zaher et al. [14]:fig. 1c). Unlike in most macronarians (e.g., *Abydosaurus*, *Camarasaurus*, *Giraffatitan*, *Nemegtosaurus*, *Rapetosaurus*, and as reconstructed for *Bonitasaura*), the dorsal terminus of the lacrimal is not well exposed in lateral view due to a contact between the maxilla and prefrontal. The lateral surface of the lacrimal is relatively smooth compared to those of the premaxilla and maxilla.

#### Prefrontal

The prefrontal of *Sarmientosaurus* (Figs 3–7; S1 Fig; S1, S2, S3 and S4 Movies) is crescentic and rostrocaudally elongate in dorsal view. Its dorsal surface is convex and its ventral surface is smoothly concave. The prefrontal articulates with the maxilla and lacrimal rostrally and rostromedially, the nasal caudomedially, and the frontal caudally. The ventral surface forms the rostrodorsal margin of the orbit. Both prefrontals are well preserved, and their lateral surfaces are somewhat rugose, as in *Rinconsaurus* [45], though not nearly as much so as in *Nemegtosaurus* [11]. The prefrontal has an elongate rostral process, a feature that is absent in *Abydosaurus*, *Camarasaurus*, and *Giraffatitan* but shared with *Nemegtosaurus*, *Rapetosaurus*, and *Tapuiasaurus*; Zaher et al. [14] considered this a feature of nemegtosaurid titanosaurs. In *Sarmientosaurus*, the rostral process is triangular in dorsal view, with the concave medial margin articulating with the nasal and maxilla and the convex lateral edge forming part of the rostrodorsal sector of the orbit. The prefrontal is dorsoventrally thick laterally and becomes even thicker medially.

#### Frontal

Both frontals (Figs 3–7; S1 Fig; S1, S2, S3 and S4 Movies) have suffered strong mediolateral deformation. This, coupled with the presence of cracks and rugosities on their dorsal surfaces, precludes us from determining whether these bones are coossified or simply firmly sutured. As our CT data do not provide evidence to resolve this matter, we will describe both frontals as a single unit. Together, these bones comprise a transversely wide surface that extends between the orbits but that is much shorter in rostrocaudal dimension (transverse width to rostrocaudal length ratio equals 3.5 to 1). The frontals are bordered by the nasals and prefrontals rostrally, the parietals caudally, and the postorbitals laterally, and they also contribute to the dorsal margins of the orbits. Unlike in *Bonitasaura* [24], *Nemegtosaurus* and *Quaesitosaurus* [11], and *Rapetosaurus* [13], the orbital margin of the frontal of *Sarmientosaurus* is smooth, not ornamented. The frontal also appears to lack the rostrolateral process present in *Ampelosaurus* [18,19], the median ‘dome’ of several titanosaurs (e.g., *Antarctosaurus*, *Bonatitan*, *Bonitasaura*, *Rapetosaurus*, *Saltasaurus*, the isolated braincase MGPIFD-GR 118), and the more lateral dorsal prominences of *Bonitasaura* and *Saltasaurus* [24]. The rostral and caudal sutures of the frontals are poorly preserved; as such, those indicated in Figs 3–5 are probable but not definitive. Nevertheless, the frontals appear to be proportionally rostrocaudally shorter than those of some other titanosauriforms (e.g., *Ampelosaurus*, *Phuwiangosaurus*, *Saltasaurus*). Ventrally there is a gap, bounded laterally by the orbitosphenoids, where the aperture for the olfactory tracts opens.

#### Parietal

The boundaries of the parietal (Figs 4–6; S1 Fig; S1, S2, S3 and S4 Movies) are difficult to establish due to the extreme fusion and deformation suffered by part of the caudal region of the skull. CT data show that the central interparietal/interfrontal zone forms a single triangular surface, the truncated apex of which arises from the parietal—supraoccipital contact and the base from the nasofrontal suture. Detailed examination of the cracked dorsal surface of the skull roof allows the identification of a probable frontoparietal suture, which suggests that the parietal contributes to the supratemporal fenestra. In contrast to the conditions in most other macronarians for which this region of the skull is known (e.g., *Ampelosaurus*, *Antarctosaurus*, *Bonatitan*, *Bonitasaura*, *Camarasaurus*, *Isisaurus*, *Jainosaurus*, *Malawisaurus*, *Nemegtosaurus*, the isolated titanosaur braincases FGGUB 1007, MGPIFD-GR 118, and MML-194), the supratemporal fenestrae of *Sarmientosaurus* are separated by only a short distance, as in *Europasaurus*, *Rapetosaurus*, and especially *Giraffatitan* and USNM 5730 (a partial skull referred to *Brachiosaurus* [112]). Unlike in many of these forms (e.g., *Ampelosaurus*, *Antarctosaurus*, *Bonatitan*, *Bonitasaura*, *Jainosaurus*, *Nemegtosaurus*, MGPIFD-GR 118), the long axes of these fenestrae are oriented approximately perpendicular to the sagittal plane instead of being aligned rostromedially—caudolaterally. In these regards, *Sarmientosaurus* is intermediate between brachiosaurids (i.e., *Giraffatitan*, USNM 5730) and derived lithostrotians. This compression and reorientation of the supratemporal fenestrae coincides with the lateral reorientation of the orbits in titanosaurs, both of which presumably evolved in response to the expansion of the nasal vestibule.

There is no visible suture between the parietals, but together they have a wing-like contour comparable to that observed in *Camarasaurus* and proportionally wider than those of *Giraffatitan*, *Nemegtosaurus*, and *Rapetosaurus*. The parietal contacts the postorbital laterally, the supraoccipital caudoventrally, and the otoccipital more ventrally. There is no parietal foramen, in contrast to the condition in sauropods such as *Shunosaurus* [113] and some diplodocoids (see [114]). The parietal of *Sarmientosaurus* lacks the bizarre dorsal excrescences of the isolated Transylvanian braincase FGGUB 1007 [52].

#### Postorbital

The postorbital (Figs 3–7; S1 Fig; S1, S2, S3 and S4 Movies of *Sarmientosaurus* has the form of a caudally-reclined ‘T.’ Its thick, convex caudodorsal ramus contributes to the dorsal margins of the orbit and infratemporal fenestra. The longer ventral ramus is rostroventrally directed to contact the dorsal process of the jugal, and it forms most of the boundary between the infratemporal fenestra and orbit. Both postorbitals are well-preserved, though they have lost contact with their respective jugals; furthermore, the end of the ventral ramus of the left postorbital has rotated laterally. The lateral surface of the postorbital is fairly smooth. The ventral ramus is thickened at its rostroventral end that articulates with the jugal. Its flattened rostral surface is slightly concave in lateral view, whereas its caudolateral side is convex. The jugal articular surface of the postorbital is oriented caudomedially. The caudodorsal ramus is an expansion in the form of a convex anvil. It is pierced by a small but well-defined, caudolaterally-opening vascular foramen near the dorsal margin of the infratemporal fenestra and the dorsal base of the ventral ramus. The same or a very similar postorbital foramen occurs in the North American titanosauriform *Abydosaurus* (see Chure et al. [98]:figs. 3b, 4b; D’Emic [115], appendix 4); this structure has so far been documented only in that taxon and *Sarmientosaurus*, though it may occur in other sauropods (L.M.W., pers. obs.). There is no evidence of the ornamentation of the orbital margin of the caudodorsal ramus that occurs in *Nemegtosaurus* and *Quaesitosaurus* [10,11,43]. As best observed in rostromedial view, there are two small, probably vascular foramina situated close together at the rostroventral end of the right postorbital. Their presence on the left postorbital cannot be verified due to damage. The postorbital of *Phuwiangosaurus* differs from that of *Sarmientosaurus* in having a dorsoventrally thinner caudodorsal ramus, the rostral projection of which is much longer than its caudal counterpart (see Suteethorn et al. [41]:fig. 6). The rostral projection also appears much longer than the caudal projection in the recently described Argentinean titanosaur *Quetecsaurus* [44].

#### Jugal

Both jugals (Figs 3 and 5–7; S1 Fig; S1, S2, S3 and S4 Movies) are well preserved, but their surfaces exhibit small cracks caused by erosion. The jugal of *Sarmientosaurus* is a roughly ‘L’-shaped bone that is rostrocaudally longer and differently shaped than the jugals of most other macronarians, including *Camarasaurus*, *Euhelopus*, *Giraffatitan*, *Malawisaurus*, *Nemegtosaurus*, and *Rapetosaurus*. The jugals of *Tapuiasaurus* [14] and the taxon represented by the Auca Mahuevo embryos [17] are similarly elongate but very different in shape, being tetraradiate rather than triradiate and comparatively dorsoventrally thick. Within Titanosauriformes, the jugal of *Sarmientosaurus* most closely resembles that of *Abydosaurus*, though the caudoventral ramus is sharply pointed and considerably longer in the latter taxon (see Chure et al. [98]:fig. 3a, b). In *Sarmientosaurus*, the subvertical dorsal ramus of the jugal contacts the postorbital and separates the ventral ends of the orbit and infratemporal fenestra. The rostroventral ramus is rostrodorsally projected and has a sigmoid contact with the caudal end of the maxilla. Ventrally, near its caudal end, the rostroventral ramus makes a slight contribution to the large lateral embayment caudal to the tooth row. The jugal also forms the ventral margin of the orbit and the caudoventral corner of the antorbital fenestra, and contacts the lacrimal rostrodorsally. The dorsal and rostroventral jugal rami meet at a nearly right angle. The rostroventral ramus is laminar throughout its extent, and is dorsoventrally expanded at its contact with the lacrimal. More caudally, between the orbit and the caudalmost sector of the maxilla, the rostroventral ramus of the jugal narrows and intersects the dorsal ramus. The caudoventral ramus of the jugal is much shorter than the rostroventral ramus. The jugal has been disarticulated from the postorbital on both sides of the skull; specifically, the articular end of the right postorbital is free and has been displaced laterally relative to the dorsal articular end of the jugal, and part of the postorbital overlaps the jugal on the left side. The articular surface of the right postorbital is rostromedially oriented and longitudinally twisted, suggesting that rostrocaudal pressures suffered by the skull during diagenesis have caused the bilateral displacement between the postorbitals and jugals.

#### Squamosal

The squamosal (Figs 3, 4 and 6; S1 Fig; S1, S2, S3 and S4 Movies) is a rostrocaudally elongate and dorsoventrally oriented bone. Along with the quadratojugal, it forms most of the caudoventral margin of the infratemporal fenestra. Whether or not the squamosal participates in the supratemporal fenestra is not totally clear, although it probably does not given the seemingly considerable distance between these structures. The squamosal is excluded from the supratemporal fenestra in *Phuwiangosaurus* [41] and the derived titanosaurs *Nemegtosaurus*, *Quaesitosaurus*, and *Tapuiasaurus*. In *Sarmientosaurus*, the squamosal articulates with the quadratojugal ventrally, the postorbital caudodorsally, and the quadrate medially. Its sutural contacts are not clearly delimited, despite the fact that the right squamosal is fairly well preserved; the left is damaged laterally and caudally. The lateral surface of the right squamosal is fractured and cracked. The main body of the squamosal is flexed, forming a rostromedially-oriented convexity near its contact with the postorbital. This convexity divides two regions: a long rostrolateral sector that is wide and laminar at its contact with the quadratojugal, and a shorter caudomedial sector that has a concave surface and that is bordered medially by the quadrate. The squamosal is slightly sigmoid in lateral view.

#### Quadratojugal

The right quadratojugal (Figs 3–6; S1 Fig; S1, S2, S3 and S4 Movies) is preserved. It is an ‘L’-shaped bone with a dorsal process that is angled slightly caudally and that is shorter than the ventral process; the latter is directed rostrodorsally to contact the jugal. The quadratojugal forms the rostroventral border of the infratemporal fenestra. It articulates with the jugal rostrodorsally, the squamosal caudodorsally, and the quadrate medially. Its contact with the squamosal is difficult to define due to fracturing of the region in question, although that contact clearly occurs in a rostromedial plane. The rostral section of the ventral ramus that contacts the jugal is slightly cracked. A small bone fragment adhered to the lateral surface of the caudodorsal part of the coronoid eminence of the right mandibular ramus appears to be the rostralmost tip of the ventral ramus of the right quadratojugal. In contrast to *Nemegtosaurus* [10,11], *Quaesitosaurus* [43], and especially *Tapuiasaurus* [14], the ventral edge of the ventral ramus is uniformly convex rather than sinuous in lateral view, and its rostral end is not ventrally expanded. In *Sarmientosaurus*, there may have been some rostral displacement of the quadratojugal relative to the jugal, but if so, it does not appear to have been significant. As observed in ventral view, the ventral process of the quadratojugal is projected dorsomedially, forming a concave medial surface. In caudal view, the subvertical dorsal ramus of the ‘L’ comprises the lateral border of the quadrate fossa; ventrally, this same margin is caudomedially projected as a ‘tongue-like’ process that caudally overlaps the quadrate. In *Tapuiasaurus*, by contrast, this process is not present, but the quadratojugal of that Brazilian titanosaur does possess a well-developed, acute caudoventral flange that does not occur in *Sarmientosaurus* [14]. The tongue-like process appears to be absent in other macronarians (e.g., *Camarasaurus*, *Giraffatitan*, *Nemegtosaurus*, *Quaesitosaurus*) as well; as such, we regard it as an autapomorphy of *Sarmientosaurus*.

### Palatal Complex

The palatal region of the *Sarmientosaurus* holotype was partially damaged by erosion, mainly on its midline. The vomers are incomplete, as is often the case in sauropod skulls [96], and parts of the palatines, ectopterygoids, and pterygoids are also missing.

#### Palatine

Although both palatines are incomplete, the right is better preserved than the left (Figs 3, 5, 6 and 8; S1 Fig; S1, S2, S3 and S4 Movies). The right palatine preserves part of the lateral region, primarily the elongate, rostrolaterally-directed maxillary process. The entire medial section of the bone where it articulates with the pterygoid has been lost, whereas the left palatine preserves most of the pterygoid contact. The body of the maxillary process is dorsomedially inclined and roughly tubular. Its rostrolateral contact with the ectopterygoid is preserved, as is its more caudolateral contact with the rostral end of the pterygoid, although all of these bones are somewhat disarticulated. The rostral end of the maxillary process is fractured into pieces on both sides, but enough is preserved to suggest that its contact with the palatal process of the maxilla is typical for sauropods in that the palatine underlaps the maxilla ventrally. Likewise, the arrangement of the maxillary process of the palatine, the ectopterygoid, and the pterygoid around the suborbital (= postpalatine) fenestra also resembles that of other sauropods in that this fenestra is small and bounded rostrally by the palatine, caudally by the ectopterygoid (with the pterygoid nearby), and laterally by the palatal process of the maxilla. The maxillary process of the palatine narrows and expands again caudomedially as it approaches the pterygoid. As shown on the left side, although somewhat damaged and disarticulated, the pterygoid contact of the palatine is expanded and articulates in the fork between the medial vomerine ramus and the lateral transverse ramus of the pterygoid, as is common in sauropods [95,96,103].

#### Pterygoid

The pterygoid (Figs 3, 5, 6 and 8; S1 Fig; S1, S2, S3 and S4 Movies) is the largest bone of the palatal complex. Neither pterygoid is complete, but enough is preserved of both to offer a reasonably comprehensive description. In general, the pterygoid of *Sarmientosaurus* is typical for sauropods in that the bone has a complex shape, with three main processes—the quadrate, vomerine, and transverse rami—arising from the highly arched body. The pterygoid body, which is better preserved on the left side, is expansive, forming a distinct ventral fossa (the postchoanal fossa) that faces rostromedially. The quadrate ramus, also better preserved on the left pterygoid, branches off the caudolateral corner of the body and twists into a more vertical plane as it attaches to the medial aspect of the reciprocal pterygoid ramus of the quadrate. The vomerine ramus passes dorsomedially, contacting its counterpart at the midline to form a thin triangular wedge that approaches the roof of the nasal cavity, where it nearly contacts the subnarial processes of the premaxilla and maxilla. Rostrally, the vomerine rami pass medial to the paired vomers. Near the juncture of the vomerine and quadrate rami, the body of the pterygoid forms a shallow but distinct, caudomedially facing pocket for the articulation of the basipterygoid process.

The transverse ramus of the pterygoid is preserved on both sides, but is better preserved on the right side. As is true for most sauropods, the transverse ramus is slender and swept far rostrally, carrying the ectopterygoid with it. The lateral end of the transverse ramus is slender and curves ventrally. The ectopterygoid attaches broadly to its rostral surface, just caudal to the suborbital fenestra. Together, these two bones form the ‘pterygoid flange,’ which is a strong, transverse projection in many other archosaurs, but is a relatively delicate structure in *Sarmientosaurus* and most other sauropods. As noted above, the palatine articulates with the body of the pterygoid rostrally, between the vomerine and transverse rami of the latter.

#### Vomer

The vomers (Figs 5, 6 and 8; S1 Fig; S1, S2, S3 and S4 Movies) are delicate, paired bones that are damaged and best observed in the CT images. They are thin laminae of bone that attach to the lateral and ventral portions of the vomerine rami of the pterygoids. As noted above, along with the pterygoids, they likely contacted the ventral surfaces of the subnarial processes of the premaxillae and maxillae. This is also the case in *Camarasaurus* and *Diplodocus*, and undoubtedly in other sauropods as well, though this part of the cranium is not well understood in most taxa.

#### Ectopterygoid

The ectopterygoids (Figs 5, 6 and 8; S1 Fig; S1, S2, S3 and S4 Movies) are essentially complete but remain largely embedded in matrix. Of the two, the right is the more clearly visible. The ectopterygoid is a relatively simple, slender bone that forms the caudal border of the suborbital fenestra. Its lateral end contacts the medial surface of the maxilla. The rostral end of the ectopterygoid passes ventrally and curves to articulate on the rostral face of the transverse ramus of the pterygoid, such that they collectively form the ‘pterygoid flange,’ as noted above. The lateral, medial, and ventromedial surfaces of the ectopterygoid are smooth. The ectopterygoid of *Sarmientosaurus* is much shorter rostrocaudally than the bones that Wilson [11] identified as ectopterygoids in *Nemegtosaurus* and *Quaesitosaurus*.

#### Quadrate

The right quadrate is, in most regions, better preserved than the left (Figs 3–6 and 8; S1 Fig; S1, S2, S3 and S4 Movies). It articulates with the pterygoid rostromedially, the squamosal caudodorsally, the quadratojugal laterally, and the articular ventrally, forming the jaw joint. Although the rostroventral region of both quadrates is damaged, both preserve at least part of the articulation with the pterygoid, which is better preserved on the left side. On the right quadrate, caudally, the edge of the vertical lamina that articulates laterally with the quadratojugal and ventrally with the articular is eroded. The right quadrate is fairly smooth in areas where its surface is intact. Its long axis is oriented caudodorsally relative to that of the skull. The quadrate is obscured from rostral view by the other bones it contacts. It is best observed in caudal view, where the damaged head that articulates with the squamosal rostrolaterally may be seen at its dorsal end. Other structures of the right quadrate evident in caudal view include the subvertical and plate-like medial edge, the thick expansion that terminates ventrally in the mandibular articulation, and the quadrate fossa, which is delimited medially by the quadrate and laterally by the quadratojugal. The quadrate fossa of *Sarmientosaurus* faces caudolaterally, as in *Nemegtosaurus*, *Quaesitosaurus*, and *Rapetosaurus*, though it is apparently not as laterally oriented as it is in these taxa. The quadrate fossa is comparable in shape to those of most other titanosaurs (e.g., *Narambuenatitan*, *Nemegtosaurus*, *Quaesitosaurus*, *Rapetosaurus*), but is seemingly more dorsoventrally elongate than in *Phuwiangosaurus* [41] and especially *Malawisaurus*; in the latter African titanosaur, the quadrate fossa appears nearly as wide mediolaterally as it is tall (see Gomani [32]:fig. 5d). The ventral extreme of the right quadrate preserves part of the medial condyle, which is mediolaterally wider than that of *Narambuenatitan* (see Filippi et al. [40]:fig. 5a). The pterygoid ramus of the right quadrate and its damaged contact with the pterygoid are also evident in caudal view. The pterygoid ramus is mediolaterally thick. The quadrate—squamosal articulation is laterally expanded. The part of the quadrate that is situated more caudally than the squamosal is visible in lateral view. According to Zaher et al. [14], caudolateral exposure of the quadrate is a feature of nemegtosaurids; its occurrence in *Sarmientosaurus* broadens the distribution of this character to include more basal titanosaurians as well. In overall morphology, the quadrate of the new Patagonian taxon is similar to those of *Quaesitosaurus* and *Rapetosaurus*.

### Braincase

The braincase of *Sarmientosaurus* is preserved in natural articulation to the remainder of the cranium. Some regions are in good condition, such as the supraoccipital, which is only slightly damaged. The otoccipitals (fused exoccipital—opisthotic complexes), by contrast, are damaged laterally, having lost some of the paroccipital processes. Some areas of the surface of the laterosphenoid—orbitosphenoid complex are weathered and obscured by sediment, and the basal tubera are eroded. Only the base of the occipital condyle is preserved. Despite this surficial damage, the internal structure of the endocranial cavity is well preserved, and is discussed below (see “Cranial Endocast”).

#### Supraoccipital

The supraoccipital (Figs 4–6; S1 Fig; S1, S2, S3 and S4 Movies) is a massive, subhexagonal element that is exposed only in caudal, dorsal, and ventral views. It exhibits a prominent sagittal nuchal crest that, in caudoventral view, resembles a large spool flanked by two wing-like sectors. The supraoccipital is bordered by the parietals rostrodorsally and laterally and the otoccipitals ventrally, forming the dorsal margin of the foramen magnum. The surface of the supraoccipital is fairly smooth. The caudodorsal end of the sagittal nuchal crest displays a marked bony flange that is damaged. There is a smooth triangular depression in the caudoventral region of the crest, the apex of which reaches half the dorsoventral height of the crest. The wings of the supraoccipital are markedly longitudinally concave and slightly elevated at their lateral contact with the parietals. The ventral contact with the otoccipital is aligned dorsolaterally—ventromedially. In caudal view, the supraoccipital of *Sarmientosaurus* resembles those of *Camarasaurus* and *Giraffatitan* in its general form and relationships to other bones. In *Nemegtosaurus*, the sagittal nuchal crest is much smaller and shaped differently, but the wing-like sectors are similar to those of the new Patagonian taxon. The protuberance at the dorsal end of the sagittal nuchal crest (= the ‘supraoccipital protuberance’ of García et al. [59] and Filippi et al. [40], among others) of *Antarctosaurus*, *Bonatitan*, *Jainosaurus*, *Malawisaurus*, *Narambuenatitan*, *Rapetosaurus*, and the isolated Patagonian titanosaur braincases MML-194 and MUCPv-334 is prominent and mound-like in caudal view. In *Sarmientosaurus* and *Isisaurus* [27], conversely, this protuberance appears more quadrangular, and in *Ampelosaurus* it is only weakly developed [18,19]. The supraoccipital protuberance of *Phuwiangosaurus* seems unusually narrow transversely [41]. The supraoccipitals of *Bonatitan*, *Rapetosaurus*, MML-194, and probably *Muyelensaurus* (see Calvo et al. [39]:fig. 4) have a midline groove on their caudal aspect, a structure that is absent in other titanosaurs.

#### Otoccipital (= exoccipital—opisthotic)

The exoccipital and opisthotic (Figs 4–6; S1 Fig; S1, S2, S3 and S4 Movies) are fused, as in other sauropods [96,113,116] and indeed in most other diapsids, such that this compound element is often termed the otoccipital. The surface of this complex is marked by small cracks and grooves that obscure its margins in some areas, although the CT images provide clear information in this respect. The surface of the caudal section is pitted by erosion. The otoccipitals are massive, and extend as paired wing-like, mediolaterally elongate and gently arched paroccipital processes laterally flanking the foramen magnum, which is circumscribed by a marked ridge. This lateral elongation is comparable to that present in many titanosauriforms (e.g., *Ampelosaurus*, *Bonatitan*, *Giraffatitan*, *Jainosaurus*, *Malawisaurus*, *Muyelensaurus*, *Narambuenatitan*, *Quaesitosaurus*, *Rapetosaurus*), but differs from the conditions in *Antarctosaurus*, *Nemegtosaurus*, and *Saltasaurus*, in which the ventral margins of the medial bases of the paroccipital processes angle more steeply ventrolaterally. The otoccipital is bordered by the laterosphenoid rostromedially, the prootic rostroventrally, the supraoccipital dorsally, the parietals dorsolaterally, and the basioccipital ventrally. The opisthotic portion of the bone forms a smooth concavity approaching the prootic; the contact between these bones on the right side of the skull is a sinuous, dorsoventrally-oriented suture. A prominent feature of the otoccipital is the metotic fissure (= vagal, jugular foramen), which is semilunar in contour and constitutes the largest opening on the lateral wall of the braincase. The glossopharyngeal, vagus, and accessory nerves (cranial nerves IX–XI) all would have traversed the metotic fissure. The fenestra cochleae (round window of the inner ear; see below) opens into the metotic fissure about halfway along the length of the latter. The fenestra vestibuli (oval window of the inner ear) is just rostrodorsal to the metotic fissure, between the prootic and the opisthotic portion of the otoccipital.

Dorsal to the metotic fissure, the otosphenoidal crest (= crista prootica) is present as a sharp ridge. The ventralmost edge of the wing of the otoccipital caudally defines a semilunar recess on either side of the foramen magnum. The foramen magnum is ovate and taller than wide, as in many macronarians (e.g., *Antarctosaurus*, *Bonatitan*, *Camarasaurus*, *Giraffatitan*, *Jainosaurus*, *Nemegtosaurus*, *Pitekunsaurus*, *Quaesitosaurus*, *Vahiny*), but not *Malawisaurus*, *Muyelensaurus*, *Narambuenatitan*, *Rapetosaurus*, and *Saltasaurus*, in which these dimensions are subequal, and *Ampelosaurus*, in which the foramen magnum is subtriangular and slightly wider than tall [18,19]. On both sides of the foramen magnum there are two openings that transmitted branches of cranial nerve XII (the hypoglossal nerve), a condition that also occurs in more basal sauropods such as *Spinophorosaurus* [117], *Camarasaurus* [118], and *Giraffatitan* [95,119]. Only one hypoglossal foramen is reported in most titanosaurs, such as *Ampelosaurus* sp. [19], *Bonatitan* [20,22], *Rapetosaurus*, MGPIFD-GR 118 [58], and MCF-PVPH-765 [60], and the isolated titanosauriform braincase TMM 40435 [120], although two hypoglossal foramina per side are present in CCMGE 628/12457, an isolated titanosaur braincase from the Upper Cretaceous of Uzbekistan [63]. No proatlantal facets are evident lateral to the foramen magnum of *Sarmientosaurus*, in contrast to the condition in two braincases referred to *Isisaurus* [27,29] and a generically indeterminate titanosaur braincase from the Maastrichtian of France [50].

#### Prootic

The left prootic is better preserved than the right (Fig 6; S1 Fig; S1, S2, S3 and S4 Movies). As noted by Madsen et al. [96], the prootic is one of the most difficult cranial bones to study in sauropods—especially in an articulated skull—due to its location in the braincase and the difficulty in defining its boundaries with surrounding bones. The prootic of *Sarmientosaurus* is laterally positioned in the braincase. Caudally, it borders the supraoccipital and the opisthotic portion of the otoccipital, but the suture with the former is not visible. A foramen for the facial nerve can be seen on both sides, at the approximate midpoint of the otosphenoidal crest. Medially, dorsal to the metotic fissure, there is a groove for the maxillary branch of the trigeminal nerve that courses rostrocaudally through a large foramen. Immediately caudal to the maxillary nerve foramen is the foramen for the mandibular branch of the trigeminal nerve. Medially, the trigeminal nerve foramen is a single aperture between the prootic and laterosphenoid, but laterally it branches such that maxillary and mandibular nerves emerge through separate foramina. Moreover, the ophthalmic branch of the trigeminal nerve also diverges within the braincase wall to emerge within the laterosphenoid. Thus, *Sarmientosaurus* is presently unique within Sauropoda in having separate foramina, bilaterally, for all three branches of the trigeminal nerve, as is discussed further in the “Cranial Endocast” section below. The prootic articulates with the basisphenoid and basioccipital ventrally and the parietal dorsally, but its precise margins cannot be determined in these areas. The rostrodorsal surface of the prootic is the best exposed; it is pockmarked and traversed by cracks. The rostral section of the bone is laminar and relatively wide. The otosphenoidal crest caudolaterally defines a recess which lies dorsal to the metotic fissure of the otoccipital.

#### Laterosphenoid—orbitosphenoid

The suture between the laterosphenoid and orbitosphenoid (Figs 3, 4 and 6; S1 Fig; S1, S2, S3 and S4 Movies) is not easily visible, as is often the case in sauropods [96], but is marked by a line of foramina (see below), as in most sauropods [118] and indeed most other dinosaurs [121]. The laterosphenoid is situated caudolateral to the orbitosphenoid, and both bones contact the frontal dorsally. The laterosphenoid is bordered by the otoccipital caudally and the prootic caudally and ventrally, whereas the orbitosphenoid contacts the basisphenoid rostroventrally. In rostral view, the laterosphenoid forms a slightly concave, wing-shaped lamina, and its caudolateral margin comprises the crista antotica. Rostromedially, each laterosphenoid is continuous with its corresponding orbitosphenoid. The rostromedial union of the paired orbitosphenoids forms the ventrally sharp, somewhat sigmoid convergent rostral end of the braincase. The slightly curved section of the rostral end of the conjoined orbitosphenoids accommodates the rostral face of the dorsal end of the hypophyseal fossa. Dorsally, this rostral surface of the orbitosphenoid gradually expands to form the aperture for the olfactory tract (although this area is covered by sediment, the structures are visible in the CT data; see “Cranial Endocast” section below). Near the rostral vertex of the orbitosphenoids, the foramen for the optic nerve (cranial nerve II) is visible in rostral and lateral views. As noted above, the suture between the orbitosphenoid and laterosphenoid is marked by a row of foramina, which transmitted, from dorsal to ventral, the orbitocerebral vein, trochlear nerve, oculomotor nerve, and abducens nerve (see “Cranial Endocast” section below). Caudally, at the height of the oculomotor nerve foramen and at the contact between the laterosphenoid and prootic, there are a series of openings that were described above (with the prootic) that pertain to the three branches of the trigeminal nerve; the margins of these foramina are poorly defined due to erosion, but the canals are clear and present on both sides in the CT data. Dorsal to the trigeminal nerve foramen, and in approximately the same plane as the trochlear nerve foramen, there is another foramen that corresponds to the canal for the transversotrigeminal (rostral middle cerebral) vein.

#### Basicranium

The basicranium (Figs 4–6 and 8; S1 Fig; S1, S2, S3 and S4 Movies) forms the floor of the braincase, and consists of the coossified basioccipital, basisphenoid, and parasphenoid. Rostrally, the basioccipital articulates with the otoccipital and is fused with the basisphenoid. The neck of the occipital condyle has been preserved, and it suggests that the condyle was dorsoventrally flattened and roughly trapezoidal in section. The condyle appears to have been substantially wider than the foramen magnum, as in many macronarians (e.g., *Antarctosaurus*, *Camarasaurus*, *Isisaurus*, *Jainosaurus*, *Lirainosaurus*, *Muyelensaurus*, *Narambuenatitan*, *Nemegtosaurus*, *Phuwiangosaurus*, *Quaesitosaurus*, *Tambatitanis*, *Vahiny*, MGPIFD-GR 118); this condition is especially pronounced in *Giraffatitan*. In several titanosaurs (e.g., *Ampelosaurus*, *Bonatitan*, *Malawisaurus*, *Pitekunsaurus*, *Rapetosaurus*, MML-194), by contrast, the occipital condyle and foramen magnum appear subequal in width. When the skull roof of *Sarmientosaurus* was held horizontally, the occipital condyle projected caudoventrally, which is consistent with evidence from the inner ear (see “Cranial Endocast” section below) that suggests that the alert posture of the head was with the snout pointing strongly downward (see below). A similarly downward orientation of the head has also been reconstructed for some diplodocoids [118,122,123].

The basal tubera of the basioccipital are thick, as in *Bonatitan*, *Camarasaurus*, *Giraffatitan*, *Rapetosaurus*, the isolated titanosaurian braincase MML-194 [59], and most other macronarians, in sharp contrast to their rostrocaudally thin, sheet-like morphology in *Saltasaurus* [7,46]. They lack the peculiar ‘notch’ and pendant lateral process present in *Jainosaurus* [28,30] and *Vahiny* [48], and the foramen found in *Lirainosaurus* [35]. The condylar region is linked to the basal tubera by two thick, low, ventrolaterally-oriented ridges that delimit a deep, roughly quadrangular space that García et al. [59] termed the subcondylar recess. A similar condition appears to occur in *Malawisaurus* (Gomani [32]:fig. 6a), *Muyelensaurus* (Calvo et al. [39]:fig. 4), and the Uzbekistan titanosaur CCMGE 628/12457 [63]. Conversely, the subcondylar recess is shallow or absent in *Jainosaurus*, *Lirainosaurus*, *Mongolosaurus* [38], *Saltasaurus*, *Tambatitanis*, *Vahiny*, and MUCPv-334 [57]. No small pits or foramina are evident between the basal tubera of *Sarmientosaurus*, in contrast to the conditions in *Mongolosaurus*, *Nemegtosaurus*, *Phuwiangosaurus*, *Quaesitosaurus*, *Rapetosaurus*, *Saltasaurus*, *Tambatitanis*, and MUCPv-334. The basal tubera are united rostrally by a thinner, curved lamina, with a dorsally-directed concavity defining the hypophyseal fossa as a dome-shaped pit. Unlike those of *Rapetosaurus*, the basal tubera are not separated by a deep, V-shaped notch [13]. They diverge at the midline at an angle of approximately 35°, as in *Giraffatitan*, *Bonatitan*, MML-194, and other titanosauriforms. As in other lithostrotians [38,115,124], their combined width is clearly much greater than (approximately twice) that of the occipital condyle, although only the neck of the latter is preserved. In contrast to *Nemegtosaurus*, *Rapetosaurus*, and *Tapuiasaurus*, however, the basal tubera of *Sarmientosaurus* do not contact the quadrates. In overall morphology, they seem to most closely resemble those of the slightly younger northern Patagonian titanosaur *Muyelensaurus* (see Calvo et al. [39]:fig. 4).

The basipterygoid processes are rostroventrally projected, proportionally slightly mediolaterally expanded, and subcylindrical in cross section. Their bases are positioned well rostral to the basal tubera, and are ‘set off’ from these latter structures by the well-developed lamina that connects the tubera. The canals for the cerebral carotid arteries enter the braincase at the base of the basipterygoid process, just rostral to the basal tuber. The basipterygoid processes are subparallel, as in *Malawisaurus* [32], *Muyelensaurus* [39], *Nemegtosaurus*, *Phuwiangosaurus* [41], *Pitekunsaurus* [42], *Quaesitosaurus* [11], *Rapetosaurus*, *Tambatitanis* [47], and probably MUCPv-334 [57], unlike the conditions in *Antarctosaurus*, *Bonatitan*, *Camarasaurus*, *Giraffatitan*, *Jainosaurus*, and probably *Vahiny*, in which these processes diverge more widely. The dorsal bases of the basipterygoid processes of *Sarmientosaurus* are closely spaced, separated only by a distance approximately equal in transverse width to one of these processes, as in *Lirainosaurus* [35], *Malawisaurus*, *Mongolosaurus* [37,38], *Phuwiangosaurus*, *Rapetosaurus*, and probably MUCPv-334. In *Antarctosaurus*, *Camarasaurus*, *Giraffatitan*, *Muyelensaurus*, *Narambuenatitan*, *Pitekunsaurus*, *Tambatitanis*, *Vahiny*, and MML-194, by contrast, the bases of these processes are placed further apart. The basipterygoid processes of *Sarmientosaurus* are much longer than their strikingly short counterparts in ISI R 467, a titanosaur braincase from India (see Chatterjee and Rudra [28]:fig. 12) that may be referable to *Isisaurus* [29], and much narrower than the unusually broad, plate-like basipterygoid processes of *Saltasaurus* [7,46]. Unlike in *Rapetosaurus* and *Tapuiasaurus*, there is no midline sagittal crest between the basipterygoid processes of *Sarmientosaurus*.

As in most diapsids, the parasphenoid of *Sarmientosaurus* is completely fused to the basisphenoid. The cultriform process of the parasphenoid is damaged and largely obscured by matrix but is present as a vertical median plate of bone located rostral to the pituitary fossa. There is an elongate ventral recess between the two basipterygoid processes at the base of the cultriform process.

### Cranial Endocast

**Fig 9:**
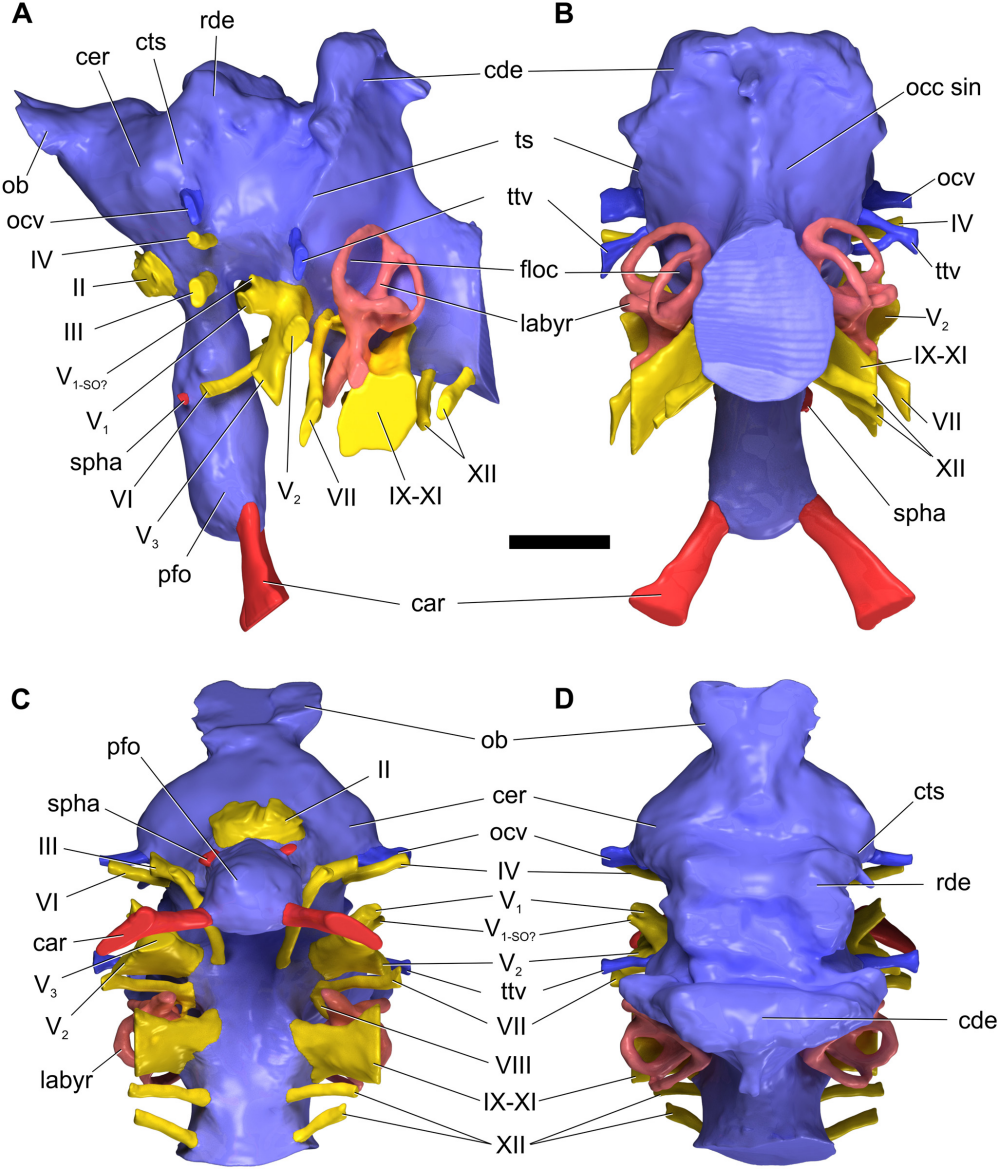
Reconstructed endocranial soft-tissues of *Sarmientosaurus musacchioi* gen. et sp. nov. (MDT-PV 2). Computed tomography-based digital visualization in left lateral (**A**), caudal (**B**), ventral (**C**), and dorsal (**D**) views. Color coding is as follows: endocast, lighter blue; endosseous inner ear labyrinth, pink; cranial nerves, yellow; arterial structures, red; venous structures, darker blue. Abbreviations see text. Scale bar = 2 cm.

The CT scan data of the *Sarmientosaurus* type specimen provided excellent discrimination of bone and matrix, allowing one of the most complete 3D reconstructions of endocranial anatomy yet produced for any sauropod (Fig 9; S1 Fig; S5 Movie). The cranial endocast, all cranial nerves, and the endosseous labyrinth were recovered from both sides of the specimen. To facilitate discussion, we will refer to the digital casts of structures as if they were the structures themselves (e.g., “orbitocerebral vein” versus “digital cast of orbitocerebral vein”).

**Fig 10:**
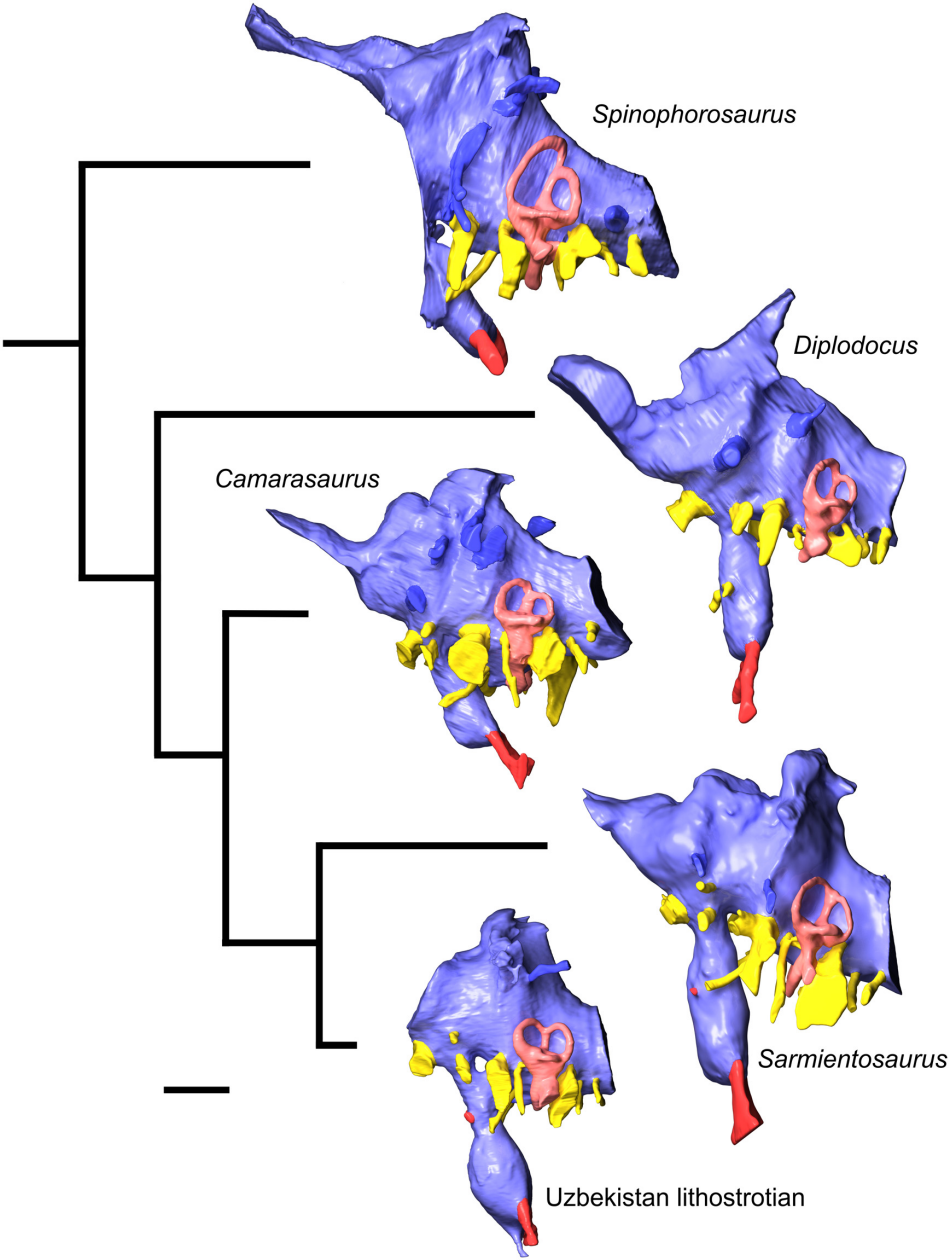
Comparison of endocranial soft-tissues of *Sarmientosaurus musacchioi* gen. et sp. nov. (MDT-PV 2) and other sauropods in a phylogenetic context. Images are computed tomography-based digital visualizations in left lateral view. Species are, from top: the basal eusauropod *Spinophorosaurus nigerensis* (GCP-CV-4229; modified from Knoll et al. [117]); the derived diplodocoid *Diplodocus longus* (CM 11161, modified from Witmer et al. [118]); the basal macronarian *Camarasaurus lentus* (CM 11338, modified from Witmer et al. [118]); the basal lithostrotian titanosaur *Sarmientosaurus musacchioi* (MDT-PV 2); and an unnamed, derived lithostrotian titanosaur from Uzbekistan (CCMGE 628/12457, modified from Sues et al. [63]; this is missing the rostral portion of the endocast). Color coding is as follows: endocast, lighter blue; endosseous inner ear labyrinth, pink; cranial nerves, yellow; arterial structures, red; venous structures, darker blue. Scale bar = 2 cm.

The endocast of *Sarmientosaurus* is in many ways intermediate between those of basal titanosauriforms such as *Giraffatitan* [95] and those of more derived titanosaurians [19,21,30,63], yet it also presents features not seen elsewhere in Sauropoda. Viewed laterally, the endocast has the typical pontine flexure seen in other saurischians, which is intermediate between the more marked flexure of basal sauropods such as *Spinophorosaurus* [117] and non-titanosaurian macronarians such as *Camarasaurus* [118] and *Giraffatitan* [95] and the almost horizontal conformation (i.e., reduced pontine flexure) seen in more advanced titanosaurs such as *Ampelosaurus* sp. [19] and *Jainosaurus* [30] (Fig 10). Two rostral swellings on the endocast represent the olfactory bulbs (Fig 9A and 9D). The olfactory tract in *Sarmientosaurus* is reduced relative to that of *Giraffatitan*, which probably reflects differences in skull morphology (e.g., in the size and position of the nasal cavity, orbit, and adductor chamber) rather than significant differences in neurology. Caudal to the olfactory bulbs and tracts is the region housing the cerebral hemispheres, which must have been modest in size.

Details of the cerebrum and other brain regions are obscured by the overlying network of dural venous sinuses, which, as in other sauropods, are extensive (Fig 9). Dorsally, there is a large rostral dural expansion [118] similar to that of *Giraffatitan* [95]. In advanced titanosaurs [19,21,30], the rostral dural expansion tends to be comparatively reduced and less globular. In *Sarmientosaurus*, it is continuous with a prominent swelling that courses ventrolaterally to terminate at the orbitocerebral vein, representing an anastomosis between the encephalic and orbital (ophthalmic) veins. These relationships suggest that this swelling likely represents the cerebrotectal (= sphenoparietal) venous sinus (Fig 9), which in extant archosaurs passes between the cerebrum and optic tectum (= optic lobe [118]).

The endocast is constricted caudal to the cerebrum by the medial pillars of the laterosphenoid. The optic tectum likely resided in this region, since, in extant sauropsids, the optic tectum is approximately bounded by the cerebrotectal sinus and transverse sinus system. Caudal to this constriction, there is a straight, ridge-like feature of the endocast between the laterosphenoid and prootic, representing the transverse sinus (= middle cerebral) venous system, as in some derived titanosaurs. The transversotrigeminal (= rostral middle cerebral) vein exits the endocranium at the lateral terminus of the transverse sinus in association with the trigeminal nerve, as in other sauropods [95,117,118] and sauropsids more generally [121,125–129]. On the right side, the transversotrigeminal vein has two trunks that externally unite in a common foramen.

The transverse sinus is continuous dorsally with the caudal dural expansion. This expansion in *Sarmientosaurus* resembles that of *Camarasaurus* [118] and *Giraffatitan* [95] in being relatively large, transversely expanded, and flared dorsolaterally, such that it is separated from the rostral dural expansion by a marked saddle (Fig 10). This condition is variable in other titanosaurs, in which the caudal dural expansion is sometimes more rounded dorsally [21] rather than flared dorsolaterally [63]. In *Sarmientosaurus*, the caudal dural expansion receives a short median venous channel (presenting the appearance of a triangular spike on the endocast) that presumably was a diploic vein draining the supraoccipital bone. Neither the rostral nor the caudal dural expansions seem to breach the skull roof dorsally (to form frontoparietal or postparietal foramina/fontanelles), although they are quite close, being roofed over only by relatively thin bone. Indeed, the presence of such frontoparietal and postparietal foramina/fontanelles is highly variable in sauropods, and some of these structures may even be preservational or preparation artifacts due to the loss of what is often very thin bone [95,118,130]. The occipital dural venous sinus and the dorsal longitudinal sinus dominate the endocast caudal to the caudal dural expansion, obscuring the form of the cerebellum.

The cerebellum should reside more or less caudal to the transverse sinus [118], although, as noted, the shape of the cerebellum is not clearly represented by the endocast. There is a very slight expansion of the cerebellar region on both sides into the ring bounded by the rostral semicircular canal that might pertain to the floccular (= auricular) lobes of the cerebellum. Floccular lobes are present in a range of archosaurs [129,131–133] but have rarely been identified in sauropods, with *Nigersaurus* [123] and potentially *Giraffatitan* [119] (L.M.W. and R.C.R., unpublished data) being the only exceptions. Their presence in *Sarmientosaurus*, subtle though it may be, thus carries some significance.

Ventrally, a large pituitary fossa is preserved, and is similar in size to that of other sauropods. The infundibular region connecting the pituitary to the remainder of the endocranial cavity is relatively simple in that it lacks the peculiar caudal expansion observed in the Uzbekistan titanosaur [63]. *Sarmientosaurus* also lacks the median canal (which is presumably venous in origin, a portion of the ventral longitudinal sinus [63]) connecting the infundibular region to the ventral part of the brainstem. This median canal often escapes notice, and has a patchy distribution in that it is present in *Spinophorosaurus* [117], *Camarasaurus* [118], and *Giraffatitan* (L.M.W. and R.C.R., unpublished data), as well as the derived titanosaurs *Bonatitan* [21] and *Jainosaurus* (L.M.W. and R.C.R., unpublished data) and the unnamed titanosaur from Uzbekistan [63]. Thus, its absence in *Sarmientosaurus* would appear to be an apomorphy. The pituitary fossa of the new Patagonian taxon resembles that of most sauropods in lacking a ventral median canal answering to the craniopharyngeal canal, whereas such a canal is present in the Uzbekistan titanosaur [63].

#### Cranial nerves and vasculature

The cranial nerves (CN) and vascular canals are clearly visible and typical of many sauropods [118,134] (Fig 10). As with most fossil sauropsid endocasts, traces of the olfactory nerves (CN I) themselves are not preserved. The optic nerve (CN II) enters the endocast just dorsal to the infundibular region of the pituitary. The oculomotor (CN III) and trochlear (CN IV) nerves exit through separate foramina in the infundibular region at the juncture of the orbitosphenoid and laterosphenoid, ventral to the foramen for the orbitocerebral vein. Earlier works [131,135–137] had regarded the largest and most dorsal of the foramina located in the orbitosphenoid—laterosphenoid suture as transmitting the trochlear nerve, but Witmer et al. [118] reidentified this aperture as venous and named it the orbitocerebral vein foramen. The presence of all three foramina (oculomotor nerve, trochlear nerve, and orbitocerebral vein) on both sides of MDT-PV 2 lends further support to this hypothesis, given that all cranial nerves are accounted for in this specimen. The fact that the orbitocerebral vein opening leads to a known venous structure (the rostral dural expansion) confirms this identification. All three of these structures extend more laterally than rostrally, consistent with the trend of the orbits to migrate caudally in derived titanosaurians, presumably in connection with the caudal expansion of the narial region. The cerebral carotid arteries enter near the ventral limit of the pituitary fossa. A pair of sphenopalatine arteries exit halfway up the pituitary.

The trigeminal nerve (CN V) exits caudal to the infundibular region, ventral to the transverse sinus. Unlike any other described sauropod, the ophthalmic (CN V_1_), maxillary (CN V_2_), and mandibular (CN V_3_) branches exit the braincase through separate foramina. *Sarmientosaurus* is unique within Sauropoda in having separate foramina for any of these branches, let alone all three (Fig 10). Moreover, we are interpreting a clearly discernible structure that arises from the left ophthalmic nerve as the supraorbital nerve, a branch rarely seen in any dinosaur group, although we cannot rule out the hypothesis that it is instead a canal for the trigeminal vein and artery. The abducens nerve (CN VI) exits the endocast at the pontine flexure and passes lateral to the pituitary fossa without penetrating it, an apomorphic condition diagnostic of titanosaurs [19,21,63]. This character is likely related to the retraction of the orbits in titanosaurs such that they face directly laterally, as reflected in the lateral orientation of the oculomotor and trochlear nerve canals and the abducens canal taking a more lateral course. As in other sauropsids, the facial nerve (CN VII) exits the endocranial cavity in common with two branches of the vestibulocochlear nerve (CN VIII), extending ventrolaterally. The more dorsal branch of the vestibulocochlear nerve enters the vestibular labyrinth on the medial side, ventral to the ampulla for the rostral semicircular canal, and likely received input from the pars vestibuli of the inner ear. The more ventral branch enters the cochlear fossa. The vagal (= jugular, metotic) foramen exhibits the typical reptilian conformation, likely transmitting the glossopharyngeal (CN IX), vagus (CN X), and spinal accessory (CN XI) nerves. As it passes the inner ear, it accepts the perilymphatic foramen (fenestra cochleae) from the cochlear fossa. Caudally, there are two rootlets for the hypoglossal nerves (CN XII).

#### Inner ear

**Fig 11:**
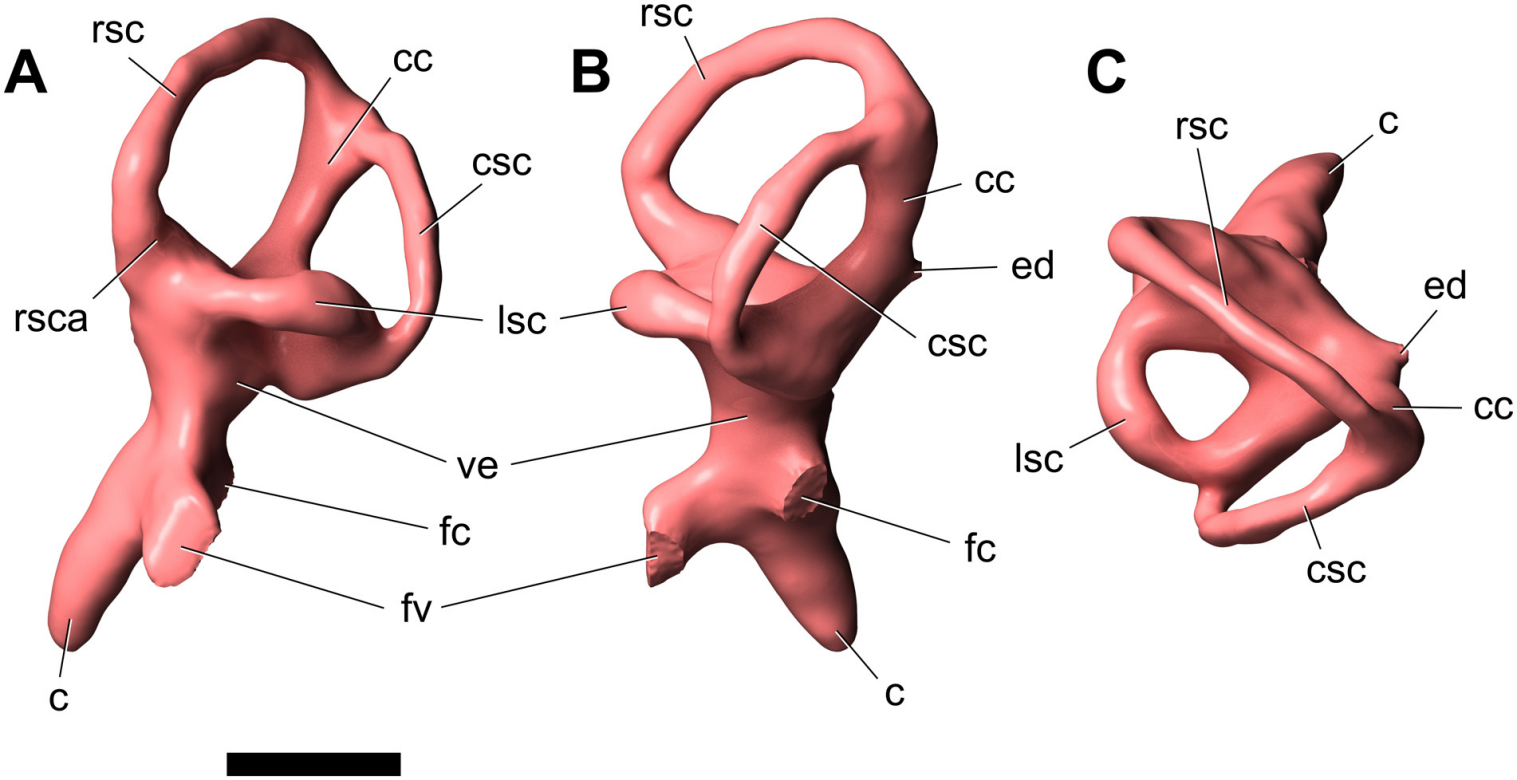
Left endosseous inner ear labyrinth of *Sarmientosaurus musacchioi* gen. et sp. nov. (MDT-PV 2). Computed tomography-based digital visualization in lateral (**A**), caudal (**B**), and dorsal (**C**) views. Abbreviations see text. Scale bar = 1 cm.

**Fig 12:**
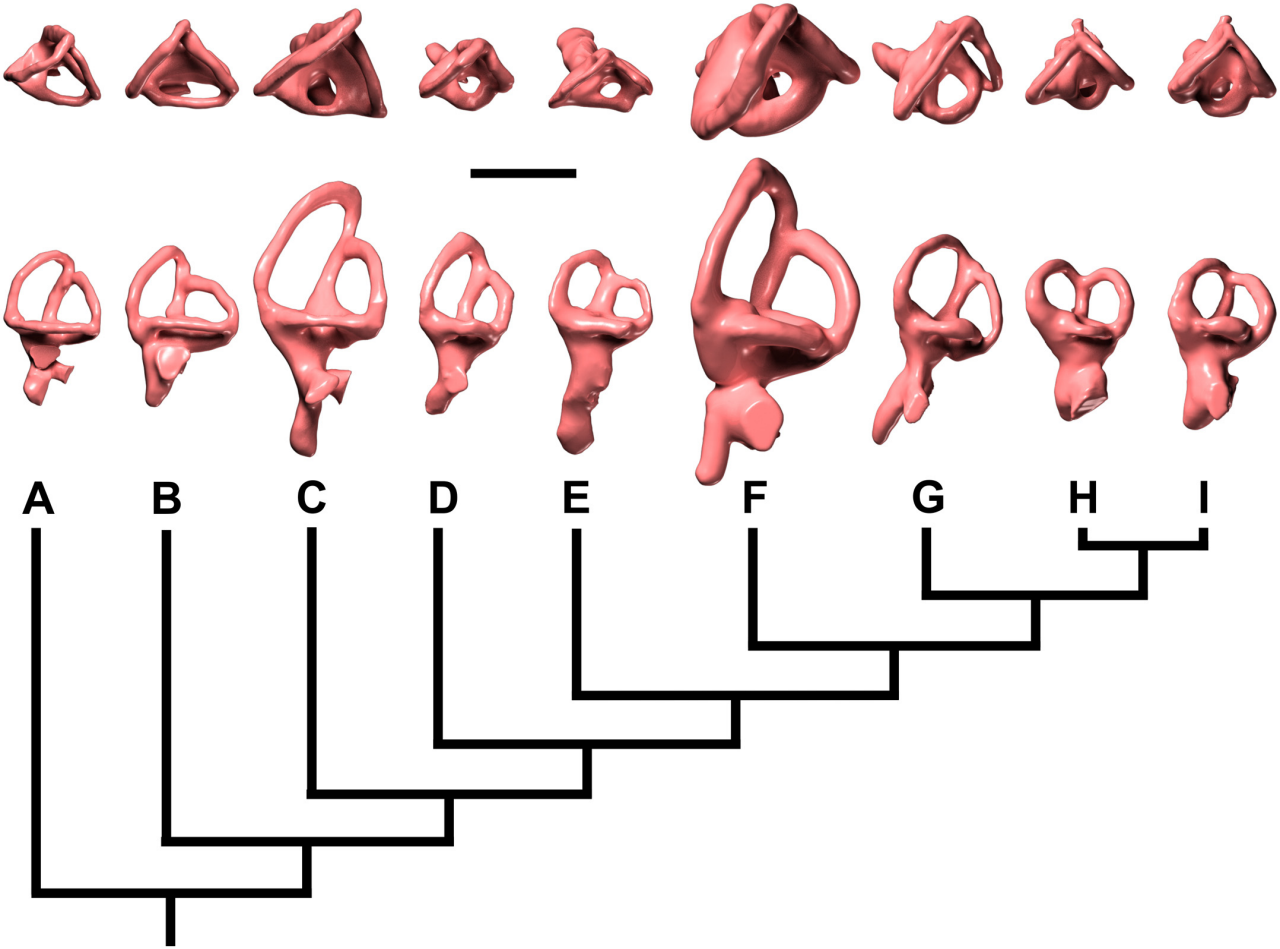
Comparison of left endosseous inner ear labyrinths of *Sarmientosaurus musacchioi* gen et sp. nov. (MDT-PV 2) and other saurischian dinosaurs in a phylogenetic context. Images are computed tomography-based digital visualizations in dorsal (top row) and lateral (bottom row) views. (**A**) The basal theropod *Herrerasaurus ischigualastensis* (MCZ 7063). (**B**) The basal sauropodomorph *Plateosaurus engelhardti* (MB R.1937). (**C**) The basal eusauropod *Spinophorosaurus nigerensis* (GCP-CV-4229). (**D**) The derived diplodocoid *Diplodocus longus* (CM 3452). (**E**) The basal macronarian *Camarasaurus lentus* (CM 11338). (**F**) The basal titanosauriform *Giraffatitan brancai* (MB R.2180.22.1–4). (**G**) The basal lithostrotian titanosaur *Sarmientosaurus musacchioi* (MDT-PV 2). (**H**) An unnamed derived lithostrotian titanosaur from Uzbekistan (CCMGE 628/12457). (**I**) The derived lithostrotian titanosaur *Jainosaurus septentrionalis* (ISI R162). A, C, D, E, F, I modified from Knoll et al. [117]; H modified from Sues et al. [63]. Scale bar = 2 cm.

The inner ear of *Sarmientosaurus* (Fig 11) displays features both primitive and intermediate among sauropod evolution on the line to derived Titanosauria. As a whole, the vestibular labyrinth is intermediate in size between the disparately large labyrinth of *Giraffatitan* and the proportionally much smaller sizes seen in more advanced titanosaurs (Fig 12). The semicircular canals are more slender than those of more basal taxa. In particular, the lateral canal is remarkably long and slender in comparison to other sauropods, which may indicate increased sensitivity in the mediolateral plane. Vertebrate semicircular canals sense angular acceleration and turning movements of the head in connection with neural mechanisms to coordinate eye, head, and neck movements for gaze stabilization (see [118,129,132] and references therein). Thus, the elongate lateral canal of *Sarmientosaurus* (Fig 12G) may reflect behaviors that emphasized lateral scanning movements of the eyes and head. This stands in contrast to the interepretation of the dramatically short lateral semicircular canals of other sauropods, especially other titanosaurs, as suggesting a behavioral emphasis on dorsoventral movments of the head, which is consistent with inferred feeding behaviors [118]. While still the longest of the three canals, the rostral canal is reduced in length relative to the others, more so than in *Giraffatitan*, but not to the extent seen in some other titanosaurs in which the rostral and caudal canals tend to be subequal in length. Interestingly, the angle between the rostral and caudal canals averages 63°, whereas these canals tend to be more orthogonal in bracketing taxa (Fig 12; see also [19,21]). As a 90° angle (i.e., orthogonality) in *Sarmientosaurus* would cause the rostral canal to breach the endocast, perhaps the reduced angle is an intermediate step in response to the shifting of the orbit caudolaterally and the concomitant compression of the temporal region. The smaller labyrinth of more derived titanosaurs is more orthogonal, and it is possible that the relaxed packaging constraints of its small size allowed a return to orthogonality.

**Fig 13:**
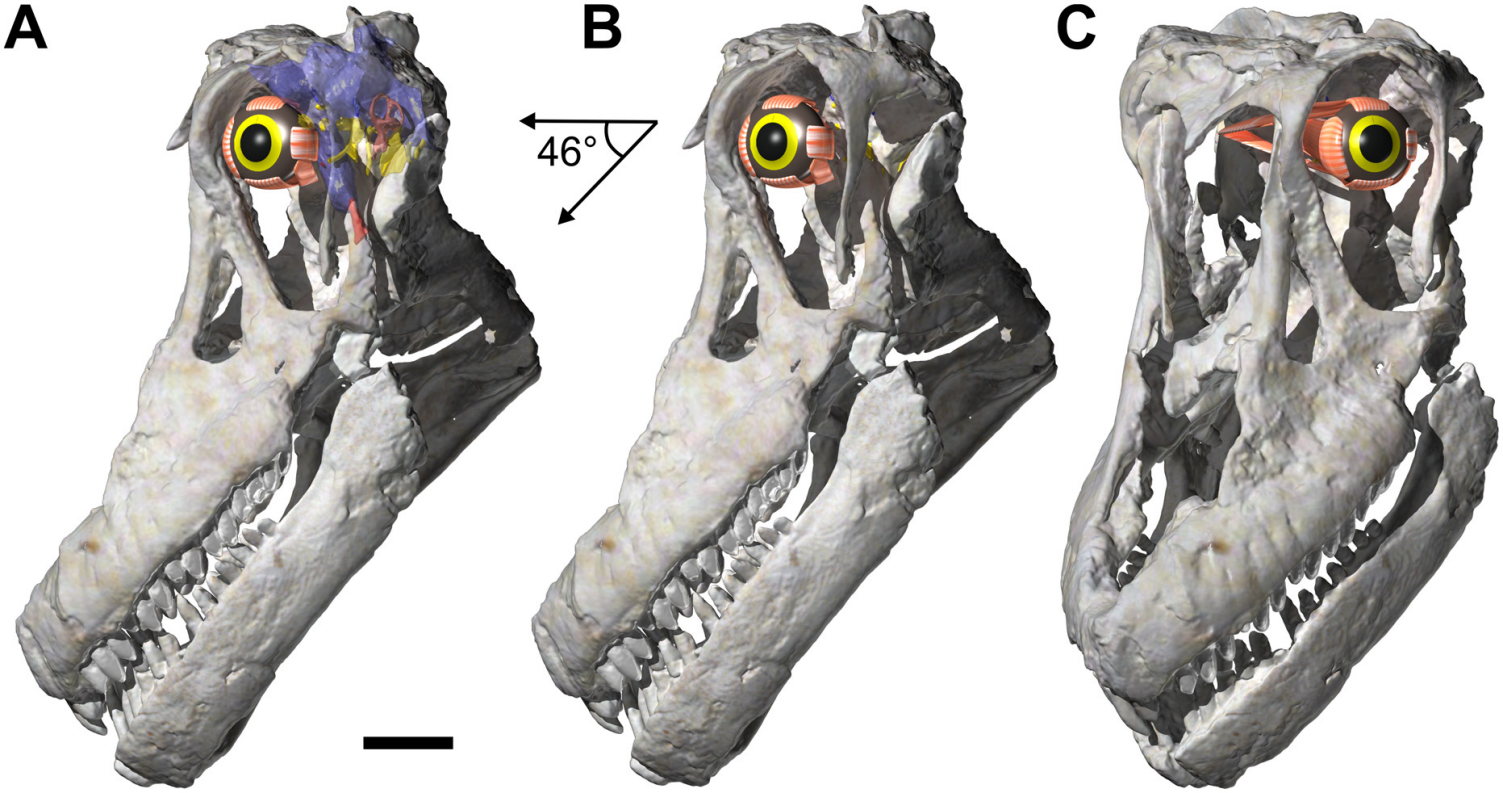
Computed tomography-based digital visualizations of the skull of *Sarmientosaurus musacchioi* gen. et sp. nov. (MDT-PV 2) in the ‘alert’ posture. (**A**) Skull rendered semitransparent in left lateral view, revealing the cranial endocast. (**B**) Skull rendered solid in left lateral view. (**C**) Skull rendered solid in left rostrolateral view. With the lateral semicircular canals oriented roughly with Earth horizontal, the skull is oriented with the tooth row 46° below horizontal. The eyeball is restored to provide a sense of the impact of the the strongly downturned ‘alert’ head posture on the visual field. Although the bony scleral ring was not preserved in *Sarmientosaurus*, its dimensions were based on scaling the complete ring of the titanosaur *Nemegtosaurus mongoliensis* (ZPAL MgD-I/9) and using known osteological correlates preserved n MDT-PV 2. Scale bar = 5 cm.

The endolymphatic canal communicates with the endocranial cavity at the base of the common crus. The fenestra vestibuli (fenestra ovalis) and fenestra cochleae (fenestra pseudorotundum, perilymphaticum) are both displaced ventrally, as seen in other sauropods, leaving the possibility for a large utricular space relative to non-neosauropod outgroups. This finding suggests that sensing linear acceleration was important to these animals. The cochlea (= lagena of some other authors) is located ventral to the fenestra vestibuli and is angled more strongly rostrally than in most other sauropods. The cochlea is similar in relative length to those seen in *Spinophorosaurus*, *Camarasaurus*, and *Giraffatitan*, but much longer than the cochleae preserved in diplodocids and more advanced titanosaurs (Fig 12). Given that the cochlea houses the hearing organ (i.e., the neuroepithelium of the basilar papilla), the relatively elongate cochlea of *Sarmientosaurus* may suggest a greater reliance on airborne sounds and potentially lower frequency hearing than in other titanosaurs (see [118,129]). When the skull of *Sarmientosaurus* is aligned with the lateral canal to Earth horizontal in the alert posture [118,132], the skull becomes downturned roughly 46° relative to the tooth row, with the occipital condyle being nearly horizontal (Fig 13).

### Mandible

When the holotype of *Sarmientosaurus* was discovered, the two mandibular rami were articulated to the cranium and to each other at the symphysis. The somewhat eroded ventral margin of the mandible comprised most of the cranial bone that was visible in the outcrop (Fig 2A–2C).

**Fig 14:**
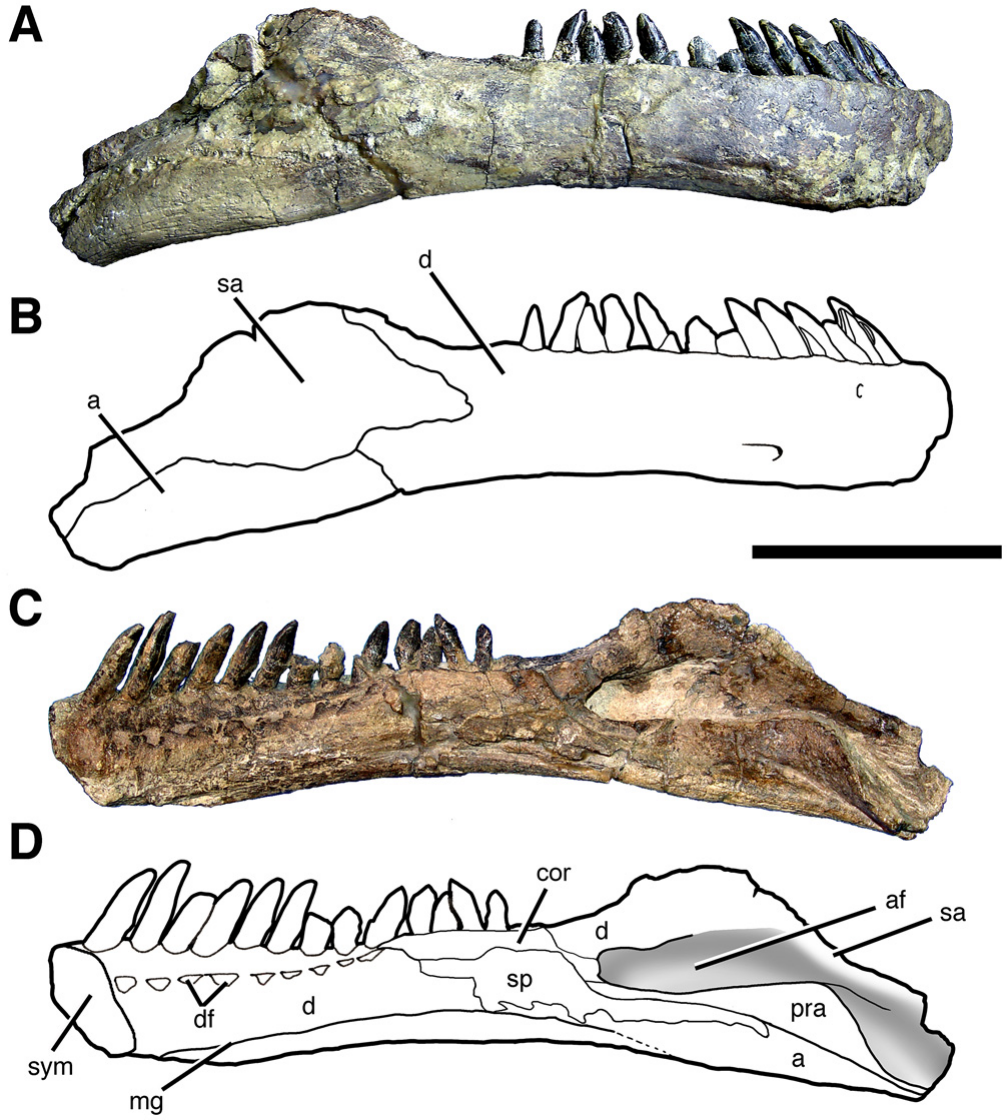
Right mandibular ramus of *Sarmientosaurus musacchioi* gen. et sp. nov. (MDT-PV 2). Photographs (**A**, **C**) and interpretive drawings (**B**, **D**) in lateral (**A**, **B**) and medial (**C**, **D**) views. Abbreviations see text. Scale bar = 10 cm.

**Fig 15:**
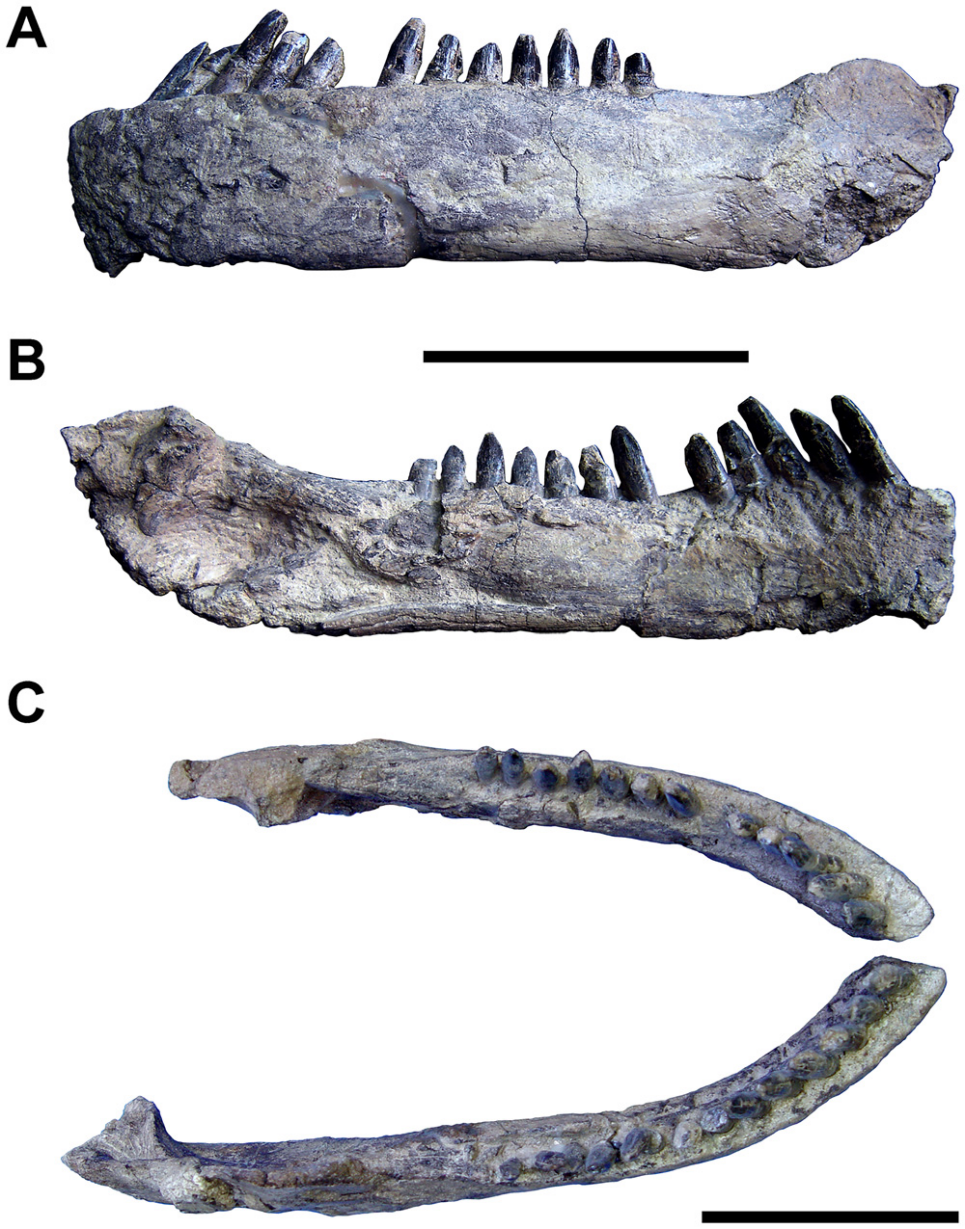
Mandible of *Sarmientosaurus musacchioi* gen. et sp. nov. (MDT-PV 2). Partial left mandibular ramus in lateral (**A**) and medial (**B**) views. (**C**) Articulated mandible in dorsal view. Scale bars = 10 cm.

**Fig 16:**
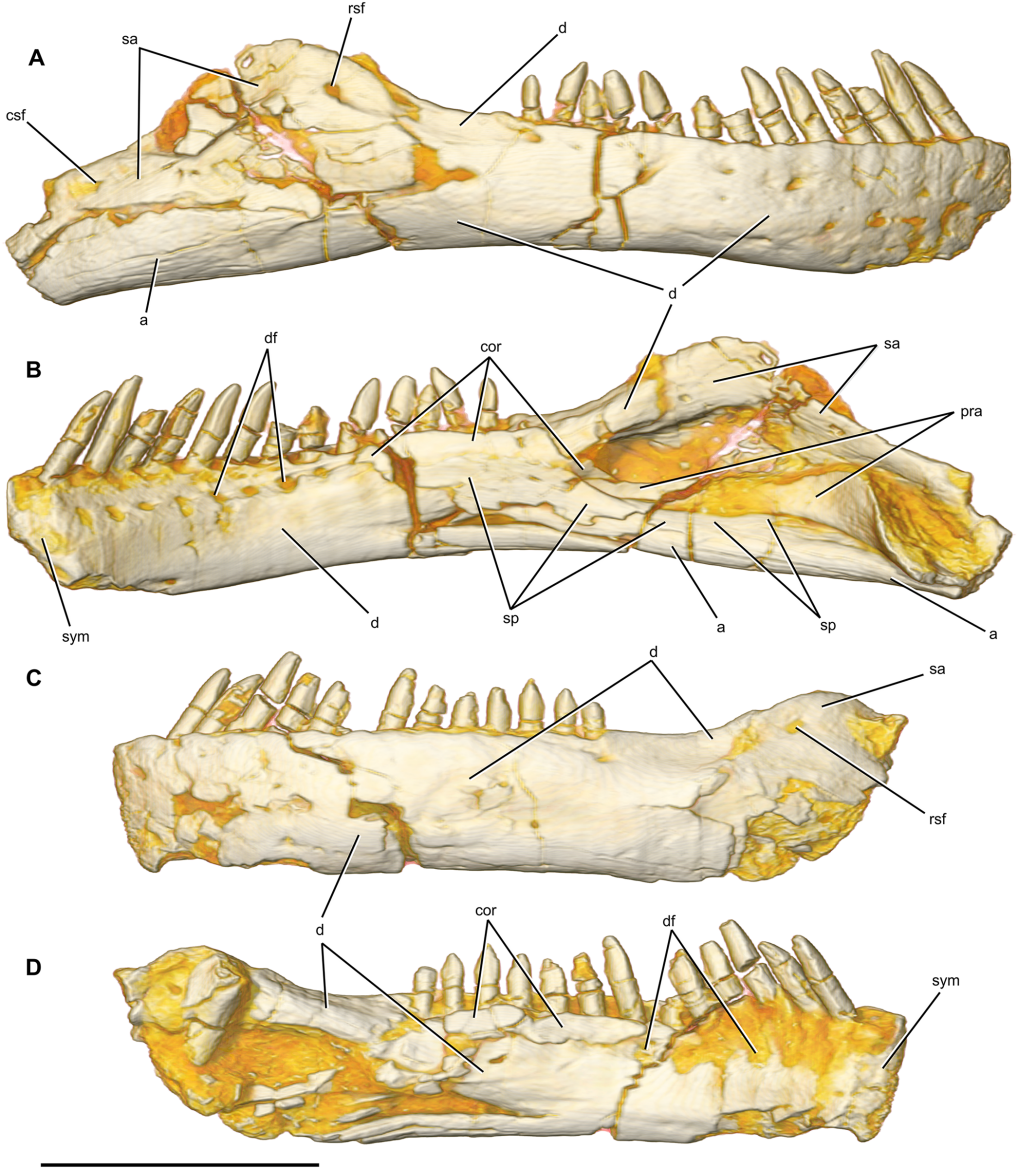
Mandibular rami of *Sarmientosaurus musacchioi* gen. et sp. nov. (MDT-PV 2). Computed tomography-based digital visualizations of right and left mandibular rami in lateral (**A, C**) and medial (**B, D**) views, respectively, indicating component bones. Abbreviations see text. Scale bar = 10 cm.

The right mandibular ramus (Figs 14–16; S1 Fig; S6, S7 and S8 Movies) is nearly complete, preserved from the symphysis to the jaw joint, whereas the left (Figs 15 and 16; S1 Fig; S6, S7 and S8 Movies) is damaged, and the area caudal to the coronoid eminence is missing. The mandibular rami are relatively elongate and articulate rostrally at the symphysis, and they lack external mandibular fenestrae. External mandibular fenestrae have, however, been reported in the Auca Mahuevo titanosaur embryos [15–17] and in the skull of an undescribed titanosaur from the Upper Cretaceous of Rincón de los Sauces in northern Patagonia [138]. This fenestra also appears to be present in *Tapuiasaurus* (see Zaher et al. [14]:fig. 1). In *Sarmientosaurus*, the lateral surfaces of the mandibular rami are reasonably well preserved, although the surangular, angular, and symphyseal region are all damaged in the right ramus, and the latter area is slightly eroded in the left ramus as well. Conversely, significant parts of the medial surfaces of both rami are fractured, cracked, and pitted. As a result, only the Meckelian groove and the suture between the angular and prearticular are clear when externally examining the original fossil. Our identifications of sutures between the other elements exposed on the medial side of the mandible are tentative and based largely on CT data.

The ‘U-shaped’ dorsal profile of the articulated mandible of *Sarmientosaurus* is shared with many sauropods. Conversely, in many diplodocoids and the titanosaurs *Bonitasaura* [23,24], *Brasilotitan* [25], and *Antarctosaurus* [9], the mandible is rectangular, or nearly so, in dorsal view; among titanosaurs, this condition is particularly marked in the latter two genera. Each mandibular ramus of *Sarmientosaurus* preserves a total of six bones: the dentary, surangular, and angular, which are exposed both laterally and medially; and the splenial, prearticular, and coronoid, which are visible only in medial aspect. Furthermore, most of the right articular appears to be present but fused to the prearticular. The large adductor fossa is prominent in medial and caudal views.

The mandible of *Sarmientosaurus* has two distinct and well-differentiated regions. The rostral region extends from the symphysis to the caudal end of the tooth row, and maintains a near-constant dorsoventral height throughout its length. This contrasts the dorsoventral symphyseal expansion of *Antarctosaurus*, *Bonitasaura*, *Brasilotitan*, *Malawisaurus*, *Nemegtosaurus*, *Rapetosaurus*, and *Tapuiasaurus* and the slight rostral increase in height in *Europasaurus* and *Giraffatitan*, and is similar to the morphology present in *Abydosaurus*; the rostral end of the mandible of the Rincón de los Sauces taxon is, however, described as very dorsoventrally low [138]. The caudal region begins just caudal to the tooth row; here, the mandible progressively expands up to the rostral end of the coronoid eminence, where it reaches its greatest height. More caudally, it gradually narrows in dorsoventral dimension toward the articular. This narrowing is not as abrupt as that present in *Camarasaurus*, *Nemegtosaurus*, *Quaesitosaurus*, *Rapetosaurus*, *Tapuiasaurus*, and possibly *Rinconsaurus* (Calvo and González Riga [45]:pl. 1b), nor as gradual as that in *Euhelopus*, but is comparable to the condition in *Abydosaurus*, *Europasaurus*, and *Giraffatitan*. The dorsolateral margin of the dentary possesses a vertical lamina of bone that borders the tooth row, similar to but lower than the lamina of *Nemegtosaurus*. The dorsolateral and dorsomedial margins coalesce caudal to the tooth row, delineating a triangular dorsal concavity that points toward the adductor fossa. As observed in medial view, the mandibular symphysis is oriented almost perpendicular to the long axis of the mandible, as in *Abydosaurus*, *Brasilotitan* [25], *Nemegtosaurus*, and *Quaesitosaurus* [139]. In *Antarctosaurus* (see Huene [9]:pl. 29, fig. 5c) and *Malawisaurus* (see Jacobs et al. [102]:fig. 1b; Gomani [32]:fig. 7b), by contrast, the symphysis seems to be oriented more rostrodorsally—caudoventrally, presumably due to the ventral deflection (‘downturn’) of the rostral end of the dentary in these taxa. The symphysis of *Sarmientosaurus* is subrectangular in medial view, as in *Antarctosaurus* and *Rapetosaurus*, not medially convex (i.e., ‘D-shaped’ or ‘P-shaped’) as it is in *Bonitasaura* [24] and *Brasilotitan* [25]. The ventral edge of the mandibular ramus is markedly mediolaterally convex. The mandibular articulation with the cranium is at a level ventral to the tooth row, as in other titanosauriforms [140].

#### Dentary

The dentary (Figs 14–16; S1 Fig; S6, S7 and S8 Movies) is the largest bone of the mandible, and contacts the opposing dentary at the symphysis, the splenial and coronoid caudomedially, the surangular caudodorsally, and the angular caudoventrally. The dorsal and ventral edges of the dentary are subparallel. In *Rapetosaurus* and especially *Malawisaurus*, however, the dorsal margin of the dentary is straight but the ventral margin is concave in medial and lateral views. As noted above, the symphysis is subvertical and nearly perpendicular to the long axis of the mandibular ramus, and its contour is subrectangular. It is mediolaterally thick, more so than in *Nemegtosaurus* and the ventral half of the dentary of *Brasilotitan* (see Machado et al. [25]:fig. 2e). The caudal contact with the surangular is very broad and sinuous, and extends from the rostral base of the tall coronoid eminence to the contact with the angular along a rostrally convex path. The caudoventral contact with the angular is short and caudodorsally oriented. There is no indication of the ‘accessory process’ of the dentary caudoventral process that occurs in *Abydosaurus* and *Giraffatitan* [98]. On the medial surface, the Meckelian groove extends along the ventral edge of the dentary, with part of its caudal portion bordering the splenial. It is not possible to verify whether the groove reaches the symphysis as in *Nemegtosaurus*, but in its caudal course along the dentary it rises and curves gently. The suture of the dentary with the splenial is best viewed on the left mandibular ramus, where the latter bone is mostly missing. The lateral surface of the dentary exhibits vascular foramina that are smaller than those in the maxilla and less profuse than those in *Nemegtosaurus*. Nine dental foramina, which correspond to the first nine tooth positions, are visible on the medial surface of the right dentary, adjacent to the dorsal edge of the bone. The coronoid bone covers the dental foramina medially, at least as far rostrally as the seventh dentary tooth, as seen on the left side. The dental foramina rostral to the coronoid are damaged in the left dentary.

Each dentary contains 13 tooth positions, with the largest teeth being closest to the symphysis as in *Ampelosaurus atacis* (see Le Loeuff [18]:fig. 4.3c), *Bonitasaura*, *Camarasaurus*, *Giraffatitan*, *Nemegtosaurus*, and *Rapetosaurus*. The tooth row begins immediately caudal to the symphysis, and the portion of the dentary caudal to the tooth row does not display a sharp ridge (termed the ‘guillotine crest’ by Apesteguía [23] and the ‘posterior crest’ by Gallina and Apesteguía [24]) as in *Bonitasaura*, *Brasilotitan*, probably *Antarctosaurus* and *Rapetosaurus* [25], and possibly the little-known African titanosaur *Karongasaurus* (see Gomani [32]:27). In dorsal view, the dental series curves gently rostromedially, as in *Euhelopus*, but not as much as in *Bonitasaura*, *Karongasaurus*, *Nemegtosaurus* (see Nowiński [10]:pl. 14, fig. 1a), *Rapetosaurus*, and especially *Antarctosaurus* and *Brasilotitan*. This contrasts the straighter dorsal profile of *Camarasaurus*.

#### Surangular

The surangular (Figs 14–16; S1 Fig; S6, S7 and S8 Movies) is much more completely preserved in the right mandibular ramus, although its contact with the dentary is better preserved on the left side. Its lateral aspect contacts the dentary rostrally and the angular ventrally. Its dorsal surface is slightly damaged on the right side but the remainder is well preserved. The central sector of the surangular is slightly laterally convex. The bone is very mediolaterally narrow in this region, which corresponds to the presence of the adductor fossa on its medial side. This fossa is filled with sediment but CT images reveal that it is rectangular in section with straight borders. The coronoid eminence is made up entirely by the surangular. The rostral surangular foramen is well preserved and present on both sides, with its proportional size and position most closely resembling the conditions in *Camarasaurus* [96,103] and *Tapuiasaurus* [14]. In *Abydosaurus* [98] and *Giraffatitan* [103], by contrast, the rostral surangular foramen appears to be placed slightly more dorsally, and in *Nemegtosaurus* [11] and *Rapetosaurus* [11,13] it is larger and more caudally situated. The caudal surangular foramen of *Sarmientosaurus* is preserved on the right side, in a caudal position similar to that in most macronarians (e.g., *Camarasaurus*, *Giraffatitan*, *Nemegtosaurus*), with the exception of *Tapuiasaurus* in which it is more rostrally placed. The medial surface of the surangular contacts the dentary rostrodorsally. Here, most of the surangular is occupied by the large adductor fossa, which housed mandibular adductor musculature and is shaped like a rostrocaudally elongate ellipse.

#### Angular

The angular (Figs 14–16; S1 Fig; S6, S7 and S8 Movies) is exposed laterally and medially. Laterally, its rostral margin contacts the dentary via a straight, caudodorsally-angled suture, whereas its dorsal edge meets the surangular along a suture that is slightly dorsally convex in lateral view. As in the surangular, the lateral surface of the angular is slightly convex and marked by fine longitudinal grooves. The lateral exposure of the angular is more dorsoventrally extensive than in *Camarasaurus*, *Nemegtosaurus*, *Quaesitosaurus*, and especially *Giraffatitan* and *Rapetosaurus*, more closely resembling *Abydosaurus* and *Tapuiasaurus* in this regard. Unlike in this latter Brazilian titanosaur, the caudal half of the angular is not turned markedly ventrally (see Zaher et al. [14]:fig. 1a, b). Medially, the dorsal portion of the angular is sheathed by the splenial, such that, in medial view, the angular and prearticular are separated by the splenial throughout much of their lengths. The angular participates in the adductor fossa, especially internally, as revealed by the CT data, but it is excluded from the medial border of this fossa by the splenial.

#### Splenial

The splenial (Figs 14–16; S1 Fig; S6, S7 and S8 Movies) is centrally located on the medial side of the mandible. As noted above, it is largely absent on the left mandibular ramus, and thus its description here is drawn from the right side. The splenial articulates with the dentary, coronoid, prearticular, and angular. Most of the edges of the bone are poorly defined and difficult to discern via gross external examination, but the CT data do allow its margins to be identified. The splenial contacts the dentary rostrally via an indistinct, generally dorsoventrally-arrayed suture. The dorsal contact of the splenial with the coronoid is also not clearly visible grossly, but it can be traced in the CT data. The splenial covers the rostral portion of the adductor fossa medially and overlaps the ventral half of the coronoid. The visible contact of the splenial with the prearticular is a nearly straight suture that is directed caudoventrally from the ventromedial border of the adductor fossa. The suture with the angular is also largely straight, and it is oriented nearly horizontally. The splenial covers the dorsal portion of the angular medially ventral to the adductor fossa and extends far caudally as a pointed splint between the angular and prearticular. The caudal termination of the Meckelian groove is in the ventral area of the splenial. The putative splenial of *Malawisaurus* exhibits some resemblance to that of *Sarmientosaurus* (see Gomani [32]:fig. 7d, e).

#### Prearticular—articular

As revealed by CT data obtained from the right mandibular ramus, the prearticular and articular appear to be coossified (Figs 14–16; S1 Fig; S6, S7 and S8 Movies). The portion of this compound element that is comprised by the prearticular is laminar and subvertically oriented, with a wide ventral base and a dorsal convexity, the caudal end of which is slightly sigmoid. The prearticular borders the coronoid rostrodorsally, the splenial rostroventrally, and the angular caudoventrally. The medial surface of the prearticular is smooth, whereas the lateral surface is obscured by sediment. The caudal contact of this bone with the angular is the best preserved of all mandibular sutures in *Sarmientosaurus*, and extends obliquely caudoventrally. The dorsomedial surface of the fused articular is rugose, as in *Camarasaurus* [96] and *Giraffatitan* [95]. Although it is incomplete caudally, enough remains of the articular of *Sarmientosaurus* to suggest that it was rostrocaudally longer than that of *Camarasaurus* but shorter than that of *Giraffatitan*.

#### Coronoid

*Sarmientosaurus* possesses a rostrocaudally elongate, dorsoventrally narrow, strap-like bone at the dorsal edge of the medial surface of the mandibular ramus, medial to the distalmost 6–7 teeth (Figs 14–16; S1 Fig; S6, S7 and S8 Movies). Madsen et al. [96] regarded this element as the intercoronoid, but we follow Wilson [11] in considering it to be the coronoid (see Wilson [11] for a discussion of the homology and nomenclature of this element). The coronoid of *Sarmientosaurus* is a mediolaterally thin bone that sheaths the alveolar region of the dentary dorsally (including the distal dental foramina); as preserved, it extends as far rostrally as the ninth dentary tooth on the right side and the seventh tooth on the left side. The ventral portion of the coronoid is itself obscured by the splenial. Caudally, the coronoid extends ventrally along the rostral margin of the adductor fossa to make a slight contact with the rostrodorsal tip of the prearticular. As might be expected in a basal lithostrotian, the coronoid of *Sarmientosaurus* is rostrocaudally shorter than those of *Camarasaurus* and *Giraffatitan* but longer than that of *Nemegtosaurus* (provided that the rostral terminus of the coronoid of the latter has been correctly identified [11]).

### Dentition

The skull of *Sarmientosaurus* was discovered with the jaws closed and the premaxillary and maxillary teeth overlapping those of the dentary. After the jaws were disarticulated, it became clear that the premaxillary teeth are subvertical, the maxillary teeth are procumbent (especially distally), and the dentary teeth are recumbent. Because there is no evidence that this unique condition is due to taphonomic distortion, we regard it as an autapomorphy of the new Patagonian titanosaur. Collectively, the preserved teeth and few empty alveoli indicate the following number of tooth positions: four in each premaxilla, 12 in the left maxilla and 11 in the right, and 13 in each dentary (see Table 2). All known sauropods have four premaxillary teeth; however, the number of maxillary and dentary tooth positions in *Sarmientosaurus* is greater than in some other titanosaurs. For example, the undescribed skull from Rincón de los Sauces has only seven or eight maxillary teeth [138], and *Rapetosaurus* and *Ampelosaurus atacis* preserve 11 and nine dentary alveoli, respectively [13,18], though the only known dentary of the latter taxon appears to be rostromedially incomplete. The maxillary and dentary tooth counts of the Auca Mahuevo embryos—estimated at 7–8 and ten, respectively—also appear to be lower than those of *Sarmientosaurus* [17]. Conversely, the dentary teeth of *Antarctosaurus* (15 or 16 [7,24]), *Brasilotitan* (14 [25]), and *Malawisaurus* (at least 15 [32,102]) are more numerous than in the new Patagonian taxon. Within Titanosauria, the dental formula of *Sarmientosaurus* appears closest to those of *Nemegtosaurus* and *Quaesitosaurus*, which have four premaxillary, eight or nine maxillary, and 13 dentary teeth [10,11,43], though judging from Zaher et al. ([14]:fig. 1) that of *Tapuiasaurus* may be even more similar. The dental formula of *Sarmientosaurus* is also very close to those of the titanosauriforms *Euhelopus* and *Abydosaurus*, which have four premaxillary, ten maxillary, and 13 and 14 dentary teeth, respectively [98,111].

**Table 2:** Number of teeth in MDT-PV 2, the holotype of *Sarmientosaurus musacchioi* gen. et sp. nov. Abbreviations: L, left; R, right.

Total	57
Premaxilla	4L, 4R
Maxilla	12L, 11R
Dentary	13L, 13R

**Fig 17:**
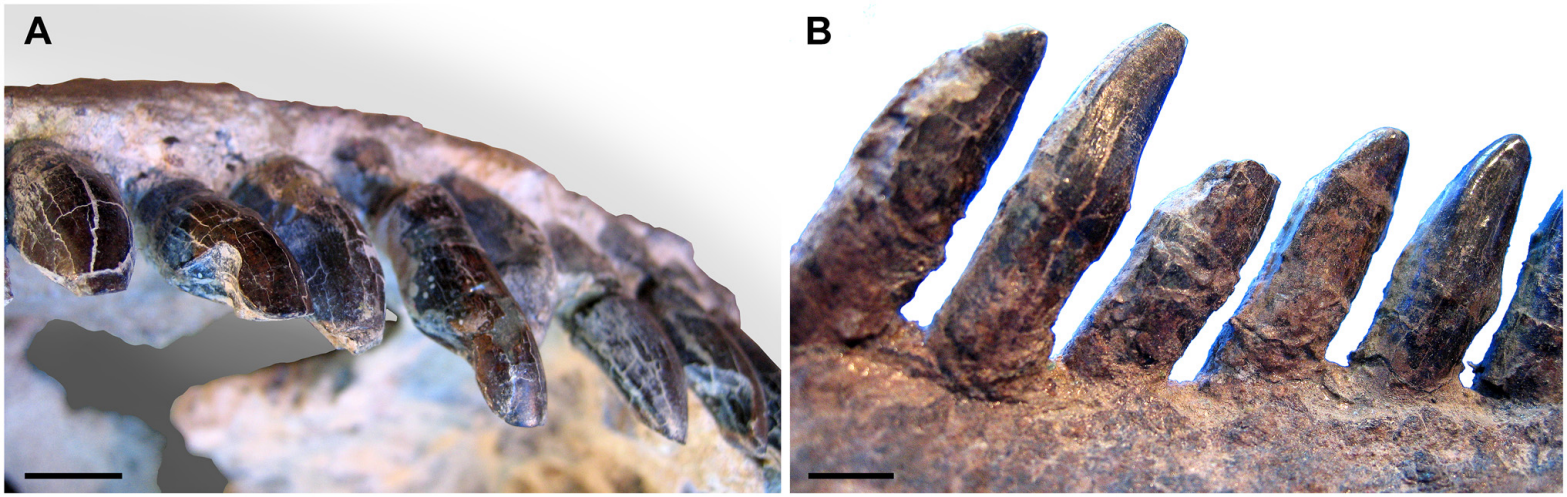
Tooth crowns of *Sarmientosaurus musacchioi* gen. et sp. nov. (MDT-PV 2). (**A**) Mesial right upper teeth (from left to right, fourth through first maxillary teeth and fourth through second premaxillary teeth) in apical and distal views, showing distal wear facets. (**B**) First through fifth right dentary teeth in lingual view. Scale bars = 1 cm.

The premaxillary teeth of *Sarmientosaurus* are larger than those of the maxilla and dentary (Table 3). In contrast to the condition in some titanosaurs (e.g., *Antarctosaurus*, *Brasilotitan*, *Dreadnoughtus* [26], *Petrobrasaurus* [141]), all tooth crowns are elliptical rather than subcircular or oval in cross section. They are compressed cone-chisel-shaped (*sensu* Calvo [140]) with high-angled wear facets, as in many titanosaurs; their Slenderness Index (*sensu* Upchurch [142]) is intermediate between those of the spatulate teeth typical of many sauropods and those of the elongate, pencil-shaped teeth characteristic of diplodocoids and multiple titanosaurs (e.g., *Antarctosaurus*, *Bonitasaura*, *Brasilotitan*, *Dreadnoughtus*, *Karongasaurus*, *Lirainosaurus*, *Maxakalisaurus*, *Nemegtosaurus*, *Petrobrasaurus*, *Pitekunsaurus*, *Quaesitosaurus*, *Rapetosaurus*, *Rinconsaurus*, *Tapuiasaurus*). The crowns do not overlap one another as they do in *Camarasaurus* [140]. As in *Giraffatitan* [143], the upper (i.e., premaxillary and maxillary) teeth exhibit much greater wear than the dentary teeth (Fig 17).

**Table 3:** Measurements (mm) of the teeth of MDT-PV 2, the holotype of *Sarmientosaurus musacchioi* gen. et sp. nov. Abbreviations: L, left; R, right.

		Premaxillary				Maxillary											
Upper teeth	L or R?	1	2	3	4	5	6	7	8	9	10	11	12	13	14	15	16
Apicobasal length, crown	L	---	---	---	40	34	27	41	29	---	24	24	25	---	---	---	22
	R	---	41	40	32	34	37	40	38	---	---	---	---	---	---	31	N/A
Mesiodistal width	L	---	---	---	13	11	11	12	12	11	11	10	10	12	12	12	11
	R	11	12	13	13	10	12	16	12	---	16	10	11	---	11	11	N/A
Labiolingual width	L	12	---	11	12	9	8	10	11	10	10	10	10	---	---	---	9
	R	10	10	10	10	9	10	11	10	---	---	---	---	---	---	10	N/A
Lower (dentary) teeth	L or R?	1	2	3	4	5	6	7	8	9	10	11	12	13			
Apicobasal length, crown	L	40	33	37	---	---	---	29	---	24	23	26	26	21			
	R	41	38	---	37	32	35	---	---	---	23	---	21	18			
Mesiodistal width	L	11	10	12	12	11	---	10	8	10	9	8	8	7			
	R	11	13	14	10	9	10	---	---	10	9	7	7	6			
Labiolingual width	L	9	9	9	9	9	---	7	7	6	7	6	5	6			
	R	9	9	9	10	8	9	---	---	7	8	7	7	6			

**Fig 18:**
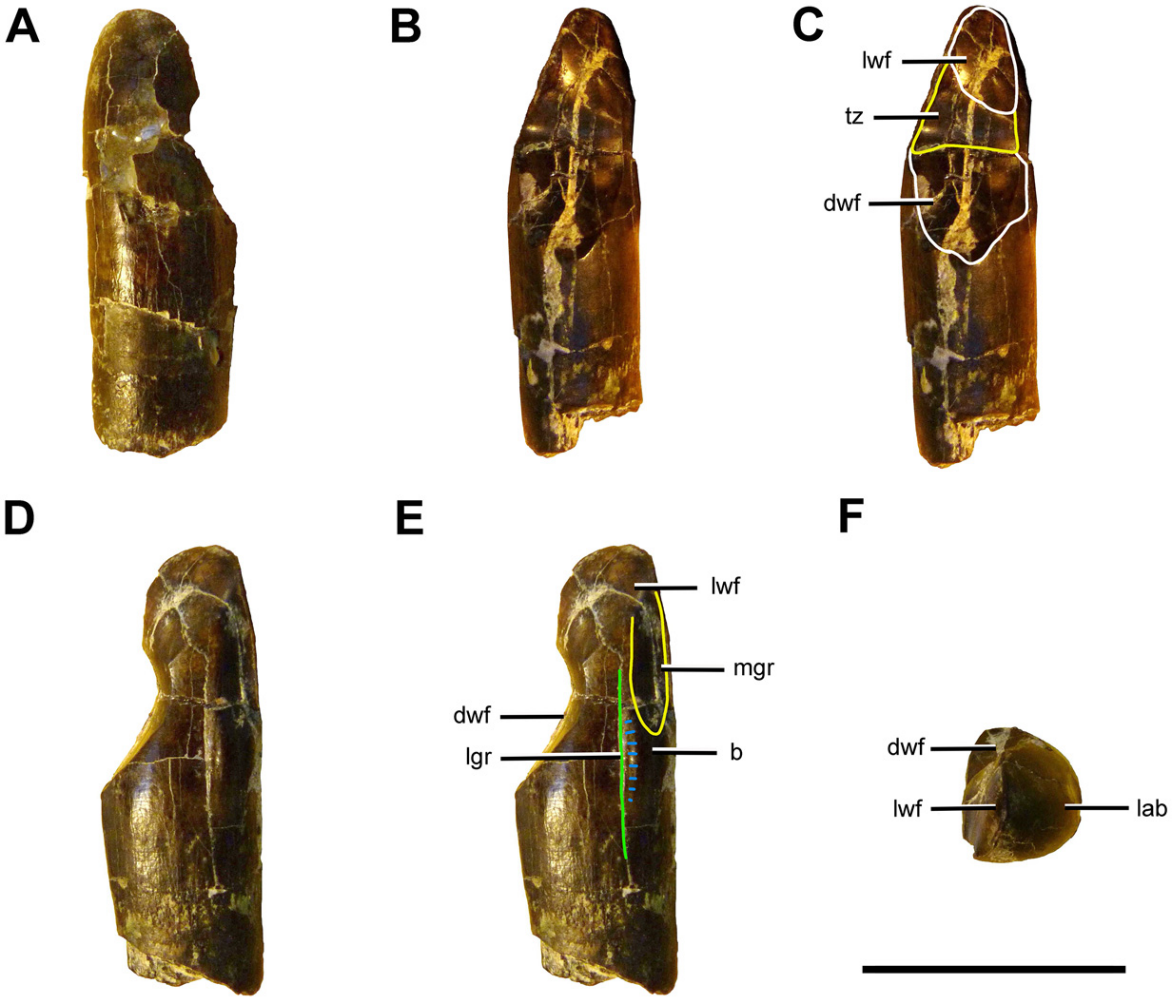
First right maxillary tooth of *Sarmientosaurus musacchioi* gen. et sp. nov. (MDT-PV 2). (**A**) Labial view. (**B**, **C**) Lingual view. (**D**, **E**) Mesiolingual view. (**F**) Apical view. Abbreviations see text. In C, the distal and lingual wear facets are outlined in white and the transitional zone is outlined in yellow. In E, the mesial groove is outlined in yellow, the large groove is indicated by a green line, and the bulge is indicated by short blue lines. Scale bar = 2 cm.

We arbitrarily choose the first right maxillary tooth (Fig 18) to represent the morphology of the upper teeth of *Sarmientosaurus*. In general terms, their functional morphology is as has been described for other titanosauriform teeth, such as USNM 187535 from the Lower Cretaceous of Texas [144] and MPCA-Pv 96 from the Campanian of Río Negro, Argentina [61]. The first right maxillary tooth has two wear facets situated at a high angle relative to the apicobasal axis of the tooth, one on the lingual surface and the other on the distal surface; the latter facet is essentially a continuation of the first. In this tooth, these facets are of subequal size, but in other *Sarmientosaurus* upper teeth the distal facet is sometimes larger than the lingual facet. In addition to these wear facets, there is a low, apicobasally-oriented crest or bulge on the mesiolingual face of the crown that is delimited by two slight grooves. The deeper and more distally positioned of these grooves extends along most of the length of the crown, whereas the shorter, shallower mesial groove reaches the apex of the lingual facet. García and Cerda [61] interpreted the origin of the lingual wear facet in the upper teeth of titanosaurs as a result of contact of the lingual surface with the labial face of the lower teeth. These authors regarded the distal facet as a hallmark of a tooth that had erupted improperly, at an angle to its ‘normal’ vertical axis, thereby contacting not only the labial or lingual faces of lower teeth (when the jaws were occluded) but also the mesial edge of the following upper tooth.

Some upper teeth of the *Sarmientosaurus* type specimen, such as the second left maxillary tooth and the fourth right premaxillary tooth, do not exhibit wear facets. As such, we infer that these teeth were not yet functional at the time of death of this individual. Certain areas of the *Sarmientosaurus* jaws alternately house fully functional teeth and younger, barely functional or not yet functional teeth (Figs 3–6, 8 and 13–16; S1 Fig; S1, S2, S3, S4, S6, S7 and S8 Movies). Unsurprisingly, their wear patterns vary with the position of the tooth and the degree to which it was used.

The basal halves of the lower (i.e., dentary) tooth crowns (Fig 17B) are subcylindrical in cross section. By contrast, the apical part of the lingual surface is gently excavated, rendering the crowns ‘D’-shaped in section here, as in *Malawisaurus*. This lingual concavity is present in all preserved dentary teeth, but it is best developed in the largest, most mesially-positioned teeth, where it occupies most of the apical half of the crown. The mesial and distal margins of each dentary tooth are subparallel throughout most of their extent but converge apically. The apex slopes gently lingually and is marked by a diminutive, semicircular wear facet. Whereas the enamel of the lingual concavity is smooth, that of the subcylindrical basal area shows fine, longitudinally-oriented wrinkles. The labial surface is gently convex toward the apex, and its enamel is mostly smooth. There is a fine groove in the apical sector of the mesial margin; this area of the distal margin is occupied by a more prominent wear facet with oblique borders.

**Fig 19:**
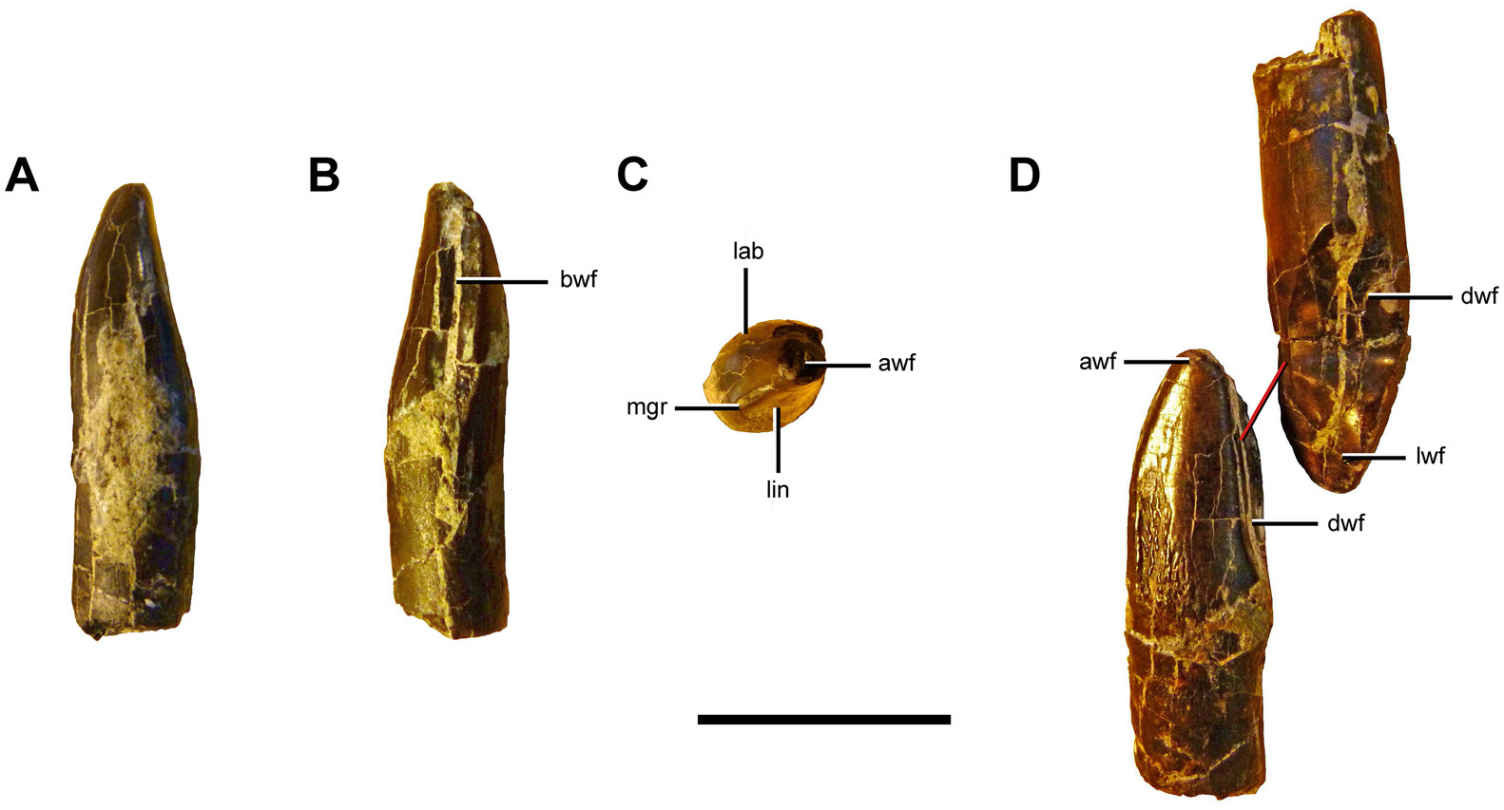
Tooth crowns of *Sarmientosaurus musacchioi* gen. et sp. nov. (MDT-PV 2). Fifth right dentary tooth in (**A**) mesiolabial, (**B**) distal, and (**C**) apical views. (**D**) First right maxillary tooth (top) and fifth right dentary tooth (bottom) showing their relative positions, with red line connecting their points of contact during occlusion. Abbreviations see text. Scale bar = 2 cm.

The general conformation of the lower teeth of *Sarmientosaurus* is reminiscent of, though slightly taller-crowned than, those of *Abydosaurus* [98]. A typical tooth of this morphology, the fifth right dentary tooth (Fig 19), has a lingual groove that borders the mesial surface and a well-developed distolingual wear facet. The lingual groove is in the concave part of the crown, and is lanceolate in shape with the point directed apically. It is arranged in two planes: an apical plane that is small and relatively flat with a pair of diminutive marks, and a basal plane that is rectangular and comparatively deep with several longitudinal rugosities. The distolingual facet is a long, deep groove that was produced by contact with the corresponding maxillary tooth (Fig 19D).

In sum, the upper teeth of *Sarmientosaurus* have two contiguous wear facets—lingual and distal—and grooves near the mesial margin, whereas the lower teeth are subcylindrical basally but lingually concave more apically, and possess a mesial or nearly mesial groove, a deep distal wear facet, and a smaller apical wear facet. This array of tooth morphologies forms a combination that has apparently never previously been documented within Macronaria.

A functional explanation for these features may be proposed following the criteria of García and Cerda [61], who emphasized the mutual and complex interrelationships between the upper and lower teeth at different growth stages. In the case of *Sarmientosaurus*, however, we must also consider the consequences of the fact that the opposing teeth are oriented differently: subvertical in the premaxilla and the rostral end of the maxilla, markedly procumbent in the caudal part of the maxilla, and recumbent in the dentary. Perhaps this complex dental arrangement explains the unique combination of wear facets observed in the upper and lower teeth of this new titanosaur. The presence of heavy wear on the upper teeth and only small apical facets on the dentary crowns demonstrates that, as in *Giraffatitan* [140,143], there was no tooth-to-tooth contact. Nevertheless, the distal wear facet of the lower crowns demonstrates the existence of the ‘interlocked’ or interdigitated dental contact proposed by Calvo [145] for the titanosauriform *Asiatosaurus* [146].

The Slenderness Index (SI), which is the ratio between the apicobasal height and the mesiodistal width of a given tooth crown [142], has been proposed to separate wide-crowned sauropods (e.g., *Camarasaurus*, *Euhelopus*, *Jobaria*) from their narrow-crowned counterparts (e.g., diplodocoids, many titanosaurs, e.g., *Nemegtosaurus*, *Rapetosaurus*). Upchurch [142] proposed that an SI of less than 3 corresponds to the wide-crowned group and an SI of approximately 4 or more to the narrow-crowned group, whereas Curry [147] regarded wide-crowned sauropods as those with an SI of less than 4 and narrow-crowned taxa as those with a value higher than 5. This index varies in *Sarmientosaurus*: of six complete, representative teeth in the right upper row, four have an SI of greater than 3 whereas the remaining two do not. We also calculated the SI of three teeth in the left upper row, which is more than 3 in each of these cases. Of the four right dentary teeth we examined in this fashion, all have SI values of 3 or more, and of four left dentary teeth, three have an SI of more than 3. These values suggest that the crowns of *Sarmientosaurus*, like those of the basal lithostrotian titanosaur *Malawisaurus* [32,102], record an intermediate stage between the spatulate teeth of basal macronarians such as *Camarasaurus* or relatively broad-crowned titanosaurs such as *Ampelosaurus atacis* [18,148] and the peg-like teeth of derived titanosaurians (e.g., *Antarctosaurus*, *Bonitasaura*, *Nemegtosaurus*, *Rapetosaurus*, *Tapuiasaurus*).

#### Tooth replacement

**Fig 20:**
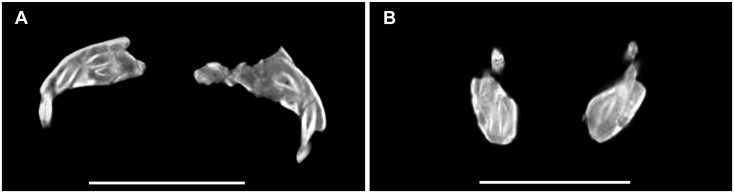
Tooth replacement in *Sarmientosaurus musacchioi* gen. et sp. nov. (MDT-PV 2) as revealed by computed tomography. (**A**) Transverse section through maxillae showing three replacement teeth in the right maxilla (on the left side of the image) and two in the left. (**B**) Transverse section through dentaries showing replacement teeth. Scale bars = 10 cm.

CT images of the premaxilla, maxilla, and dentary (Fig 20; S2, S3, S4, S6, S7 and S8 Movies) reveal that *Sarmientosaurus* has two replacement teeth per alveolus, except in the fourth right maxillary alveolus, which houses three germ teeth. Tooth replacement in the new taxon was of the ‘wave of alternating replacement’ type, as also described for *Nemegtosaurus* [10,11] and an indeterminate titanosaur dentary (MCSPv-061 [61]). As previously recognized in isolated titanosaurian dentigerous elements [55,61], during development, the germ teeth of *Sarmientosaurus* moved labially from their origin near the medial wall of the alveolus until they finally erupted.

#### Microwear

**Fig 21:**
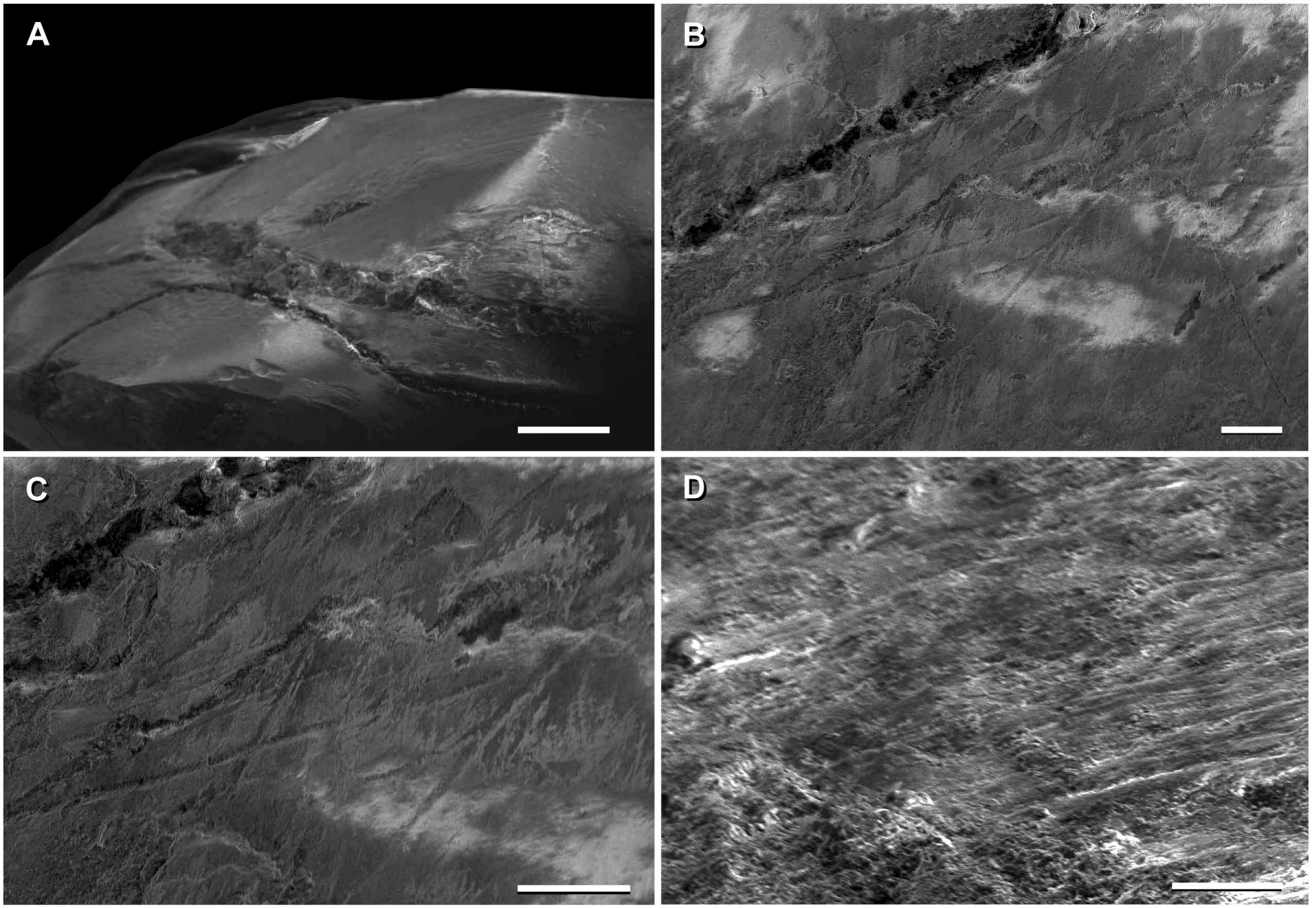
Scanning electron microscope images of first right maxillary tooth of *Sarmientosaurus musacchioi* gen. et sp. nov. (MDT-PV 2). (**A**) Lingual wear facet (enlarged 18 times). (**B**, **C**) Distal wear facet, showing microwear oriented generally parallel to the long axis of the tooth (enlarged 120 and 220 times, respectively). (**D**) Lingual wear facet, showing microwear oriented generally parallel to the long axis of the tooth (enlarged 430 times). Scale bars = 1 mm in A; 100 μm in B–C; 50 μm in D.

**Fig 22:**
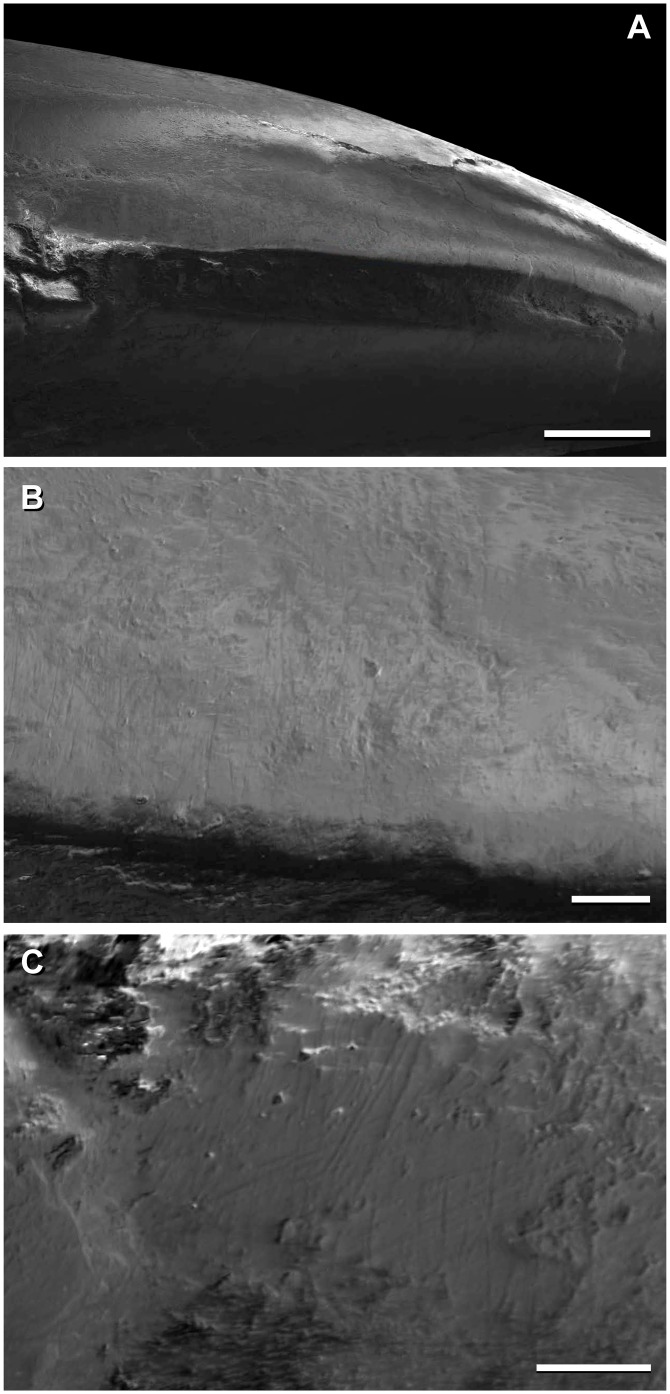
Scanning electron microscope images of mesial groove of fifth right dentary tooth of *Sarmientosaurus musacchioi* gen. et sp. nov. (MDT-PV 2). (**A**) Enlarged 20 times. (**B**) Enlarged 150 times. (**C**) Enlarged 220 times. Scale bars = 1 mm in A; 100 μm in B–C.

**Fig 23:**
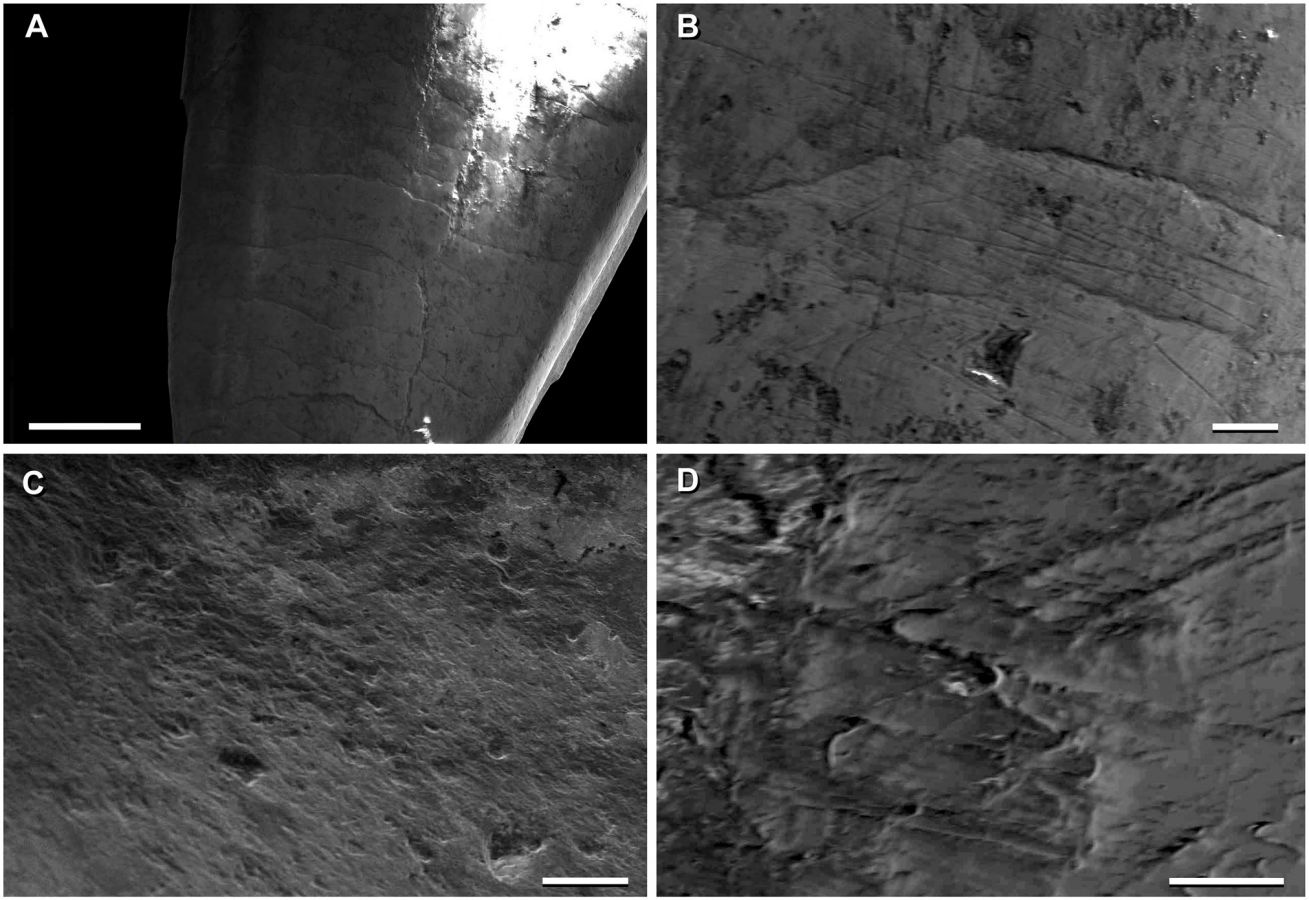
Scanning electron microscope images of fifth right dentary tooth of *Sarmientosaurus musacchioi* gen. et sp. nov. (MDT-PV 2). (**A**, **B**) Lingual surface (enlarged 22 and 130 times, respectively). (**C**) Apical facet (enlarged 85 times). (**D**) Labial zone (enlarged 450 times). Scale bars = 1 mm in A; 100 μm in B; 200 μm in C; 50 μm in D.

Numerous scanning electron microscope (SEM) images were taken of the first right maxillary and the fifth right dentary teeth of *Sarmientosaurus*, to examine their wear facets and to assess the new taxon for the grooves that have been recorded in the enamel of other sauropodomorphs [61,123,143,149]. SEM images of the maxillary tooth (Fig 21) show thick and thin grooves that sometimes intersect but that are generally oriented parallel to the long axis of the wear facet. The dentary tooth (Figs 22 and 23) shows a similar pattern in the area of the mesial groove and the labial surface, although the intersecting grooves may be slightly more dense than those in the maxillary tooth. The apical facet of the dentary tooth also exhibits densely-packed groups of pits of different depths and perimeters, as in specimens studied by García and Cerda [61]. The general orientation of the grooves suggests the existence of orthal jaw movement, as proposed by Calvo [140] for titanosauriforms.

An association between grooves and pits in the enamel and dentine and the diet of a given taxon has been proposed by multiple authors [123,149–151]. Nevertheless, other workers [61,152] have proposed that the origin of these traces may instead be related to the accidental ingestion of sediment associated with plant fodder, and therefore not strongly indicative of diet.

### Cervical Series

The type specimen of *Sarmientosaurus* preserves parts of the cranial and middle cervical series, including the partial axis and a piece of its right rib articulated to the cranial end of the third cervical vertebra, a fragment of the caudal end of the fifth cervical vertebra adhered to the sixth cervical vertebra and its articulated right rib, and the seventh cervical vertebra. The sixth and seventh cervical vertebrae are somewhat distorted and damaged by erosion; of these, the sixth is in marginally better condition and therefore provides more information. In the field, the right ventral side of the cervical series was exposed, whereas the left was missing or covered by sediment. The series was fully articulated to within a few centimeters of the skull, which was turned at an angle of nearly 90° relative to the craniocaudal axis of the neck. Although additional vertebrae were observed *in situ* and collected, only the aforementioned five could be recovered during laboratory preparation; the others were too damaged by weathering to be salvageable. In the right ventrolateral area of the neck, a cylindrical structure 3 mm in diameter originated a few centimeters from the occipital region of the skull and coursed along several vertebral centra (Fig 2). Situated ventrolateral to the preserved cervical ribs (i.e., above and lateral to the ribs in the field, since the cervical series was discovered with its ventral side up), this structure is regarded here as an ossified cervical tendon.

Our identification of the positions of the sixth and seventh cervical vertebrae is based on their location in the articulated cervical series as it appeared in the field. Both of these vertebrae may be regarded as middle cervicals, given that the few titanosaurs for which the cervical series is completely represented possess between 13 (the unidentified Brazilian titanosaur informally known as ‘Peirópolis Series A’ [7,153]) and 17 vertebrae (*Rapetosaurus* [154]) in this region of the skeleton.

#### Axis

**Fig 24:**
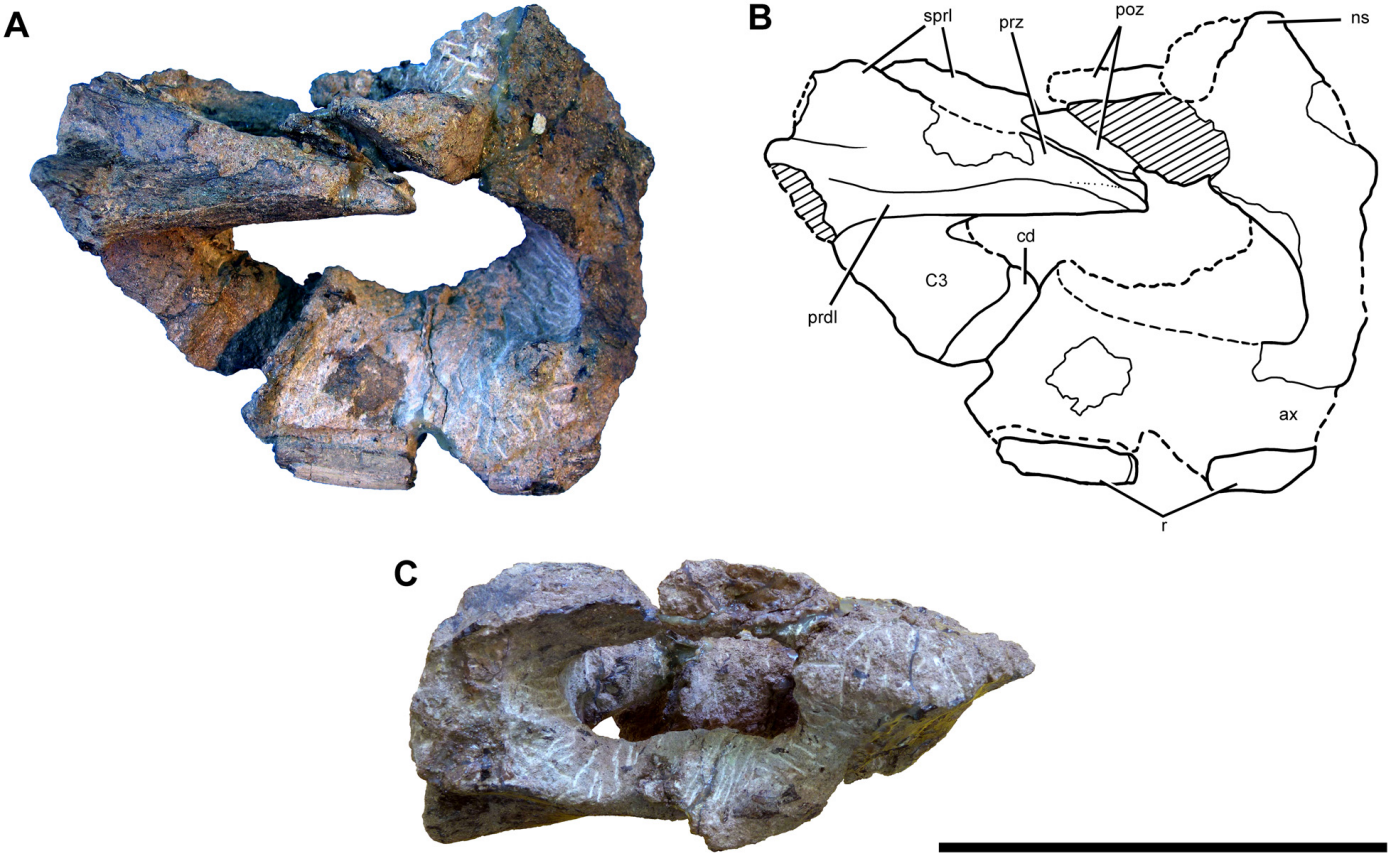
Articulated partial axis and cervical vertebra 3 of *Sarmientosaurus musacchioi* gen. et sp. nov. Photographs (**A**, **C**) and interpretive drawing (**B**) in right lateral (**A**, **B**) and dorsal (**C**) views. Abbreviations see text. Scale bar = 10 cm.

The caudal articular surface of the centrum of the axis (Fig 24) is markedly concave, a feature that can be observed because the cranial articular condyle of the third cervical vertebral centrum is partially eroded and displaced from its contact with the axial centrum. Although it is incomplete cranially, the axis appears to have been dorsoventrally low and craniocaudally elongate, as is the case in the other preserved cervical vertebrae of *Sarmientosaurus*. There is no evidence of lateral pneumatic fossae (‘pleurocoels’). A cervical rib fragment approximately 6 cm in total length is preserved as two pieces adhered to the right ventrolateral area of the centrum. The region of the centrum caudal to the ventral base of the neural arch is proportionally long. The preserved portion of the neural arch is subtriangular and wedge-shaped in lateral view. The neural spine is inclined caudodorsally at roughly 50° from the horizontal, as in the titanosauriform axis from the Late Cretaceous of India described by Wilson and Mohabey ([155]; 196/CRP/GSI/05). This exceeds the 32° angle present in *Futalognkosaurus* (see Calvo et al. [156]:fig. 5) but is less than the 70° angle in *Saltasaurus* (Powell [46]:fig. 4a). The left centropostzygapophyseal lamina is stout, convex, and subcircular in section. The postzygapophyses project well beyond the caudal edge of the neural spine, more so than in the axis of many other sauropods, including other titanosaurs (see, for example, Wilson and Mohabey [155]:fig. 4). Their articular surfaces are caudolaterally oriented.

#### Third cervical vertebra

The cranial end of the third cervical vertebra (Fig 24) is preserved in partial articulation with the axis. The preserved portion includes part of the cranial articular condyle, which is conical and delineated from the remainder of the centrum by a ridge. The neural canal opens just dorsal to the centrum. In lateral view, the robust, cranially-projecting prezygapophyses are roughly triangular and subhorizontal. This last condition contrasts with that in the putative third cervical vertebra of *Malawisaurus* (MAL-243 [32]), in which the prezygapophyses are oriented craniodorsally at an angle of approximately 30°. Craniodorsally-angled prezygapophyses also present in the third cervical vertebrae of *Erketu* [157], *Maxakalisaurus* [36], *Mongolosaurus* [37,38], and probably *Rapetosaurus* (see Curry Rogers [154]:fig. 7c). The prezygapophyseal articular facets are flat and craniomedially oriented to articulate with the postzygapophyses of the axis. The robust cranial ends of the prezygodiapophyseal and spinoprezygapophyseal laminae extend caudally from the prezygapophyses.

#### Fifth cervical vertebra

**Fig 25:**
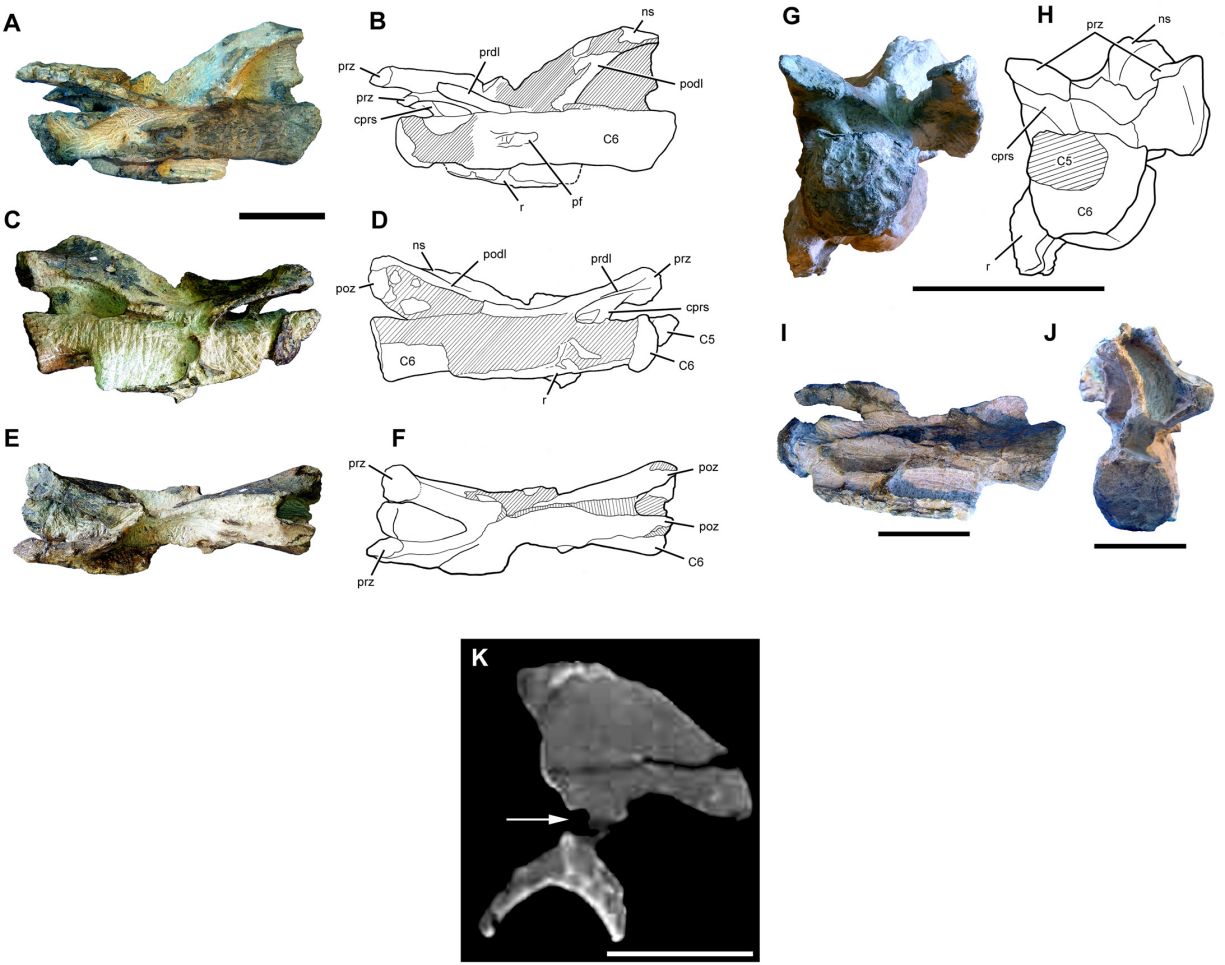
Cervical vertebra 6 of *Sarmientosaurus musacchioi* gen. et sp. nov. (MDT-PV 2). Photographs (**A**, **C, E**, **G**, **I**, **J**), interpretive drawings (**B**, **D**, **F**, **H**), and computed tomography image (**K**) in left lateral (**A**, **B**), right lateral (**C**, **D**), dorsal (**E**, **F**), cranial (**G**, **H**), ventral (**I**), and caudal (**J**) views. (**K**) Transverse section showing extent of lateral pneumatic fossa of centrum (arrow). Abbreviations see text. Scale bars = 10 cm in A–J; 5 cm in K.

Only a diminutive fragment of the centrum of the fifth cervical vertebra (Fig 25) is preserved, affixed to the cranial articular condyle of the sixth cervical. Its broken margins reveal that its internal tissue structure was camellate.

#### Sixth cervical vertebra

The sixth cervical vertebra of *Sarmientosaurus* (Fig 25) has a very low and elongate, opisthocoelous centrum (see Table 4). Many areas of the centrum are damaged, including the cranial half of the left lateral surface, the entire right lateral surface, and the central part of the ventral surface, in the area where the missing left cervical rib would have attached. In the neural arch, the dorsal and ventral extremes of the neural spine are damaged, the postzygodiapophyseal laminae are distorted, and the left postzygapophysis is not preserved.

**Table 4:** Measurements (mm) of cervical vertebrae of MDT-PV 2, the holotype of *Sarmientosaurus musacchioi* gen. et sp. nov. Abbreviation: C, cervical vertebra. * = element incomplete, measurement as preserved.

Dimension	Axis	C6	C7
Craniocaudal length, centrum	88*	316	190*
Dorsoventral height, centrum (at level of articular condyle)	---	55.9	68.2
Transverse width, centrum (at level of articular condyle)	---	61.2	65.0
Dorsoventral height, caudal articular cotyle	---	76	---
Transverse width, caudal articular cotyle	---	58	---
Craniocaudal length, prezygapophyses	---	110	---
Craniocaudal length, lateral pneumatic fossa	---	37*	22
Proximodistal length, cervical rib	---	145	---
Elongation Index (*sensu* Wedel et al. [94])	---	4.15	---
Elongation index (*sensu* Upchurch [142])	---	5.44	---

The cranial articular condyle is subspherical, slightly wider than high, and smaller than that of the seventh cervical vertebra. As mentioned above, a small piece of the caudal end of the fifth cervical centrum is attached to its cranial surface. The caudal articular cotyle of the sixth cervical vertebra is higher than wide and only gently concave, presumably because its rim is eroded. Remnants of the incomplete parapophysis and diapophysis are preserved on the left lateral side of the centrum. Between them, a small lateral pneumatic fossa (‘pleurocoel’) is present as a craniocaudally elongate depression that is partially covered by sediment. It is larger than that present in the seventh cervical vertebra. Unlike the condition in *Rapetosaurus* [154], it is bordered dorsally by a low crest. Cross sections generated by CT scans (Fig 25K) reveal this fossa to be a deep, transversely rectangular cavity; it is, however, not as extensive as that of the seventh cervical vertebra. The preserved surface of the centrum is cracked and pitted by erosion. The neural arch is low, with stout, cranially-projecting prezygapophyses. The right prezygapophysis is oriented nearly horizontally relative to the centrum, but the left is deformed and rotated dorsally. The subcircular articular facet is craniomedially oriented and surpasses the cranial articular condyle of the centrum by a few centimeters. The prezygapophyses are supported ventrally by structures that we interpret as modified centroprezygapophyseal laminae. Interestingly, instead of comprising continuous sheets of bone as in other sauropods, these structures are perforated caudally, rendering them similar to ‘pillars’ or ‘struts’ that are roughly ellipsoidal in cross section. Although this condition could conceivably be due to the taphonomic loss of a hypothetical, mediolaterally thin caudal portion of a more ‘standard’ centroprezygapophyseal lamina, we regard this possibility as unlikely given the presence of a closely comparable ‘pillar’ in the seventh cervical vertebra as well (see below). Consequently, we consider ‘centroprezygapophyseal pillars’ in the middle cervical vertebrae to be a diagnostic feature of *Sarmientosaurus*. Parts of the low spinoprezygapophyseal laminae are preserved dorsally. The right postzygapophysis has a subcircular, ventrolaterally-oriented articular facet, and the incomplete neural spine was seemingly low and undivided. The postzygodiapophyseal laminae are also incomplete. The spinopostzygapophyseal laminae caudally delimit a cavity, the spinopostzygapophyseal fossa of Wilson et al. [158], that is wide ventrally but narrows dorsally.

The low and elongate morphology of this *Sarmientosaurus* cervical vertebra (Table 4) is comparable to that present in some other titanosauriform taxa, such as *Erketu* [157,159], *Malawisaurus* [32], *Phuwiangosaurus* [41,160], *Sauroposeidon* [94,161], *Trigonosaurus* [162], and ‘Peirópolis Series A’ [7,153]. In this vertebra of the new Patagonian titanosaur, the ratio between the craniocaudal length of the centrum and the dorsoventral height of its caudal articular cotyle (the ‘elongation index’ as defined by Wedel et al. [94]) is 4.15. Similarly, when Upchurch’s [142] original definition of the ‘elongation index’ (centrum length divided by transverse cotyle width) is applied, the value for the sixth cervical of *Sarmientosaurus* is 5.44. These values are comparatively high (see Ksepka and Norell [157]), similar to those of the middle cervical vertebrae of *Tapuiasaurus* [14]. The neural arch of the sixth cervical vertebra of *Sarmientosaurus* is also substantially lower than those of similarly-positioned vertebrae of many other titanosaurs, such as *Atsinganosaurus* [163], *Isisaurus* [164], *Maxakalisaurus* [36], and *Muyelensaurus* [39].

#### Seventh cervical vertebra

**Fig 26:**
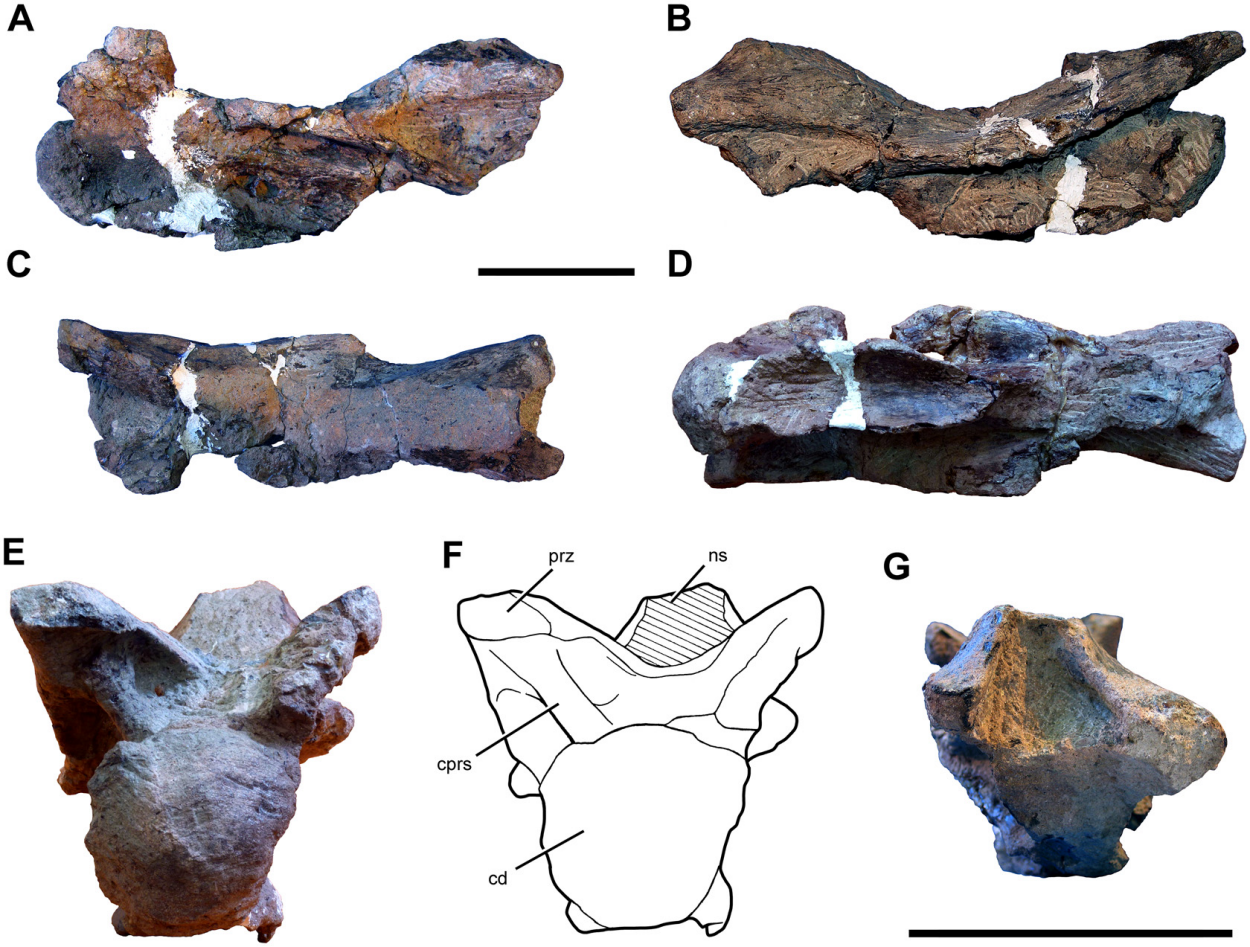
Cervical vertebra 7 of *Sarmientosaurus musacchioi* gen. et sp. nov. (MDT-PV 2). Photographs (**A**–**E**, **G**) and interpretive drawing (**F**) in left lateral (**A**), right lateral (**B**), dorsal (**C**), ventral (**D**), cranial (**E**, **F**), and caudal (**G**) views. Abbreviations see text. Scale bars = 10 cm.

The caudal part of the centrum of the seventh cervical vertebra (Fig 26) is missing, and the zygapophyses and part of the neural spine are damaged. The centrum has a convex cranial articular condyle and deeply excavated lateral faces, and its ventral surface is markedly concave transversely. On the left lateral surface there is a small, craniocaudally elongate pneumatic fossa that is partitioned by a lamina into a cranial portion and a slightly larger caudal portion. On the right side of the centrum, the external margin of the pneumatic fossa is eroded. Nevertheless, CT images demonstrate that the centrum has a minimum transverse thickness of only ~6.5 mm here; as such, in this region, the centrum consists only of a median lamina that separates the two large pneumatic fossae. A similar morphology is present in a cervical vertebra of a juvenile individual of *Phuwiangosaurus* [165] and in *Erketu*, an especially long-necked somphospondylan from the Early Cretaceous of Mongolia. In this latter taxon, the lateral surfaces of each cervical centrum are excavated by well-developed pneumatic fossae that reduce the centrum to a thin median septum [157,159].

Only traces of the parapophyses are preserved in the seventh cervical vertebra of *Sarmientosaurus*. The zygapophyses are also incomplete. The more complete right prezygapophysis is stoutly constructed, and, as in the sixth cervical vertebra, it is supported ventrally by a ‘centroprezygapophyseal pillar.’ Parts of the robust prezygodiapophyseal lamina and the postzygodiapophyseal lamina are preserved on the right side of the neural arch. The preserved portions of the postzygapophyses are similar in construction to those of the sixth cervical vertebra. The neural spine seems to be considerably more caudally situated than in comparably-positioned cervical vertebrae of most other titanosaurs (e.g., *Atsinganosaurus*, *Futalognkosaurus*, *Isisaurus*, *Malawisaurus*, *Maxakalisaurus*, *Muyelensaurus*, *Rinconsaurus*, *Saltasaurus*, *Uberabatitan*).

#### Cervical ribs

As noted above, much of the caudal process of the right cervical rib of the axis is preserved and separated into two fragments (Fig 24A and 24B). The right rib of the sixth cervical vertebra is best observed in medial view (Fig 25). As seen in right lateral view, parts of its tuberculum and capitulum are preserved, and both of these structures are delicately constructed. The caudal process of the rib is thin and tubular. The cranial process narrows cranially but its tip is not preserved.

#### Ossified cervical tendon

**Fig 27:**
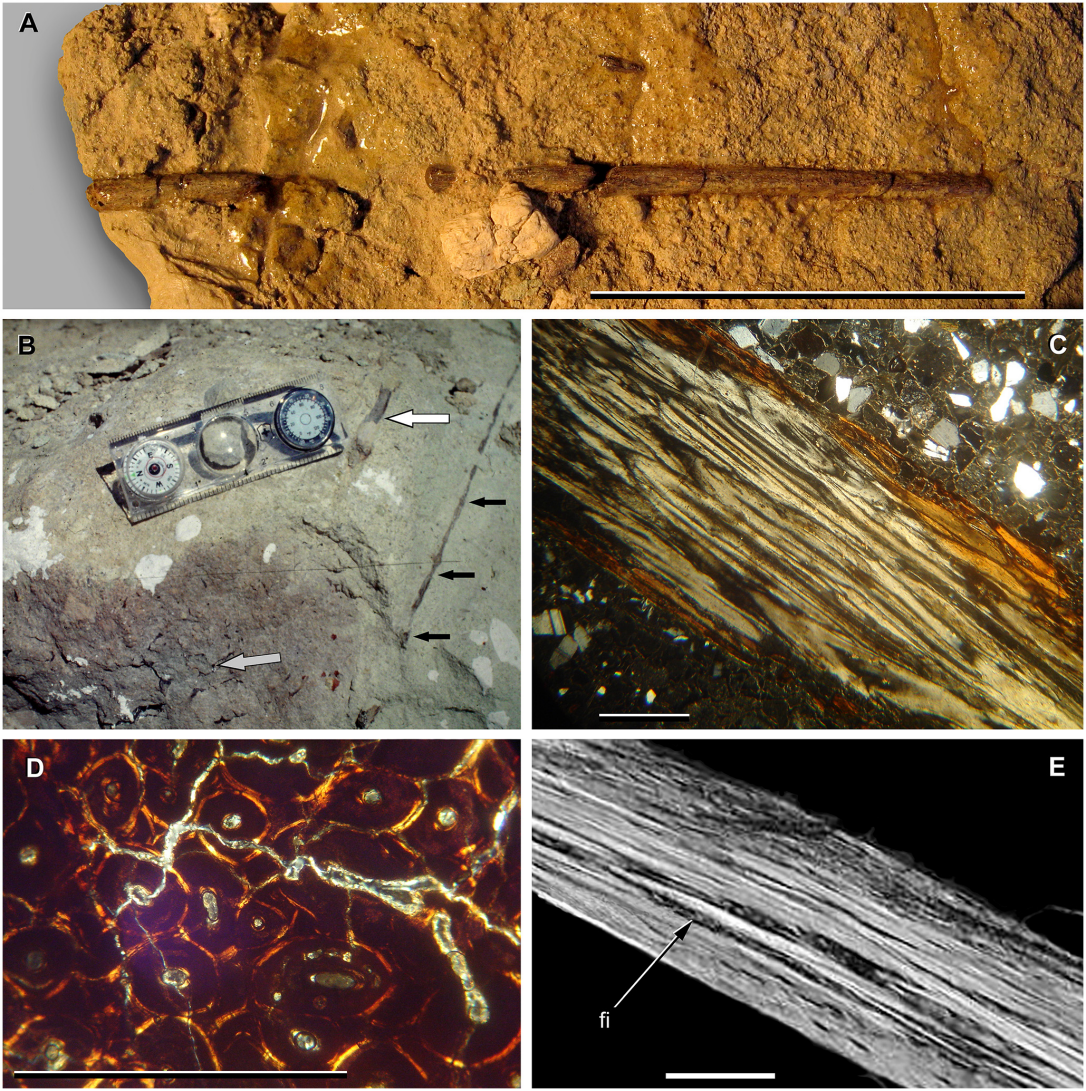
Ossified cervical tendon of *Sarmientosaurus musacchioi* gen. et sp. nov. (MDT-PV 2). (**A**) Section of tendon. (**B**) Field photograph showing relative dispositions of the camellate tissue of a cervical vertebra (hatched arrow), a cervical rib (white arrow) and the cervical tendon (black arrows) upon discovery. (**C**) Longitudinal thin-section of tendon showing longitudinal structures compatible with fibroblasts (near the top of the thin-section). (**D**) Transverse thin-section showing Haversian tissue. (**E**) Longitudinal thin-section (crossed polars, 100×) of tendon of *Podilymbus* (grebe) showing fibroblasts (modified from Organ and Adams [174]). Abbreviation see text. Scale bars = 5 cm in A; 1 mm in C–D; 0.1 mm in E.

An enigmatic, cable-like structure was observed *in situ* at the type locality of *Sarmientosaurus*, ventrolateral to the right side of the cervical vertebrae and ribs. The structure originated immediately caudal to the skull and coursed along multiple vertebrae, maintaining a constant diameter of only ~3 mm throughout its length (Figs 2, 27A and 27B). This delicate structure is clearly distinct from the cervical ribs, which are much thicker and occupied a different position (Fig 27B). The structure is nearly oval in section and its external surface is rugose and striated (Fig 27A). As seen in thin section, its microstructure is composed of bone with numerous Haversian canals and longitudinally oriented fibers (Fig 27C and 27D). These fibers are comparable to the fibroblasts of tendinous tissue [166] that are found in the tendons of some extant birds (Fig 27E). Consequently, we regard this peculiar structure of *Sarmientosaurus* as an ossified cervical tendon. Sauropod cervical ribs are known to be derived from ossified tendons [166,167], raising the possibility that the structure corresponds to the distal part of one or more of these ribs. Nevertheless, unquestionable cervical ribs attached to the axis and all more caudal cervical vertebrae were also observed in the quarry; these were thicker and positioned differently, and they each decreased in diameter caudally. Furthermore, as above, the enigmatic tendon of *Sarmientosaurus* originated adjacent to the occipital region of the skull and extended for several meters without interruption or an appreciable change in diameter.

**Fig 28:**
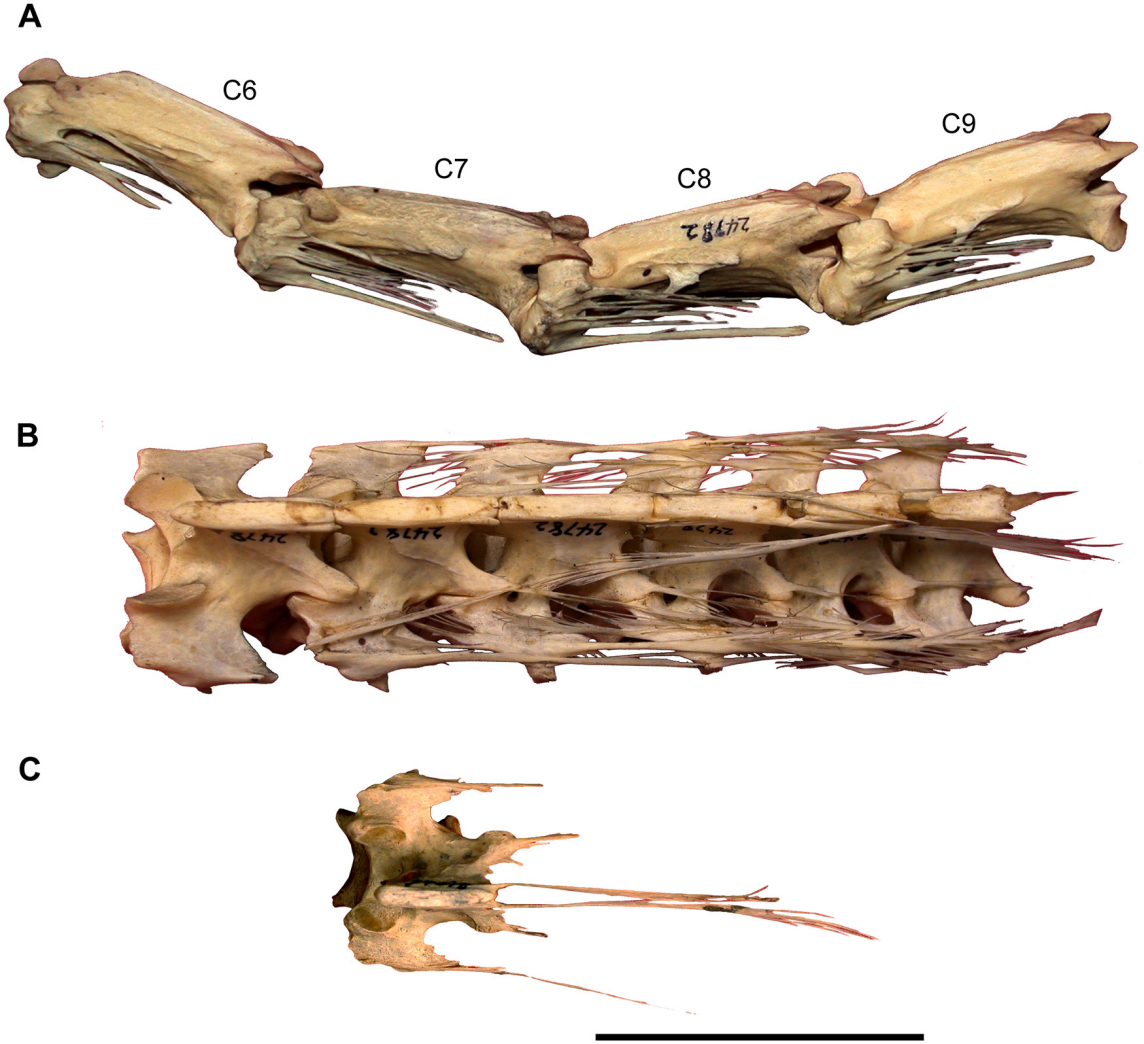
Ossified tendons in presacral vertebrae of extant Gruidae (cranes). (**A**) *Grus antigone* (ANS 24782), articulated cervical vertebrae 6–9 and associated ossified tendons in left lateral view. (**B**) *Grus antigone* (ANS 24782), articulated cranial thoracic vertebrae and associated tendons in dorsal view. (**C**) *Anthropoides paradiseus* (ANS 37018), thoracic vertebra 1 and associated tendons in dorsal view. Photos courtesy Jerry Harris, Dixie State University. Scale bar = 5 cm.

Except *Sarmientosaurus*, no sauropods (or non-avian dinosaurs in general) are known to have ventrolaterally-situated, ossified cervical tendons; nevertheless, the position and morphology of the structure in question is consistent with this hypothesized identification. One line of evidence that may support this identity is the fact that many modern birds have very elongate cervical muscles, and that some of these birds have ossified tendons situated deep within the neck musculature. The latter occurs in Gruidae (cranes; e.g., *Anthropoides paradiseus*, *Grus antigone*), in which there are many ossified cervical tendons; nevertheless, in these birds, the tendons extend the length of one or only a few presacral centra (Fig 28). The enigmatic tendon of *Sarmientosaurus* could conceivably be part of an insertion tendon of M. rectus capitis anterior (ventralis), which connects the cranial cervical centra with the basal tubera of the skull in extant diapsids [168,169]. In modern taxa, however, this tendon is not ossified, and it is usually wide rather than cable-like as in MDT-PV 2 (T. Tsuihiji, pers. comm.). Another possibility is that the structure is an insertion tendon of M. longus colli ventralis or Mm. intertransversarii, but not extending along the entire neck (J. Harris, pers. comm.). Histologically, the tendon shows significant secondary remodeling, suggesting that it ossified early in ontogeny, probably long before the sauropod attained its maximum size. Moreover, ontogenetic studies of other archosaurs (e.g., ornithischian dinosaurs) indicate that tendons often remain ossified throughout the ontogeny of these animals. As such, the ossification of this tendon in *Sarmientosaurus* is probably not attributable to the seemingly advanced ontogenetic stage of the holotype (I. Cerda, pers. comm.).

In sum, several facts support the hypothesis that the enigmatic structure in the neck of *Sarmientosaurus* is an ossified tendon that is distinct from the cervical ribs. The structure maintained a diameter of only ~3 mm through a length of at least 3 m, and is histologically consistent with ossified tendon. Furthermore, unquestionable cervical ribs are associated with the axis and sixth cervical vertebra on the right side. The function of this presumed ossified tendon is unknown.

## Discussion

### Distinctive Features of *Sarmientosaurus*

#### Proportions of orbit

In *Sarmientosaurus*, the maximum diameter of the orbit is nearly 40% the rostrocaudal length of the cranium (with the latter measured from the tip of the snout to the occipital condyle). By contrast, in the basal macronarian *Camarasaurus*, the maximum orbital diameter is only 26% of cranial length. In the brachiosaurid *Giraffatitan*, this value is 21%, and in the Brazilian lithostrotian titanosaur *Tapuiasaurus* it is 20%. With a maximum diameter of about 35% total cranial length, only the orbit of the Early Cretaceous titanosauriform *Abydosaurus* approaches the proportionally large size seen in *Sarmientosaurus*.

#### Maxilla—lacrimal articulation

*Sarmientosaurus* has a complex articulation between the maxilla and the lacrimal: the ascending ramus of the maxilla is embedded in and bordered medially and laterally by the dorsal process of the lacrimal. This unusual configuration is not present in *Camarasaurus*, in which the maxillary ascending ramus contacts the nasal and lacrimal, nor in *Giraffatitan*, where this ramus mostly articulates with the nasal, and to a lesser extent, the lacrimal. In the Madagascan titanosaur *Rapetosaurus*, the ascending ramus appears to overlap the rostral half of the lacrimal laterally and dorsally [13]. In *Tapuiasaurus*, however, the lacrimal appears to have a complex relationship with the maxillary ascending ramus that is reminiscent of that in *Sarmientosaurus*. The left ascending ramus (the right is damaged) is bordered by the lacrimal both laterally and medially, although the medial exposure of the latter (on the lateral margin of the bony nasal aperture) is proportionally much greater than in *Sarmientosaurus*. Nevertheless, this region of the skull is significantly distorted in *Tapuiasaurus*, and as such its true morphology remains in doubt.

#### Narial ridge

In MDT-PV 2, the medial edge of the ascending ramus of the maxilla exhibits a low but distinct ridge that borders the bony nasal aperture. This narial ridge is not present in *Abydosaurus*, *Camarasaurus*, *Giraffatitan*, or *Rapetosaurus*. *Tapuiasaurus* appears to show a subtle ridge in the same or a closely similar position as that in *Sarmientosaurus* (see Zaher et al. [14]:fig. 1c), but further observations of the holotype of this Brazilian titanosaur (MZSP-PV 807) are required to ascertain this. The ascending ramus of the maxilla and the margin of the bony nasal aperture are not preserved in *Nemegtosaurus*.

#### Caudoventral process of quadratojugal

The quadratojugal of *Sarmientosaurus* is distinctive in that its caudoventral corner is attenuated into a caudomedially-projecting, rounded, ‘tongue-like’ process that caudally overlaps the ventrolateral extreme of the quadrate and comprises the ventrolateral margin of the quadrate fossa. This process is not observed in most other macronarians (e.g., *Camarasaurus*, *Giraffatitan*, *Nemegtosaurus*, *Quaesitosaurus*); in these taxa, no part of the quadrate is obscured from caudal view by the quadratojugal. A comparable process may be present in *Tapuiasaurus* and possibly *Rapetosaurus* (see Zaher et al. [14]:5), but if so, it is significantly less developed than in *Sarmientosaurus* (see Curry Rogers and Forster [13]:fig. 17b; Zaher et al. [14]:fig. 1d). We therefore consider this ‘tongue-like’ quadratojugal process to be an autapomorphy of the new Patagonian titanosaur.

#### Tooth inclination

The only known specimen of *Sarmientosaurus* has noticeably procumbent maxillary teeth and recumbent dentary teeth. This combination of tooth inclinations is not observed in any other sauropod, although procumbent maxillary and dentary teeth are known in the diplodocid *Diplodocus* and the macronarians *Camarasaurus* and *Euhelopus* [99,101,111]. It could be argued that the combination of tooth orientations seen in MDT-PV 2 is an artifact of lithostatic pressure applied to the skull. Nevertheless, it is difficult to envision a mode of taphonomic deformation that (1) would cause most maxillary and dentary teeth to ‘lean’ mesially and distally, respectively, while simultaneously keeping the premaxillary and mesialmost maxillary teeth subvertical, and that (2) would not cause obvious mediolateral distortion or damage to the tooth crowns.

#### Centroprezygapophyseal ‘struts’ in cervical vertebrae

The middle cervical vertebrae of *Sarmientosaurus* are unique among Sauropoda in having centroprezygapophyseal laminae that have been modified into structures resembling ‘struts’ or ‘pillars.’ In *Camarasaurus*, the centroprezygapophyseal laminae are continuous bony sheets that connect the ventral surfaces of the prezygapophyses to the dorsal surface of the centrum. This is also the case in the non-titanosaurian titanosauriforms *Erketu* and *Sauroposeidon* [170,171] and the titanosaurs *Malawisaurus*, *Rapetosaurus*, *Saltasaurus*, and *Trigonosaurus*. In the brachiosaurid *Giraffatitan* [172], the cranial sector of the centroprezygapophyseal lamina is solid; an infraprezygapophyseal fossa is present more caudally, but the lamina is never perforated to form a ‘strut’ or ‘pillar’ as in *Sarmientosaurus*.

#### Ossified cervical tendon

The new Patagonian titanosaur appears to possess a very elongate, ossified tendinous structure that extends along the cervical series, ventrolateral to the vertebrae and ribs. Ossified tendons are common in various groups of non-avian and avian dinosaurs [173,174], including sauropods. Bony rods found in sauropod quarries have traditionally been regarded as the ends of the elongate cervical ribs of these animals. In their studies of the histogenesis of sauropod cervical ribs, Cerda [166] and Klein et al. [167] concluded that the elongate portion of the rib arose from the ossification of a tendinous element. Nevertheless, during the recovery of MDT-PV 2, the two of us who were present (R.D.F.M. and G.A.C.) observed a dark, cylindrical structure that originated approximately 5 cm from the right quadrate and extended uninterrupted for several meters while maintaining a constant diameter of only ~3 mm. Simultaneously, we observed ‘typical’ ribs attached to the axis and sixth cervical vertebra, which were much thicker and lighter in color. The enigmatic ossified cervical tendon of *Sarmientosaurus* is presently unique among Dinosauria.

### Phylogenetic Relationships

We investigated the evolutionary relationships of *Sarmientosaurus* via two principal phylogenetic analyses, both of which entailed multiple iterations of analysis with TNT (Tree analysis using New Technology) v. 1.1 [175].

In the first principal analysis, we examined the interrelationships of 22 sauropod genera using the basal form *Shunosaurus* as an outgroup. Ingroup taxa included *Abydosaurus*, *Alamosaurus*, *Apatosaurus*, *Baurutitan*, *Camarasaurus*, *Diplodocus*, *Epachthosaurus*, *Euhelopus*, *Giraffatitan*, *Jobaria*, *Malawisaurus*, *Nemegtosaurus*, *Neuquensaurus*, *Nigersaurus*, *Omeisaurus*, *Phuwiangosaurus*, *Rapetosaurus*, *Rocasaurus*, *Saltasaurus*, *Sarmientosaurus*, *Tapuiasaurus*, and *Trigonosaurus*. The 337 characters (105 cranial, 232 postcranial) were compiled by the second author (M.C.L.). Characters were drawn or modified from many previous analyses [2,7,12,22,24,34,39,44,88,90,103,115,124,142,156,176–185], with a few of these [90,103,142,180] being the original sources of most characters. Twenty-six characters (numbers 3, 6, 14, 18, 20, 35, 50, 58, 63, 70, 75, 83, 90, 94, 98, 137, 160, 166, 200, 211, 212, 247, 254, 289, 290, and 322) were treated as ordered. The character list and data matrix are provided in the Supporting Information, as S1 and S2 Appendix, respectively.

**Fig 29:**
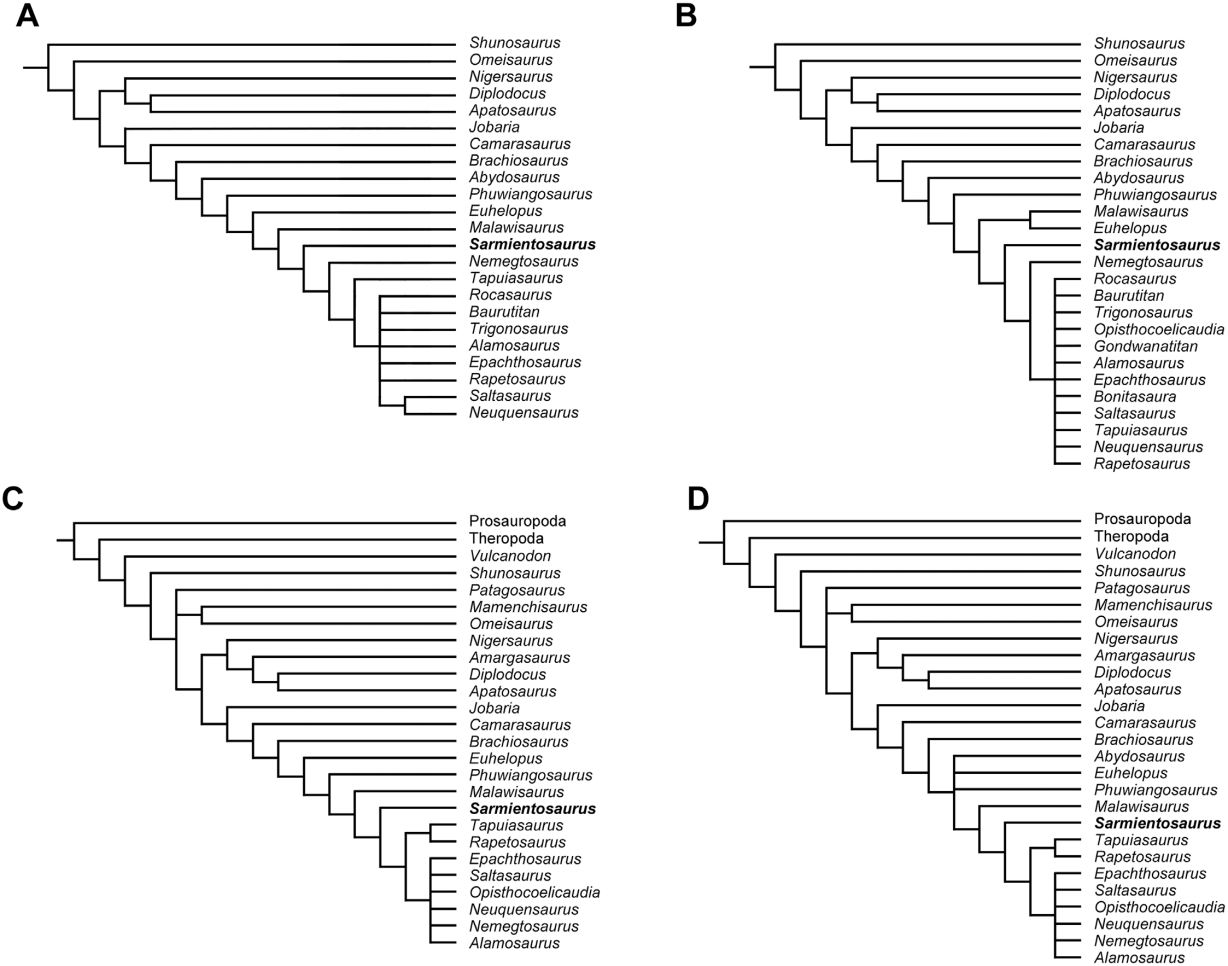
Hypothesized phylogenetic position of *Sarmientosaurus musacchioi* gen. et sp. nov. within Titanosauria. (**A**) Strict consensus of three trees of 590 steps based on initial analysis of matrix of 22 ingroup taxa and 337 osteological characters. (**B**) Strict consensus of 32 trees of 536 steps based on analysis of modified matrix of 25 ingroup taxa and 295 osteological characters. (**C**) Strict consensus of seven trees of 434 steps based on analysis of matrix of 25 ingroup taxa and 246 osteological characters (modified from Zaher et al. [14]). (**D**) Strict consensus of 14 trees of 447 steps based on analysis of matrix used in (**C**) with the addition of the titanosauriform *Abydosaurus*. All analyses recover *Sarmientosaurus* as a basal lithostrotian titanosaur, intermediate in phylogenetic position between the basalmost lithostrotian *Malawisaurus* and more derived titanosaurs.

The initial analysis (traditional [heuristic] search, 1,000 replicates of Wagner trees, random addition sequence, tree bisection reconnection branch swapping algorithm, ten trees saved per replicate) yielded three most parsimonious trees of 590 steps in length, the strict consensus of which has a Consistency Index (CI) of 0.55 and a Retention Index (RI) of 0.61. In this consensus tree, *Sarmientosaurus* is placed in a derived position relative to *Phuwiangosaurus*, *Euhelopus*, and the basal lithostrotian titanosaur *Malawisaurus*, as the sister taxon of the more derived lithostrotians in the analysis, the most derived of which form an unresolved polytomy (Fig 29A).

To further investigate the phylogenetic relationships of *Sarmientosaurus*, we altered the dataset by adding the titanosaurs *Bonitasaura* and *Opisthocoelica udia* to the matrix. Initial analysis of this augmented matrix (S3 Appendix) yielded four most parsimonious trees of 613 steps, the strict consensus of which has a CI of 0.54 and an RI of 0.62. The proposed position of *Sarmientosaurus* relative to other titanosaurs remained unchanged, though the topology among more derived members of Lithostrotia was slightly altered.

In an effort to improve recovered phylogenetic resolution within Titanosauria, we removed 42 characters from the original matrix (characters 7–10, 12, 30, 37, 42, 44, 48, 58, 60–62, 70, 72, 87–90, 107, 108, 124, 126, 129, 131, 141, 147, 150, 157, 158, 160, 163, 164, 183, 184, 187, 193, 232, 269, 277, and 296), including many that had been optimized as synapomorphies of Neosauropoda in previous iterations of the analysis and others that were poorly informative. We also added the Brazilian titanosaur *Gondwanatitan* [186] to the matrix, thus totaling 25 ingroup taxa and 295 characters (S4 Appendix). The analysis yielded 32 most parsimonious trees of 536 steps, the strict consensus of which (Fig 29B) has a CI of 0.51 and an RI of 0.60. Yet again *Sarmientosaurus* is hypothesized as a lithostrotian more derived than *Phuwiangosaurus*, *Euhelopus*, and *Malawisaurus* (the latter two of which form a clade); in turn, the new Patagonian sauropod is proposed as the sister taxon of all other titanosaurs in the analysis.

The second principal analysis was performed using the phylogenetic data matrix published by Zaher et al. [14], which included an ingroup of 31 sauropod genera and Theropoda plus Prosauropoda as an outgroup. Ingroup genera included *Alamosaurus*, *Amargasaurus*, *Apatosaurus*, *Barapasaurus*, *Barosaurus*, *Camarasaurus*, *Diamantinasaurus*, *Dicraeosaurus*, *Diplodocus*, *Euhelopus*, *Giraffatitan*, *Haplocanthosaurus*, *Isisaurus*, *Jobaria*, *Malawisaurus*, *Mamenchisaurus*, *Nemegtosaurus*, *Neuquensaurus*, *Nigersaurus*, *Omeisaurus*, *Opisthocoelicaudia*, *Patagosaurus*, *Phuwiangosaurus*, *Rapetosaurus*, *Rayososaurus*, *Rebbachisaurus*, *Saltasaurus*, *Shunosaurus*, *Tangvayosaurus*, *Tapuiasaurus*, and *Vulcanodon*. To these, we added *Sarmientosaurus* and *Epachthosaurus*, both of which are known from Cenomanian—Turonian strata of the Lower Member of the Bajo Barreal Formation in central Patagonia. The matrix (S5 Appendix) included 246 characters (241 unordered, five [numbers 8, 37, 64, 66, and 198] ordered), 88 of which were cranial and the remaining 158 postcranial. Our initial analytical trial yielded 20 most parsimonious trees of 472 steps, the strict consensus of which has a CI of 0.56 and an RI of 0.75. In this tree, *Sarmientosaurus* is again hypothesized as a basal lithostrotian titanosaur, more derived than *Phuwiangosaurus*, *Tangvayosaurus*, and *Malawisaurus* and the sister group of the other lithostrotians in the analysis.

We then eliminated nine ingroup taxa that are represented by fragmentary fossils and/or not thought to be closely related to *Sarmientosaurus* (*Barapasaurus*, *Barosaurus*, *Diamantinasaurus*, *Dicraeosaurus*, *Haplocanthosaurus*, *Isisaurus*, *Rayososaurus*, *Rebbachisaurus*, and *Tangvayosaurus*) and conducted a second iteration of the analysis (S6 Appendix). This trial recovered seven most parsimonious trees of 434 steps, the strict consensus of which (Fig 29C) has a CI of 0.61 and an RI of 0.76. Once again, *Sarmientosaurus* is postulated as a basal lithostrotian titanosaur, more derived than *Phuwiangosaurus* and *Malawisaurus* but more basal than other Lithostrotia. Finally, we added the North American Early Cretaceous titanosauriform *Abydosaurus* to this reduced matrix, to test its potential relationship to *Sarmientosaurus* (S7 Appendix). This trial yielded 14 most parsimonious trees of 447 steps (CI = 0.59; RI = 0.74). The strict consensus of these trees (Fig 29D) posits *Abydosaurus* as a member of Somphospondyli, in an unresolved polytomy with *Euhelopus*, *Phuwiangosaurus*, and Lithostrotia at the base of the clade. Once again, *Sarmientosaurus* is postulated as a basal lithostrotian titanosaur, more derived than *Malawisaurus* but more basal than all other lithostrotians. Importantly, the new Patagonian taxon is always maintained in a considerably more basal position than is *Epachthosaurus*, which holds implications for the relationships of these taxa to one another.

### Are *Sarmientosaurus* and *Epachthosaurus* the Same Taxon?

All known fossils of the titanosaurian sauropod *Epachthosaurus* were collected from a site approximately 100 km south of that which produced *Sarmientosaurus*, from strata of the Lower Member of the Bajo Barreal Formation that are closely comparable to (and possibly correlative with) those that yielded the latter sauropod [7,67,187,188]. Regrettably, known specimens of *Epachthosaurus* and *Sarmientosaurus* do not preserve skeletal elements in common, and as such, the two taxa cannot be directly compared. Given that both are titanosaurs from nearby sites in the same geological formation, it is reasonable to consider the possibility that they may represent the same taxon.

As is the case for *Sarmientosaurus* (see above), *Epachthosaurus* has been frequently (though not universally) regarded as a basal titanosaurian. Nevertheless, the results of our phylogenetic analysis suggest that *Sarmientosaurus* occupies a substantially more basal position within Lithostrotia than does *Epachthosaurus*, casting doubt on the possibility that the two forms could be synonymous. It is, however, at least conceivable that they might represent a single titanosaurian taxon in which the skull and neck are more morphologically conservative than the remainder of the skeleton.

**Fig 30:**
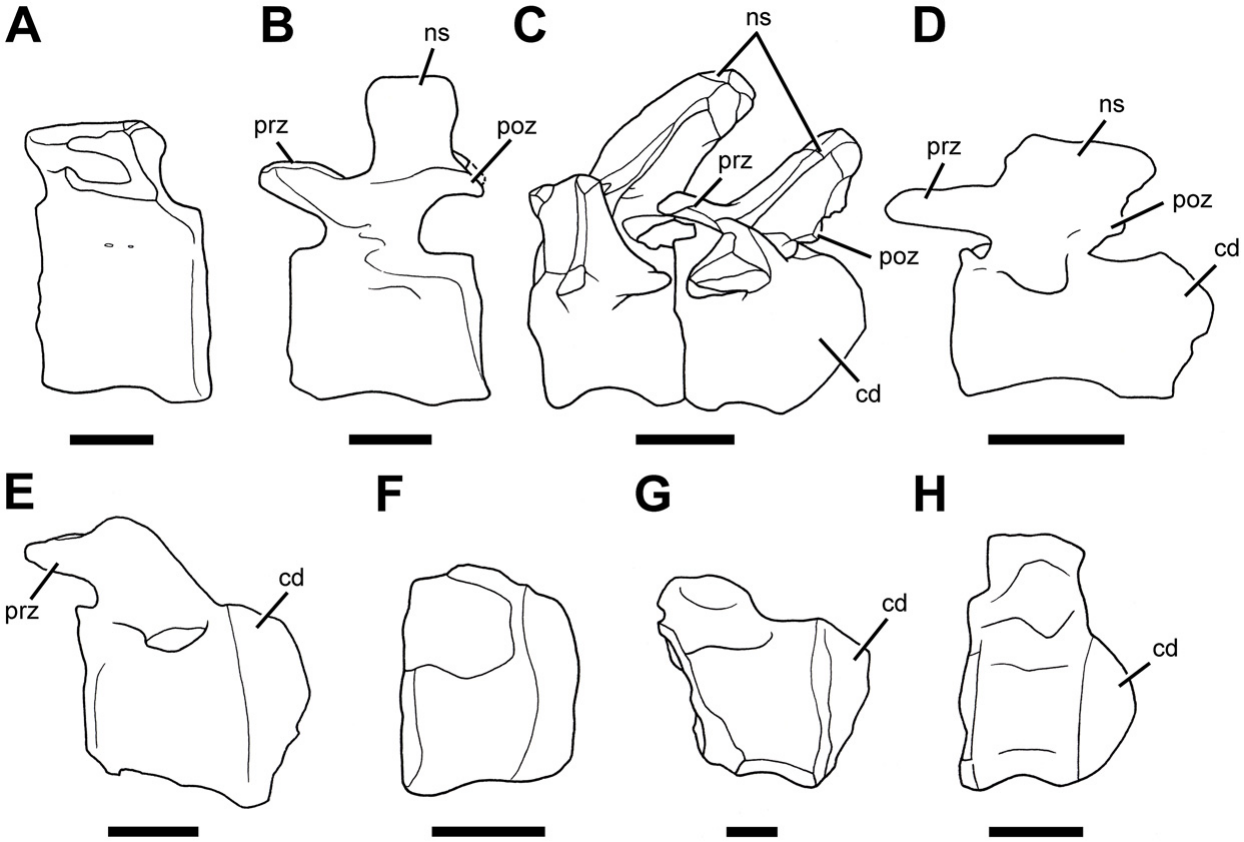
Titanosaur caudal vertebrae from the Lower Member of the Bajo Barreal Formation in left lateral view, to demonstrate the diversity of these sauropods in this geologic unit. Specimens were recovered from the Estancia Ocho Hermanos locality unless otherwise indicated. (**A**) Middle caudal vertebra of *Andesaurus*-like taxon (UNPSJB-PV 178, reversed and redrawn from Powell et al. [51]:fig. 1f). (**B**) Middle caudal vertebra of *Andesaurus*-like taxon (UNPSJB-PV 595, reversed and redrawn from Powell et al. [51]:fig. 1b). (**C**) Proximal (first and second) caudal vertebrae of *Epachthosaurus sciuttoi* (UNPSJB-PV 920, reversed and redrawn from Martínez et al. [67]:fig. 6a). (**D**) Middle (tenth) caudal vertebra of *Epachthosaurus sciuttoi* (UNPSJB-PV 920, redrawn from Martínez et al. [67]:fig. 8a). (**E**) Proximal caudal vertebra of indeterminate titanosaurian (UNPSJB-PV 876, redrawn from Powell et al. [51]:fig. 2a). (**F**) Proximal or middle caudal vertebra of indeterminate titanosaurian (UNPSJB-PV 182, redrawn from Powell et al. [51]:fig. 2e). (**G**) Middle caudal vertebra of indeterminate titanosaurian (UNPSJB-PV 730, redrawn from Powell et al. [51]:fig. 2g). (**H**) Proximal caudal vertebra of indeterminate titanosaurian (UNPSJB-PV 584, redrawn from Sciutto and Martínez [53]:fig. 4a). Unlike the others, the vertebra in (**H**) was collected from the Cañadón de Las Horquetas locality. Scale bars = 5 cm in A–B, E–G; 10 cm in C–D; 6 cm in H.

**Fig 31:**
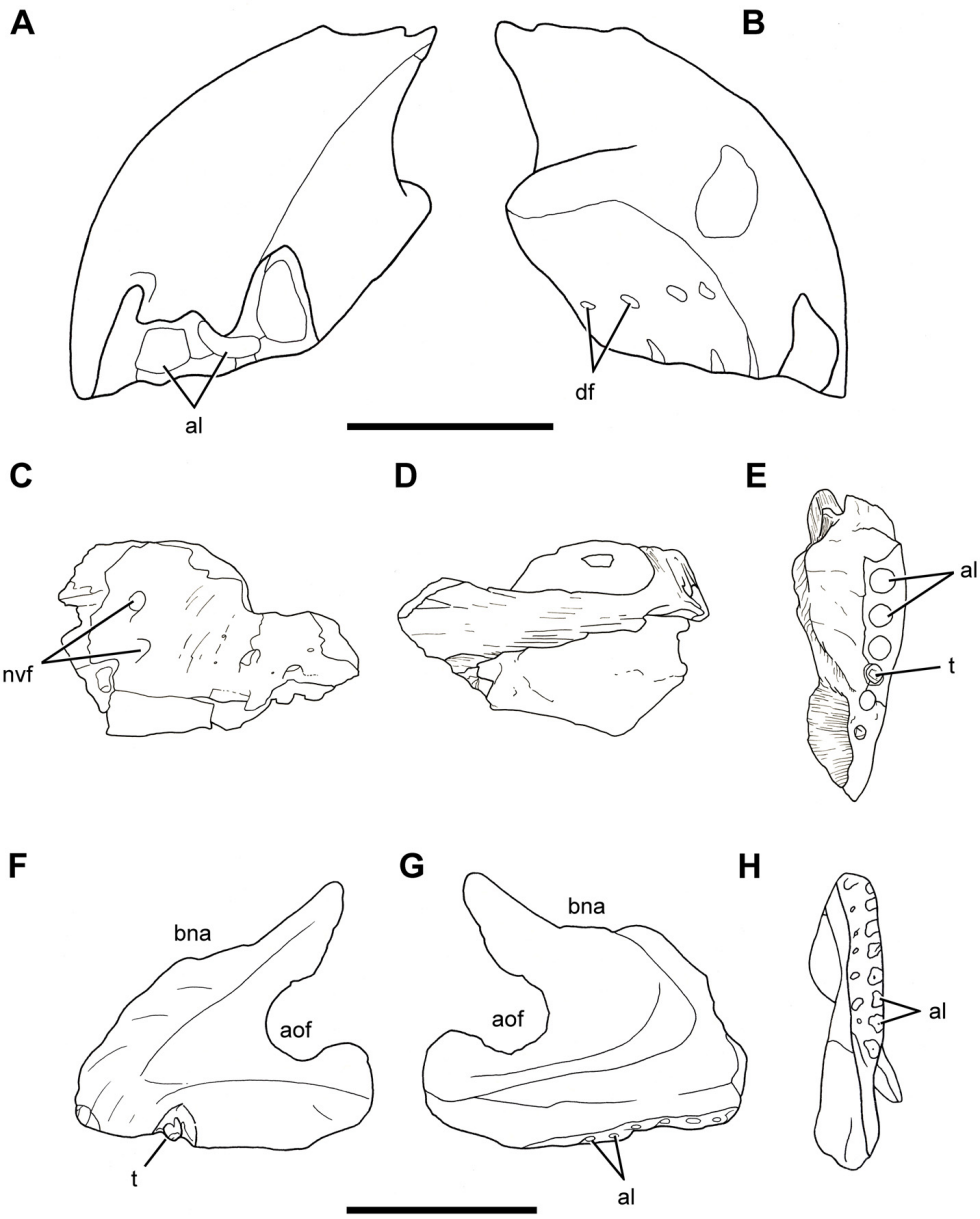
Isolated titanosaur cranial elements from the Cretaceous Chubut Group of southern Chubut Province, central Patagonia, Argentina. Left premaxilla (UNPSJB-PV 669) in lateral (**A**) and medial (**B**) views (modified and redrawn from Powell et al. [51]:fig. 1h, i). Partial left maxilla of *Campylodoniscus ameghinoi* (MACN A-IOR63) in lateral (**C**), medial (**D**), and ventral (occlusal, **E**) views (modified and redrawn from Huene [9]:pl. 40, fig. 1a–c). Left maxilla (UNPSJB-PV 583) in lateral (**F**), medial (**G**), and ventral (occlusal, **H**) views (modified and redrawn from Sciutto and Martínez [53]:fig. 7; note that a single tooth has been exposed via preparation since that paper was published). Specimens UNPSJB-PV 669 and 583 were unambiguously recovered from the Lower Member of the Bajo Barreal Formation, at the Estancia Ocho Hermanos and Cañadón de Las Horquetas localities, respectively. MACN A-IOR63 was collected from the western flank of the Sierra de San Bernardo west of Lago Musters (i.e., in the vicinity of the Estancia Ocho Hermanos); unfortunately, however, its stratigraphic position within the Chubut Group is not known [7,9]. Scale bars = 5 cm in A–E; 10 cm in F–H.

Another point that should be considered in this context is demonstrated in Table 5: titanosaurian sauropods are remarkably diverse in the Cenomanian—Turonian strata of the Lower Member of the Bajo Barreal Formation that produced *Epachthosaurus* and *Sarmientosaurus*. In addition to these two forms, comparable levels of this formation exposed in northern Santa Cruz Province (Argentina) have yielded a third titanosaurian taxon, *Drusilasaura* [73], and slightly younger (Turonian—Coniacian; G.A.C., unpublished data) beds in southern Chubut have produced a fourth titanosaur, *Elaltitan* [7,106,189]. Furthermore, the locality in southern Chubut Province that yielded known material of *Epachthosaurus* (the Estancia Ocho Hermanos; see Martínez et al. [67]) has also produced the following titanosaurian specimens from comparable levels of the Bajo Barreal Formation: (1) a series of caudal vertebrae similar in morphology to those of the basal titanosaurian *Andesaurus* (e.g., UNPSJB-PV 178 and 595 [51]; Fig 30A and 30B); (2) another partial caudal series (UNPSJB-PV 175, 176, 177, 876, and 877 [51]; Fig 30E) associated with two hemal arches and a fragmentary femur (UNPSJB-PV 172) of a taxon that is clearly distinct from *Epachthosaurus* [51] (Fig 30C and 30D); and (3) a left premaxilla (UNPSJB-PV 669 [51]) that is proportionally dorsoventrally taller and mediolaterally wider than those of *Sarmientosaurus* (Fig 31A and 31B). In addition, Sciutto and Martínez [53] described a titanosaurian left maxilla (UNPSJB-PV 583; Fig 31F and 31H) from a locality very close to that which yielded MDT-PV 2, and from an equivalent horizon of the Bajo Barreal Formation. This maxilla differs strongly from those of *Sarmientosaurus* in several features: (1) it is relatively rostrocaudally short and dorsoventrally tall; (2) it preserves the margin of the narial opening, which was clearly more rostrally placed than in *Sarmientosaurus*; (3) it has only eight alveoli (versus 11–12 in *Sarmientosaurus*); and (4) its teeth are narrower-crowned. This demonstrates that at least one other titanosaur with a comparatively short and high skull coexisted with *Sarmientosaurus*.

**Table 5:** Fossil vertebrate fauna of the Upper Cretaceous (Cenomanian—Turonian) Bajo Barreal Formation of central Patagonia, Argentina. Note that several taxa previously assigned to this formation (e.g., the titanosaurian sauropods *Aeolosaurus colhuehuapensis*, *Argyrosaurus superbus*, and *Elaltitan lilloi*; the hadrosaurid ornithopod *Secernosaurus koerneri*) are now known to come from a recently defined, stratigraphically younger geologic unit, the Lago Colhué Huapi Formation [80].

**Osteichthyes**
Actinopterygii
Holostei indet.
**Testudines**
Chelidae
*Bonapartemys bajobarrealis*[232]
*Prochelidella argentinae* [232]
**Crocodyliformes**
Crocodyliformes indet.
**Pterosauria**
Pterosauria indet.
**Dinosauria**
Theropoda
Ceratosauria
Abelisauridae
*Xenotarsosaurus bonapartei* [66]
Abelisauridae indet. [69,71,233,234]
Tetanurae
Avetheropoda
Megaraptora indet. [235]
Allosauroidea
Carcharodontosauridae indet. [71]
Coelurosauria
*Aniksosaurus darwini* [70,72,236]
Dromaeosauridae indet. [71]
Sauropoda
Sauropoda indet. [51,53]
Neosauropoda
Diplodocoidea
Rebbachisauridae
*Katepensaurus goicoecheai* [75,237]
Rebbachisauridae indet. [53,238,239]
Titanosauriformes
Titanosauria
?*Andesaurus* sp. [51]
*Drusilasaura deseadensis* [73]
Titanosauria indet.[51,53,240]
Lithostrotia
*Epachthosaurus sciuttoi* [67,187,188,241]
*Sarmientosaurus musacchioi* [242,243;this paper]
Ornithopoda
*Notohypsilophodon comodorensis* [68,244]

The taxonomic situation involving *Sarmientosaurus* and *Epachthosaurus* is closely comparable to that between the titanosaurs *Nemegtosaurus* [10] and *Opisthocoelicaudia* [190] from the Upper Cretaceous Nemegt Formation of Mongolia. Both of these Nemegt titanosaurs are currently considered valid, though the former is represented only by the skull and the latter only by postcrania. As is the case for *Sarmientosaurus* and *Epachthosaurus*, definitively determining whether or not *Nemegtosaurus* and *Opisthocoelicaudia* belong to the same taxon will require the discovery of specimens that preserve an association of skeletal elements that are already known for both of these forms.

### Implications of *Sarmientosaurus* for Titanosauriform Evolution

Our cladistic analyses have consistently recovered *Sarmientosaurus* as an archaic lithostrotian titanosaur, phylogenetically basal to taxa such as *Nemegtosaurus*, *Rapetosaurus*, and *Tapuiasaurus*. The phylogenetic position of the new Patagonian form relative to *Tapuiasaurus* is of particular interest given the respective geologic ages of these sauropods. *Tapuiasaurus* comes from Aptian (possibly early Aptian) strata in Brazil [14], whereas *Sarmientosaurus* is from Cenomanian—Turonian beds in southern Argentina. According to the current Geologic Time Scale [65], the Aptian extends from 126–113 Ma and the Cenomanian—Turonian from 100–89.8 Ma. Consequently, the comparatively derived Brazilian form is at least 13 million years—and more probably some 25 million years—older than the new Argentinean titanosaur.

The craniodental anatomies of these sauropods differ dramatically. *Sarmientosaurus* retains a relatively plesiomorphic skull and wide-crowned dentition reminiscent of those of non-titanosaurian titanosauriforms such as *Abydosaurus* and *Giraffatitan*. *Tapuiasaurus*, by contrast, closely resembles other derived titanosaurians in having a proportionally low and elongate skull with narrow-crowned teeth restricted to the rostral ends of the jaws. The occurrence of *Sarmientosaurus* in the Cenomanian—Turonian suggests that, in South America and perhaps elsewhere, comparatively short-skulled, broad-toothed titanosaurs persisted alongside their more advanced, diplodocoid-like relatives for tens of millions of years, at least into the early stages of the Late Cretaceous. The disparate skull and tooth morphologies of these titanosaurs may well reflect distinct dietary preferences and/or feeding mechanisms; if so, the coexistence of these animals may have been facilitated by niche partitioning.

Another notable aspect of our strict consensus trees is that, in contrast to several earlier studies [12,14,180], *Rapetosaurus* and *Tapuiasaurus* are never recovered as members of Nemegtosauridae. Nemegtosauridae is defined as the stem-based clade that includes all titanosaurs more closely related to *Nemegtosaurus* than to *Saltasaurus*; apart from the former genus, the only other indisputable nemegtosaurid is *Quaesitosaurus* [11]. Intriguingly, until now, *Nemegtosaurus*, *Rapetosaurus*, and *Tapuiasaurus* were the only titanosaurians for which complete or nearly complete skulls had been described. We therefore suspect that the purported monophyly of these three genera has been an artifact of differential preservation among titanosaur taxa. Stated simply, *Nemegtosaurus*, *Rapetosaurus*, and *Tapuiasaurus* have been artificially ‘pulled’ together in previous analyses because they were the only titanosaurs for which most craniomandibular characters could be definitively scored. (D’Emic [115]:644–646] briefly discussed this phenomenon as it pertains to the phylogeny of Titanosauriformes more broadly, terming it the “monophyly of the preserved.”) The incorporation of *Sarmientosaurus* into phylogenetic analyses has had the effect of redistributing craniomandibular character data throughout Titanosauria, such that many putative nemegtosaurid synapomorphies are now proposed to characterize wider taxonomic groups. Notably, Wilson ([11]:313) predicted such an eventuality: “As more well-preserved titanosaur skulls are discovered, the distribution of characters supporting nemegtosaurid monophyly will likely broaden to diagnose more inclusive groups.”

Lastly, an unexpected result of our phylogenetic analyses involves the Early Cretaceous North American titanosauriform *Abydosaurus* [98]. This taxon has been nested within Brachiosauridae in all previous analyses in which it has been included [98,115,124,159,191]. In our strict consensus trees, however, *Abydosaurus* is always recovered as a basal titanosauriform that is closer to Titanosauria than to the only uncontroversial brachiosaurid in our matrices, *Giraffatitan*. This suggests that *Abydosaurus* may not be a member of Brachiosauridae, and that this clade (at least as it has been conceived by some recent authors [98,115,124]) may be paraphyletic. Intriguingly, *Abydosaurus* exhibits similarities with *Sarmientosaurus*: for instance, the orbit is proportionally large and the tooth count is closely comparable in both taxa. Most notably, *Abydosaurus* and *Sarmientosaurus* share a foramen on the lateral surface of the postorbital, near the junction of the rostrodorsal, caudodorsal, and ventral processes, a condition that is presently unique to these genera, but that may also occur in other sauropods (L.M.W., unpublished data). The significance of these morphologies should be investigated in future phylogenetic analyses of Titanosauriformes. Also, the comprehensive description of the *Abydosaurus* material (especially the abundant postcranial remains [98]) will undoubtedly clarify the systematic position of this taxon.

### Feeding Mechanism of *Sarmientosaurus*

The jaw mechanics of sauropods have been studied by numerous authors [140,143,192–200]. There is general consensus that, in these herbivorous dinosaurs, food was swallowed with little to no oral processing. Many works [192,201] have assumed the presence of a gastric mill that would have done much of the processing of ingested vegetation, although more recent studies [202] have cast considerable doubt on the existence of this type of structure in sauropods.

Calvo [140] argued that, in sauropods, dental microwear patterns provide evidence of the direction of jaw motion on the occlusal surfaces of the teeth, evidence that can, in turn, be evaluated by examining the mandibular joint. Based on his observations of titanosaur microwear and mandibular morphology, Calvo [140] proposed that these sauropods possessed a simple, up-down, orthal, isognathic jaw mechanism similar to that which he reconstructed for *Giraffatitan* but distinct from the propaliny he proposed for *Diplodocus*.

Christiansen [143] examined the feeding mechanics of *Giraffatitan*, among other sauropods. Although he could not precisely determine the cropping mechanism that this titanosauriform would have employed, he proposed that *Giraffatitan* ‘raked’ foliage from stems and branches in a fashion at least broadly comparable to that which he inferred for the diplodocoids *Dicraeosaurus* and *Diplodocus*. He also argued that the dental wear pattern of this brachiosaurid does not indicate that it was produced by tooth-to-tooth contact, despite the occasional existence of apical wear facets. Nevertheless, Christiansen [143] concurred with Calvo [140] in suggesting that *Giraffatitan* might also have utilized some form of shearing mechanism.

García and Cerda [61] proposed a hypothesis to explain the formation of wear facets on teeth that exhibit these facets on both their labial and lingual surfaces. These authors suggested that contact of the teeth with each other as well as with plant material produced these wear facets. García and Cerda [61] also argued that it is not possible to precisely reconstruct which plants a given sauropod might have eaten based solely on its dental microwear. This is because the animal would have frequently (and presumably, inadvertently) ingested sediment along with the vegetation it consumed, and this sediment would also have produced microwear on its teeth.

The premaxillary and maxillary teeth of *Sarmientosaurus* exhibit lingual and distal wear facets accompanied by slight, submesially-placed grooves. The functional dentary teeth do not have labial wear facets, but instead have strong distal wear facets. These distal wear facets on the dentary teeth are not present in the sample of 185 titanosaurian teeth from a diversity of geological formations analyzed by García and Cerda [61].

Calvo [140,145] considered the teeth of the sauropod ‘*Asiatosaurus mongoliensis*’ (widely regarded as a *nomen dubium*; [90,203,204]) to belong to Titanosauriformes due to the high angle of their wear. Nevertheless, the two teeth of this taxon described by Osborn [146] have mesiodistal wear facets that imply interdigitated occlusion as in *Camarasaurus* [150]. Calvo [140,145] regarded the jaw movements of ‘*Asiatosaurus*’ to be orthal, as in titanosauriforms. In *Sarmientosaurus*, the prominent distal wear facet of the dentary teeth—which is oriented parallel to the long axis of the tooth—is correlated with the submesial groove of the upper teeth. This demonstrates the existence of some degree of occlusal interdigitation, as present in the aforementioned macronarians but absent in advanced titanosaurians. Based on the subvertical wear facets of its teeth and the general orientation of its dental microwear [113], the new Patagonian taxon would have employed orthal jaw movements.

The peculiar orientation of the tooth crowns of *Sarmientosaurus* is not observed in other sauropods, and its functional significance is therefore difficult to interpret. However, the combination of subvertical premaxillary and mesial maxillary crowns and recumbent dentary crowns might indicate the existence of specialized shearing movements in the rostral part of the mouth that would have facilitated the slicing of plant matter.

### Diet of *Sarmientosaurus*

In interpreting the diet of *Sarmientosaurus*, several factors must be considered, including the type of teeth present in the new sauropod, the paleoenvironmental and paleobotanical record of the time and place in which the animal lived, and what is known about its body structure and posture. Ryan and Vickaryous [205] considered sauropods to be gut processors with simple teeth; assuming that this is the case, these dinosaurs must have had specialized intestines to digest fibrous, poorly nutritive plants. As tools for slicing vegetation, the teeth of *Sarmientosaurus* formed the first phase of the digestive process of this titanosaur.

When considering the diet of *Sarmientosaurus*, we must also take into account the probable habitual postures of the head and neck. Based on several lines of anatomical evidence (e.g., the likely orientation of the snout in life, the morphology of the inner ear and cervical series), Sereno et al. [123] envisioned the diplodocoid sauropods *Diplodocus* and *Nigersaurus* as low-height browsers when the neck was held in its neutral position. The inferred orientation of the occipital condyle of *Sarmientosaurus* indicates that, as in these diplodocoids, the skull was habitually held with the snout pointing mostly downward relative to the craniocaudal axis of the neck. The reconstructed anatomy of the semicircular canals supports this interpretation (see above) in that the ‘alert posture’ of the head in *Sarmientosaurus*—as determined by aligning the lateral semicircular canal with Earth horizontal (see Witmer et al. [118,132] and references therein)—also indicates a strongly downturned head posture. Furthermore, as in *Nigersaurus*, the new Patagonian titanosaur had an extensively pneumatized cervical series (see below). All of these features suggest a habitually neutral neck position that would have restricted the diet of *Sarmientosaurus* to relatively low-growing plants, as Stevens and Parrish [122] have proposed for other sauropods. Stevens and Parrish [206] suggested three possible forms of browsing with a habitually neutral neck posture: by ventriflexion, dorsiflexion, or neutral browsing, but all of these hypothesized positions are based primarily on digital analyses of the necks of sauropods for which this part of the skeleton is well preserved, such as *Apatosaurus* and *Diplodocus*. Unfortunately, the incompletely known cervical series of *Sarmientosaurus* precludes conducting these types of analyses on the new titanosaur. Similarly, we lack information on neck flexibility in *Sarmientosaurus*, and can only speculate on most of its relevant body dimensions (e.g., shoulder height). Stevens and Parrish [122,206] concluded that most sauropods were low- and medium-height browsers, but estimated that *Diplodocus*, for example, could feed up to four meters above the ground by virtue of its large body size and long neck. As did Sereno et al. [123] in the case of *Nigersaurus*, Parrish [207] argued that diplodocids and nemegtosaurid titanosaurs—with their downwardly-angled heads, rostrally broad snouts, and rostrally-located teeth—were adapted to browsing at low heights. Even though only the first of these features is present in *Sarmientosaurus*, it is, in our view, probable that the new titanosaur was primarily a low browser as well.

Given the comparatively robust nature of its teeth, it seems unlikely that *Sarmientosaurus* fed on soft vegetation, as has been proposed for *Nigersaurus* [123], but see [208]. Stevens and Parrish [122] argued that titanosaurs, especially derived members of the clade, used their teeth to strip leaves from stems or slice vegetation (albeit softer vegetation than more basal macronarians such as *Camarasaurus* and *Giraffatitan* could process).

The Cenomanian is widely considered as the interval when angiosperms began to dominate continental macrofloras, including those in Patagonia [209]; as such, it is likely that *Sarmientosaurus* regularly fed on some of these flowering plants. The oldest known records of Poales (grasses)—a potential food for the new titanosaur—are younger than the early Late Cretaceous [210], although it is possible that the group originated in Gondwana during the ‘middle’ Cretaceous [211].

The paleobotanical record of the Lower Member of the Bajo Barreal Formation (the unit that yielded *Sarmientosaurus*) includes fossil wood of the araucarian conifer morphotype *Agathoxylon* [212] and pollen of the angiosperm genera *Liliacidites*, *Nyssapollenites*, *Rousea*, *Tricolpites*, and *Verrutricolpites* [76,213].

An early Late Cretaceous angiosperm leaf assemblage was recovered some 900 km to the south of the *Sarmientosaurus* locality, in strata of the Cenomanian—Coniacian Mata Amarilla Formation of Santa Cruz Province [209]. This flora consists of a dozen morphotypes distributed in two depositional settings: a marine environment and a floodplain, the latter of which is comparable to the paleoenvironment reconstructed for the Lower Member of the Bajo Barreal Formation. Along with fern and conifer remains, these Mata Amarilla Formation strata have yielded fossil leaves consistent with the angiosperm genera *Laurophyllum*, *Sterculia*, *Myrcia*, and *Peumus*; the first two forms are suggestive of a subtropical, humid climate [214]. Given the geographic and stratigraphic proximity of the Bajo Barreal and Mata Amarilla formations, it is likely that *Sarmientosaurus* inhabited this type of paleoclimate as well. This is supported by the morphology of the *Agathoxylon* wood from the Bajo Barreal Formation, which has weak growth rings that indicate a lack of significant climatic seasonality [212]. In addition to angiosperms, plants such as gleicheniaceous ferns [215], horsetails, and cycads probably also contributed to the diet of the new titanosaur, as has been proposed for sauropods from other landmasses and temporal intervals [194,196,216–222].

### Cervical Pneumaticity

**Fig 32:**
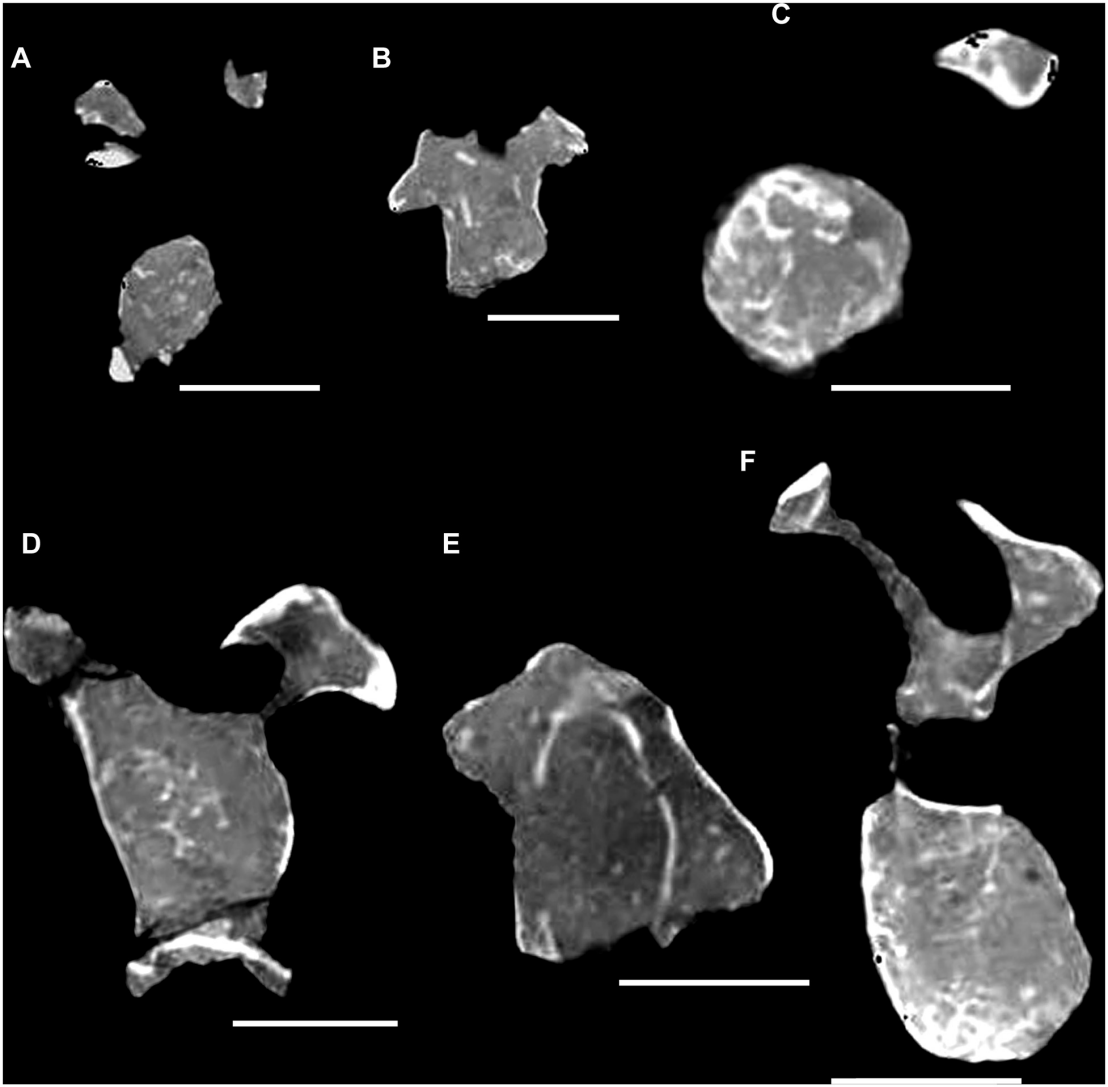
Computed tomographic transverse sections through cervical elements of *Sarmientosaurus musacchioi* gen. et sp. nov. (MDT-PV 2), showing extensive pneumaticity. (**A**) Axis and associated rib. (**B**) Cervical vertebra 3. (**C**, **D, E**) Cervical vertebra 6. (**F**) Cervical vertebra 7. Scale bars = 5 cm.

The cervical vertebrae of *Sarmientosaurus* show the camellate, highly pneumatic internal structure characteristic of the presacral vertebrae of somphospondylan sauropods (Fig 32). The existence of pneumaticity in sauropod vertebrae has been documented by numerous workers [32,155,223–229], but studies of axial pneumaticity in titanosaurs are relatively scarce [46,94,147,225,230,231]. Wedel [225] regarded the extensive vertebral pneumaticity of various sauropod clades (including Titanosauria) as a specialization related to increased body mass and neck length. Wedel [226] examined the extent of pneumaticity in different sauropods by calculating the Air Space Proportion (ASP) of transverse sections through vertebrae. He ([226]:table 7.2) estimated the average sauropod ASP at 50%–60% air by measured volume.

We examined the pneumaticity of the preserved vertebrae of *Sarmientosaurus* via a CT-based analysis, using the methodology proposed by Wedel [226]. To our knowledge, this analysis is the first of its kind yet performed on a titanosaurian cervical series. CT images revealed extraordinarily high ASP values—up to 88.19% air—in each axial cross section (see Table 6). The average ASP of the *Sarmientosaurus* axis and third cervical vertebra is 81.64%, whereas that of the sixth cervical vertebra is 70.56% and the seventh is 75.18%. Lower ASP values are generally observed in the articular condyles and cotyles of the centra; this is probably a consequence of biomechanical factors related to maintaining structural strength. By contrast, a cervical vertebra of a juvenile individual of the non-titanosaurian somphospondylan or basal titanosaurian *Phuwiangosaurus* has a mid-centrum ASP of only 55% [165,226]. Although ASPs vary considerably in non-titanosaurian sauropods, the maximum recorded values for cervical centra are 74% at mid-centrum in the titanosauriform *Sauroposeidon* and 77% near the caudal cotyle in the diplodocid *Tornieria* ([219]:table 7.2). Sereno et al. [123] reported extreme cervical vertebral pneumaticity—probably even greater than that of *Sarmientosaurus*—in the African rebbachisaurid *Nigersaurus*, but did not calculate an ASP or comparable quantitative value. Nevertheless, it is clear that the new Patagonian titanosaur possesses some of the most extensively pneumatized cervical centra yet documented within Sauropoda.

**Table 6:** Pneumaticity (Air Space Proportion, ASP) of cervical vertebrae of MDT-PV 2, the holotype of *Sarmientosaurus musacchioi* gen. et sp. nov. Percentages are based on computed tomographic slices and are listed from cranial to caudal.

Direction	Axis & Cervical 3	Cervical 6	Cervical 7
Cranial	80.51%	15.17% (condyle)	54.26%
	86.81%	59.21% (condyle)	66.49%
	88.19%	55.03% (condyle)	76.60%
	76.56%	80.72%	76.60%
	86.11%	75.94%	86.20%
	69.16%	83.04%	77.80%
	84.14%	81.72%	82.60%
		86.05%	83.63%
		84.54%	85.30%
Caudal		84.15%	62.33% (cotyle)
Average	81.64%	70.56%	75.18%

When considering the paleobiological significance of the highly pneumatic cervical vertebrae of *Sarmientosaurus*, we should also take into account the other distinctive morphologies of the taxon, such as exceptionally elongate cervical vertebrae accompanied by ossified, ventrolaterally-positioned tendons, a downwardly-angled skull, and a habitually neutral or proportionally low neck posture. This suite of features would have made the new titanosaur an efficient harvester of vegetation, probably from plants growing at medium to low heights. Interestingly, *Nigersaurus* also possesses the combination of an exceptionally pneumatized neck and a downward-facing snout [123]; nevertheless, the two forms differ considerably in that *Sarmientosaurus* has a much more robustly-constructed skull and teeth and (probably) a proportionally longer neck. These shared cranial and cervical features of *Nigersaurus* and *Sarmientosaurus* represent a series of previously undocumented convergences between Diplodocoidea and Titanosauria. Perhaps not coincidentally, these two sauropod genera inhabited mid-Cretaceous paleoecosystems in the western Gondwanan continents (Afro-Arabia and South America, respectively). This raises the intriguing possibility that the convergent morphologies observed in these taxa may represent an adaptive response to some factor common to these paleoenvironments, perhaps the diversification and increasing dominance of angiosperms during the mid-Cretaceous.

## Conclusions

**Fig 33:**
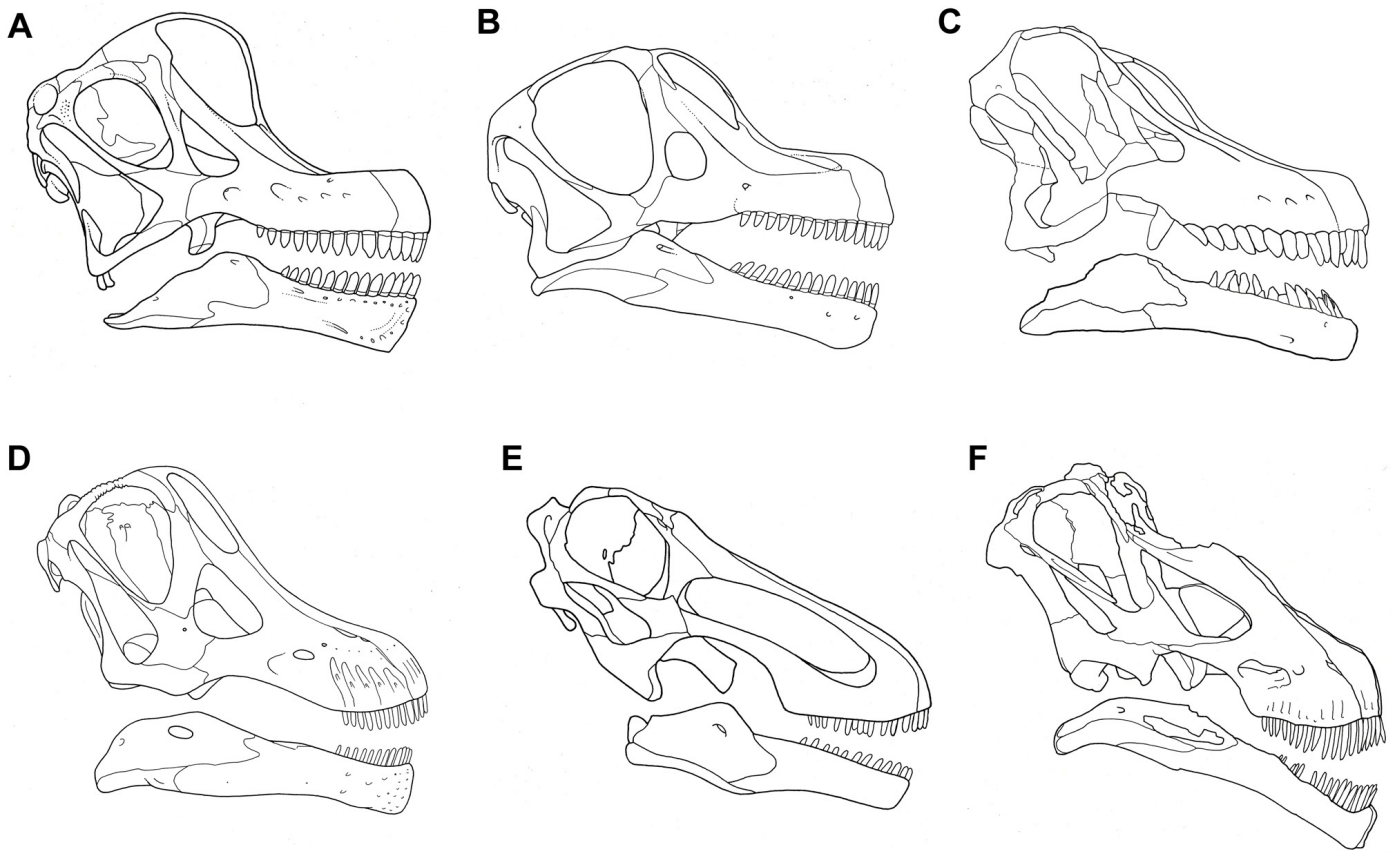
Comparison of titanosauriform sauropod dinosaur skulls in right lateral view. (**A**) *Giraffatitan brancai* (redrawn and modified from Wilson and Sereno [103]). (**B**) *Abydosaurus mcintoshi* (redrawn and modified from Chure et al. [98]). (**C**) *Sarmientosaurus musacchioi* gen. et sp. nov. (**D**) *Nemegtosaurus mongoliensis* (redrawn and modified from Wilson [11]). (**E**) *Rapetosaurus krausei* (redrawn from Curry Rogers and Forster [13]). (**F**) *Tapuiasaurus macedoi* (redrawn from Zaher et al. [14]). Not to scale.

**Fig 34:**
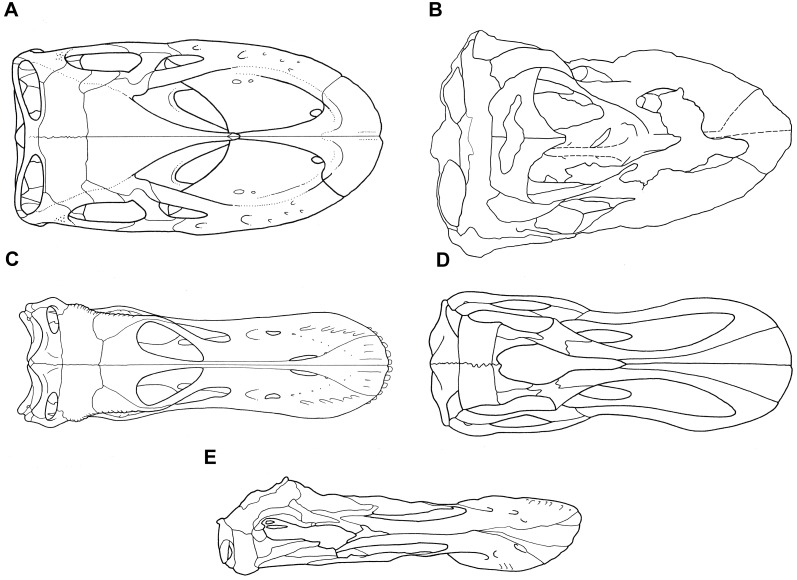
Comparison of titanosauriform sauropod dinosaur skulls in dorsal view. (**A**) *Giraffatitan brancai* (redrawn from Wilson and Sereno [103]). (**B**) *Sarmientosaurus musacchioi* gen. et sp. nov. (**C**) *Nemegtosaurus mongoliensis* (redrawn from Wilson [11]). (**D**) *Rapetosaurus krausei* (redrawn from Curry Rogers and Forster [13]). (**E**) *Tapuiasaurus macedoi* (redrawn from Zaher et al. [14]). Not to scale.

*Sarmientosaurus musacchioi* is the first titanosaurian sauropod from southern South America for which an articulated, virtually complete adult skull has been discovered. Phylogenetic analyses demonstrate that the new taxon is an archaic member of the titanosaurian subclade Lithostrotia, occupying a position more derived than *Malawisaurus* but more basal than taxa frequently regarded as nemegtosaurids (*Nemegtosaurus*, *Rapetosaurus*, and *Tapuiasaurus*) and saltasaurid titanosaurs such as *Alamosaurus*, *Neuquensaurus*, and *Saltasaurus*. As such, *Sarmientosaurus* is the most basal known titanosaur to be represented by a well-preserved skull. The new taxon exhibits a previously-undocumented cranial form that consists of an amalgam of plesiomorphic titanosauriform features such as a comparatively broad snout with a large narial fossa and a deep mandibular adductor chamber with more derived morphologies such as an elongate rostral process of the prefrontal (Figs 33 and 34). These characters offer novel cranial support for the phylogenetic hypothesis that titanosaurians are closely related to Brachiosauridae and other titanosauriforms—a hypothesis that, although now well-established, had previously been based primarily on evidence from the postcranial skeleton. Furthermore, the occurrence of the more derived lithostrotian *Tapuiasaurus* in the Aptian of Brazil raises the possibility that the new Patagonian taxon represents a titanosaurian ‘ghost lineage,’ the evolutionary history of which remains undocumented for almost all of the mid-Cretaceous.

*Sarmientosaurus* possesses a number of distinctive features that have not been observed in other sauropods. The cranial endocast presents among the most complete information about the brain and sensory systems for any sauropod, let alone titanosaurs, and thus will be important for the developing picture of neural evolution in Sauropoda. The endocast and inner ear possess clear titanosaurian apomorphies, but also retain a number of plesiomorphies, supporting the hypothesized phylogenetic position of *Sarmientosaurus* as a basal member of Lithostrotia. The dentition of the new Patagonian taxon is also unique in the disposition and morphology of its wear facets and the peculiar orientations of the upper and lower teeth. The adaptive significance of the latter feature is currently unclear. The presence of a ventrolaterally-positioned ossified tendon in the neck of the new sauropod is an anatomical novelty within non-avian dinosaurs. Ossified tendons occur in numerous ornithischian taxa, but in these animals, the tendons are associated with the neural spines of the caudal dorsal, sacral, and proximal caudal vertebrae. No other non-avian dinosaur exhibits ossified tendons situated ventrolateral to the cervical ribs, or tendons that maintain a constant, diminutive diameter over several meters. Moreover, *Sarmientosaurus* provides key data on the extent of cervical pneumatization in titanosaurs, in having vertebrae that are internally comprised much more by air than by bone. The new Argentinean lithostrotian exhibits remarkable convergence to the diplodocoid *Nigersaurus* in this regard.

Finally, the occurrence of *Sarmientosaurus* in the Cenomanian—Turonian Lower Member of the Bajo Barreal Formation underscores the significance of this geologic unit as an important source of information on non-avian dinosaur diversity and evolution during the early Late Cretaceous in southwestern Gondwana. Only 2 km from the *Sarmientosaurus* type locality, and from a stratigraphically equivalent horizon, earlier field efforts led by the senior author (R.D.F.M.) recovered a titanosaurian left maxilla [53] that differs dramatically from that of the new taxon. Intriguingly, this isolated maxilla is rostrocaudally short and dorsoventrally tall, suggesting that the sauropod to which it pertained had a similarly short, high skull, unlike the relatively elongate skull of *Sarmientosaurus*. Additionally, Powell et al. [51] reported a titanosaurian left premaxilla, again from Bajo Barreal Formation strata at least approximately correlative to those that produced *Sarmientosaurus*, but from a more distant locality (the Estancia Ocho Hermanos in the Sierra de San Bernardo). This premaxilla contrasts with that of the new lithostrotian in being relatively tall and mediolaterally wide. Collectively, these discoveries suggest that multiple titanosaurian lineages with disparate cranial morphologies coexisted in the Cenomanian—Turonian of central Patagonia. Perhaps these distinct cranial specializations reflect different dietary preferences in these large herbivores; if so, then these sauropods may have able to coexist by partitioning their niches to exploit different types of vegetation. Given that the angiosperm radiation was well underway by the early Late Cretaceous, these flowering plants were almost certainly a significant food source for *Sarmientosaurus* and other coeval titanosaurs. In sum, the new Patagonian sauropod provides critical insights into the cranial and cervical anatomy of basal members of Titanosauria, which in turn enhances our understanding of the evolutionary history and paleobiology of this extraordinarily diverse and abundant herbivorous dinosaur clade.

## Supporting Information

S1 Appendix Characters used in first (i.e., 337 character) phylogenetic analysis of *Sarmientosaurus musacchioi* gen. et sp. nov. (DOC)

S2 Appendix Data matrix for first iteration of first (i.e., 337 character) phylogenetic analysis of *Sarmientosaurus musacchioi* gen. et sp. nov. (22 ingroup taxa, 337 characters). (NEX)

S3 Appendix Data matrix for second iteration of first (i.e., 337 character) phylogenetic analysis of *Sarmientosaurus musacchioi* gen. et sp. nov. (24 ingroup taxa, 337 characters). (NEX)

S4 Appendix Data matrix for third iteration of first (i.e., 337 character) phylogenetic analysis of *Sarmientosaurus musacchioi* gen. et sp. nov. (25 ingroup taxa, 295 characters). (NEX)

S5 Appendix Data matrix for first iteration of second (i.e., based on Zaher et al. [14]) phylogenetic analysis of *Sarmientosaurus musacchioi* gen. et sp. nov. (31 ingroup taxa, 246 characters). (NEX)

S6 Appendix Data matrix for second iteration of second (i.e., based on Zaher et al. [14]) phylogenetic analysis of *Sarmientosaurus musacchioi* gen. et sp. nov. (22 ingroup taxa, 246 characters). (NEX)

S7 Appendix Data matrix for third iteration of second (i.e., based on Zaher et al. [14]) phylogenetic analysis of *Sarmientosaurus musacchioi* gen. et sp. nov. (23 ingroup taxa, 246 characters). (NEX)

S1 Fig Interactive, three-dimensional digital visualization of skull and reconstructed endocranial soft-tissues of *Sarmientosaurus musacchioi* gen. et sp. nov. generated from computed tomographic scan of the holotype (MDT-PV 2). **(Zaher et al. [14])** Color coding of reconstructed endocranial soft-tissues is as follows: endocast, lighter blue; endosseous inner ear labyrinth, pink; cranial nerves, yellow; arterial structures, red; venous structures, darker blue.
(PDF)

S1 Movie Rotating animation of digital visualization of cranium of *Sarmientosaurus musacchioi* gen. et sp. nov. generated from computed tomographic scan of the holotype (MDT-PV 2). Animation pauses in dorsal, right lateral, and ventral views; at these points, individual cranial bones and anatomical structures are labelled.
(MOV)

S2 Movie Animation of axial computed tomographic (CT) images of cranium of *Sarmientosaurus musacchioi* gen. et sp. nov. (MDT-PV 2). Positions of CT images indicated by orange line in digital visualization of cranium in right lateral view. Scale bar = 5 cm.
(MOV)

S3 Movie Animation of horizontal computed tomographic (CT) images of cranium of *Sarmientosaurus musacchioi* gen. et sp. nov. (MDT-PV 2). Positions of CT images indicated by orange line in digital visualization of cranium in right lateral view. Scale bar = 5 cm.
(MOV)

S4 Movie Animation of sagittal computed tomographic (CT) images of cranium of *Sarmientosaurus musacchioi* gen. et sp. nov. (MDT-PV 2). Positions of CT images indicated by orange line in digital visualization of cranium in dorsal view. Scale bar = 5 cm.
(MOV)

S5 Movie Animation of reconstructed endocranial soft-tissues of *Sarmientosaurus musacchioi* gen. et sp. nov. generated from computed tomographic scan data of the holotype (MDT-PV 2). Color coding is as follows: endocast, lighter blue; endosseous inner ear labyrinth, pink; cranial nerves, yellow; arterial structures, red; venous structures, darker blue. Abbreviations see text. Scale bar = 2 cm.
(MOV)

S6 Movie Animation of axial computed tomographic (CT) images of right (top) and left (bottom) mandibular rami of *Sarmientosaurus musacchioi* gen. et sp. nov. (MDT-PV 2). Positions of CT images indicated by orange line in digital visualizations of right and left mandibular rami in medial and lateral views, respectively. Scale bar = 5 cm.
(MOV)

S7 Movie Animation of horizontal computed tomographic (CT) images of right (top) and left (bottom) mandibular rami of *Sarmientosaurus musacchioi* gen. et sp. nov. (MDT-PV 2). Positions of CT images indicated by orange line in digital visualizations of right and left mandibular rami in medial and lateral views, respectively. Scale bar = 5 cm.
(MOV)

S8 Movie Animation of sagittal computed tomographic (CT) images of right (top) and left (bottom) mandibular rami of *Sarmientosaurus musacchioi* gen. et sp. nov. (MDT-PV 2). Positions of CT images indicated by orange line in digital visualizations of right and left mandibular rami in caudal view. Scale bar = 5 cm.
(MOV)
