# Supplementary material for: A Basal Lithostrotian Titanosaur (Dinosauria: Sauropoda) with a Complete Skull: Implications for the Evolution and Paleobiology of Titanosauria
Source: PLoS One. 2016 Apr 26;11(4):e0151661. doi: 10.1371/journal.pone.0151661 (PMC4846048; doi:10.1371/journal.pone.0151661)
Supplement: S1 Appendix — (DOC) [file pone.0151661.s001.doc]

**Appendix S1. Characters used in first (i.e., 337 character) phylogenetic analysis of *Sarmientosaurus musacchioi* gen. et sp. nov.**

Characters are ordered by anatomical region. Source abbreviations are as follows: **CARB**, Carballido et al. , Carballido and Sander ; **CPG**, Calvo et al. ; **CR**, Curry Rogers ; **CRF**, Curry Rogers and Forster ; **CS**, Calvo and Salgado ; **D**, D’Emic ; **G**, Gomani ; **GA**, Gallina and Apesteguía ; **GR**, González Riga ; **GROD**, González Riga and Ortiz David ; **GRPP**, González Riga et al. ; **H**, Harris ; **MUB**, Mannion et al. ; **P**, Powell ; **S**, Salgado ; **SCC**, Salgado et al. ; **SGP**, Salgado et al. ; **SLL**, Smith et al. ; **SZ**, Sanz et al. ; **U**, Upchurch ; **UBD**, Upchurch et al. ; **W**, Wilson ; **WS**, Wilson and Sereno . Numbers following source abbreviations correspond to character numbers in those analyses. * = character substantially modified from cited source.

**1.** **Morphology of rostral ends of jaws in dorsal view (U1*, U59*, WS31*, G2*, CRF39*, W65*, UBD1*, CR86*, H1, GA17*, CARB94*, GROD20*, SGP17*):**

0. triangular, with acute tip

1. broadly rounded rostrally with linear, roughly parallel lateral margins (U-shaped or parabolic)

2. platalean (broadly rounded and convex rostrally but with lateral margins that are sinuous, convex rostrally but becoming concave, producing spoon shape)

3. rectangular, especially lower jaw (rostral margin linear and at abrupt angle to lateral margins)

**2.** **Configuration of caudolateral processes of premaxilla and lateral processes of maxilla (U13*, WS105, G3*, CRF1, CRF177*, W1, UBD15*, CR2, H2, CARB1, D1, MUB75):**

0. lacking midline contact

1. possessing midline contact

**3.** **Angle between lateral and medial margins of premaxilla as seen in dorsal view (U12*, UBD12-14*, CR3*, H3*):**

0. >20º

1. between 10º-20º

2. <10º

**4.** **Morphology of rostral margin of premaxilla (= region rostral to nasal process) (U10, WS19, G1*, CRF174, W2, UBD10, H4, CARB2*, MUB1*, MUB76*):**

0. without 'step'

1. with marked 'step'; rostral margin of skull thus sharply demarcated (= muzzle-like area present)

**5.** **Free portion of nasal (= ascending) process of premaxilla in lateral view (U11*, CRF175*, UBD11*, CR4*, CR20*, H5, GRPP2*, GA9*, GROD12*, SGP9*):**

0. majority projects caudally and divides external nares into left and right halves

1. majority projects dorsally and divides external nares into left and right halves

2. is greatly reduced, reducing or eliminating internarial bar and creating single, confluent external narial opening

**6.** **Thin, plate-like process (flange) directed rostromedially from edge of maxillary ascending process (CS14*, U15-16*, CRF178*, UBD17-18*, CR8*, H6, MUB78*):**

0. absent

1. present but lacking midline contact

2. present and contacting each other at midline

**7.** **Direction in which subnarial foramen faces (U6, G5, CRF173, UBD6, CR14, H7):**

0. lateral

1. dorsal

**8.** **Proportions and size of subnarial foramen (U7, CRF3, UBD7, CR13, H8):**

0. small and subcircular

1. elongate (at least twice as long [in direction of premaxilla-maxilla suture] as wide)

**9.** **Position of subnarial foramen with respect to narial fossa (U8, G6, UBD8, H9):**

0. outside

1. within

**10.** **Relative positions of subnarial foramen and rostral maxillary foramen (W5, H10, CARB8):**

0. well distanced from one another

1. separated only by narrow bony isthmus

**11.** **External nares face (U4, CRF172, UBD4, CR17, H11):**

0. laterally or rostrolaterally

1. dorsally or rostrodorsally

**12.** **Shelf-like area or fossa (narial fossa) on premaxilla and maxilla lateral to external nares (U17*, CRF179*, W1*, UBD19, CR11*, H12):**

0. absent

1. present

**13.** **Length of border of external naris formed by maxilla (WS21*, G9, W3, H13, CARB5):**

0. short (less than 1/4 narial perimeter)

1. long (more than 1/3 narial perimeter)

**14.** **Position of midpoint of osteological external nares (CS7*, U2-3*, WS18*, S7*, G7-8*, CRF5*, CRF171*, W8*, UBD2-3*, CR16*, CR19*, H14, CARB12*):**

0. rostral to antorbital fenestra

1. dorsal to antorbital fenestra

2. caudal to antorbital fenestra

**15.** **Ratio of maximum diameter of osteological external nares:maximum diameter of orbit (U5*, WS89, CRF6, W9, UBD5, CR18, H15, CARB13, MUB2*):**

0. <1.0 (nares shorter)

1. >=1.0 (nares longer)

**16.** **Preantorbital fenestra (U18, WS74, S24, CRF2, W4, UBD20, CR12, H16, GRPP5, GA11, CARB7*, MUB79, GROD14, SGP11):**

0. absent

1. present

**17.** **Antorbital fossa (U19*, WS20, W7, UBD21, H17, CARB11):**

0. present

1. absent

**18.** **Ratio of maximum diameter of antorbital fenestra:maximum diameter of orbit (CRF4*, W6*, CR15*, H18*, CARB9*):**

0. <0.85 (orbit significantly larger)

1. 0.85-1.15 (diameters subequal)

2. >1.15 (antorbital fenestra significantly larger)

**19.** **Angle subtended by rostral and ventral margins of orbit (WS25*, W10, H19, CARB14):**

0. obtuse or roughly 90 degrees

1. markedly acute

**20.** **Rostral extent of maxillary (= rostral) process of lacrimal (U14*, W11*, UBD16*, H20, CARB15*, MUB80*):**

0. dorsal to midpoint of antorbital fenestra

1. caudodorsal to midpoint of antorbital fenestra but rostral to caudodorsal corner of antorbital fenestra

2. process absent; maxilla-lacrimal contact at caudodorsal corner of antorbital fenestra

**21.** **Element contacting ectopterygoid laterally (U54, WS76, CRF7, W12, UBD65, CR25, H21, CARB18):**

0. jugal

1. maxilla

**22.** **Contribution by jugal to antorbital fenestra (U20, CRF8, W13, UBD22, CR22, H22, CARB19):**

0. very reduced or absent

1. large (occupying most of caudoventral margin)

**23.** **Size of frontal (= caudal) process of prefrontal (CRF10, W14, CR31, H23, CARB27):**

0. small (does not project far beyond frontal-nasal suture)

1. elongate (approaches parietal)

**24.** **Morphology of frontal process of prefrontal in dorsal view (U28*, W15, UBD33, H24, CARB28, MUB82*):**

0. flat, broadly rounded or square

1. hooked or acute and subtriangular

**25.** **Morphology of jugal (= ventral) process of postorbital (WS75, W16, UBD28, H25, CARB31, MUB87, GROD4):**

0. mediolaterally narrow

1. broader mediolaterally than rostrocaudally

**26.** **Jugal (= ventral) process of postorbital (UBD31, H26):**

0. does not contact lacrimal (jugal intervenes)

1. contacts lacrimal (excludes jugal from margin of orbit)

**27.** **Squamosal (= caudal) process of postorbital (W17, H27, CARB32, GROD5):**

0. present

1. absent

**28.** **Frontal-parietal suture in dorsal view (WS65, CRF11*, W18, UBD34, CR34*, H28, CPG1*, GRPP6, GA2*, CARB34, GROD2, SGP2*):**

0. between supratemporal fenestrae/fossae (frontals contribute to rostral margin of fenestrae/fossae)

1. rostral to supratemporal fenestrae/fossae (frontals excluded from rostral margin of fenestrae/fossae)

**29.** **Midline contact (symphysis) between frontals in adults (CS13, U30, S31, CRF12, W19, UBD36, CR32, H29, CARB35):**

0. sutured

1. fused

**30.** **Ratio of rostrocaudal length:minimum mediolateral width of frontal (U29*, W20*, UBD35, H30, CARB36*):**

0. >=1.0 (equal or longer than wide)

1. <1.0 (wider than long)

**31.** **Dorsoventral height of occipital process of parietal (W21, H31, CPG2*, GRPP7*, GA4*, CARB40, MUB3*, GROD7*, SGP4*):**

0. short (less than diameter of foramen magnum)

1. deep (nearly twice the diameter of the foramen magnum)

**32.** **Contribution to posttemporal fenestra by parietal (CRF13, W22, UBD42, CR38, H32, CPG3*, GRPP9, GA7*, CARB41, MUB85, GROD10, SGP7*):**

0. present

1. absent

**33.** **Postparietal foramen (U34, S26*, CRF14, W23, UBD43, CR40, H33, CARB43):**

0. absent

1. present

**34.** **Morphology of infratemporal fenestra (CS9*, S6*, G18*, H34):**

0. subrectangular

1. subtriangular

2. linear (slit-like, crescentic)

**35.** **Position of rostralmost or rostroventralmost end of infratemporal fenestra (CS8*, U26-27*, WS25*, S32, G17*, CRF183*, W30*, UBD29-30*, CR44*, H35, MUB88*, CARB51*):**

0. caudal to orbit

1. rostral to caudal margin of but caudal to or equal with midpoint of orbit

2. rostral to midpoint of orbit

**36.** **Ratio of intraparietal distance separating supratemporal fenestrae:length of long axis of supratemporal fenestrae (CRF15*, W24*, CR39*, H36*, CPG4*, GRPP10*, GA8*, CARB42*, D5*, MUB4*, GROD11*, SGP8*):**

0. <1.0 (fenestra larger)

1. >=1.0 (intraparietal distance greater)

**37.** **Supratemporal fossa surrounds supratemporal fenestra (U31, UBD37, H37):**

0. present

1. absent

**38.** **Orientation of long axis of external supratemporal fenestra (WS66*, G16*, W25-26*, UBD40*, H38, CARB46*):**

0. rostrocaudal

1. mediolateral

2. dorsoventral (axis of fenestra appears longer in lateral than dorsal view)

**39.** **Contribution by squamosal to dorsal margin of supratemporal fenestra (U37, CRF185, UBD44, CR43, H39, CARB24*):**

0. present

1. absent (excluded by parietal-postorbital contact)

**40.** **Ratio of maximum diameter of supratemporal fenestra:diameter of foramen magnum (CS10*, U33*, S30*, G15*, CRF17*, W27, UBD41*, CR41*, H40, CARB47):**

0. >>1.0

1. ~1.0 (subequal)

**41.** **Ratio of rostrocaudal:mediolateral dimension of temporal bar (supratemporal region) (WS26, CRF18*, W28, H41, CARB48):**

0. >=1.0 (longer rostrocaudally)

1. <1.0 (longer mediolaterally)

**42.** **Lateral visibility of supratemporal fenestra (U32, WS27, G15*, CRF184, W29, UBD38-39*, CR42*, H42, CARB49, MUB86*):**

0. not visible (obscured by temporal bar)

1. visible (temporal bar shifted ventrally)

**43.** **Maxilla-quadratojugal contact (CS11*, U22, S2*, CRF180, UBD24, H43, CARB17*):**

0. absent

1. present

**44.** **Squamosal-quadratojugal contact (U35, WS23, CRF19*, W31, UBD45, CR48*, H44, CARB25, MUB89):**

0. present

1. absent

**45.** **Ratio of length of rostral (= jugal or maxillary) process of quadratojugal:length of dorsal (= squamosal) process (U21*, WS24*, W32*, UBD23*, H45, CARB21*, MUB5*):**

0. <=1.0

1. >=1.0

**46.** **Rostral process of quadratojugal (U23, UBD25, H46):**

0. tapers to acute tip

1. expands dorsoventrally at tip

**47.** **Orientation of rostral process of quadratojugal in lateral view (U24, CRF181, UBD26, CR46, H47, CARB23):**

0. straight or curves slightly dorsally at tip

1. angles ventrally at tip

**48.** **Angle between rostral and dorsal processes of the quadratojugal (U25*, CRF182*, UBD27, CR47*, H48, CARB22):**

0. roughly 90º

1. >>90º

**49.** **Orientation of long axis of quadrate with respect to long axis of skull (CS5, U56, S5, G20, CRF194, UBD68, CR52, H49):**

0. perpendicular

1. angled caudodorsally-rostroventrally

**50.** **Quadrate fossa (CS1*, U55*, WS28*, WS90*, G21-22*, CRF20*, W33-34*, UBD66-67*, CR51*, H50, CARB52-53*, MUB91*):**

0. absent

1. shallow

2. deeply invaginated

**51.** **Orientation of quadrate fossa (W35, H51, CARB54, MUB92):**

0. caudal

1. caudolateral

**52.** **Rostral articulation of vomer (W42, H52, CARB64, MUB95):**

0. with maxilla

1. with premaxilla

**53.** **Morphology of lateral ramus of palatine (WS29, CRF23, W40, CR83, H53, GRPP17, CARB62):**

0. plate-like (long maxillary contact)

1. rod-like (narrow maxillary contact)

**54.** **Rostral end of maxillary process of palatine (U46, UBD57, H54):**

0. poorly developed and unexpanded

1. mediolaterally expanded

**55.** **Composition of palatine (= rostral) ramus of pterygoid (U50, UBD61, H55):**

0. formed from two sheets of bone that project laterally and ventrally

1. formed from single, flat plate

**56.** **Ratio of width of main body of pterygoid:overall length of pterygoid (U51*, G23*, CRF192*, UBD62, CR77*, H56):**

0. <0.20

1. >=0.20

**57.** **Morphology of contact surface on pterygoid for basipterygoid articulation (U53-54*, WS96*, G24*, G26*, CRF21*, CRF193*, W36, UBD63-64*, CR78*, H57, CARB57, MUB93*):**

0. small facet

1. dorsomedially-oriented hook

2. rocker-like surface

**58.** **Rostrocaudal position of ectopterygoid process of pterygoid (U47-48*, CRF22, CRF190*, W37, UBD58-59*, CR81*, H58, CARB58):**

0. caudal or ventral to orbit

1. between orbit and antorbital fenestra or ventral to antorbital fenestra

2. rostral to antorbital fenestra

**59.** **Dorsoventral position of ectopterygoid process of pterygoid (U49, CRF191, UBD60, CR82, H59):**

0. projects below ventral margin of cranium (usually robust)

1. does not project below ventral margin of cranium (usually slender)

**60.** **Size of quadrate flange of pterygoid (W38, H60, CARB59):**

0. large (palatobasal and quadrate articulations well separated)

1. small (palatobasal and quadrate articulations approach)

**61.** **Shape of palatine ramus of pterygoid (W39, H61, CARB60):**

0. straight (at level of dorsal margin of quadrate ramus)

1. stepped (raised above level of quadrate ramus)

**62.** **Epipterygoid (W41, H62, CARB63):**

0. present

1. absent

**63.** **Ratio of dorsoventral height of supraoccipital:height of foramen magnum (W43*, H63, CPG5*, GRPP12*, GA12*, CARB65*, D6*, MUB6*, GROD15*, SGP12*):**

0. >=2

1. 1.01-1.99

2. <=1

**64.** **Sagittal and transverse nuchal crests merge smoothly at dorsal end of supraoccipital forming low tetrahedral process (S29, H64):**

0. absent

1. present

**65.** **Distal ends of paroccipital processes (U38*, CRF186*, UBD46, CR74*, H65, CARB44):**

0. flat or very slightly convex laterally

1. markedly convex laterally and expanded dorsally and ventrally ('tongue-like' process present)

**66.** **Ventral (nonarticular) process of paroccipital process (W44, H66, CPG6, GRPP13, GA13, CARB66, D8, MUB96, GROD16, SGP13):**

0. absent

1. present

**67.** **Morphology of occipital region of skull (CRF16, W54, CR57, H67, CARB80):**

0. concave caudally, with paroccipital processes oriented caudolaterally

1. flat with paroccipital processes oriented mediolaterally

**68.** **Orientation of occipital condyle (U39, S4, G27, CRF187, UBD47, CR58):**

0. caudoventral

1. ventral

**69.** **Size of crista prootica (U40, G14, CRF24, W45, UBD49, CR59, H68*, CARB67):**

0. rudimentary

1. laterally expanded into 'leaf-shaped' dorsolateral process

**70.** **Ratio of length:maximum basal diameter of basipterygoid processes (U42*, S27*, G12*, CRF25*, W46*, UBD52-53*, CR62*, H69, CARB68*, MUB9*, GROD18*):**

0. <=2

1. 2.01-3.99

2. >=4.0

**71.** **Angle of divergence between basipterygoid processes (U43*, S28, G13*, CRF26*, W47*, UBD54*, CR64*, H70, GRPP15*, GA15*, CARB69*, SGP15*):**

0. >=30°

1. <30°

**72.** **Orientation of basipterygoid processes (CS6, U41, S3, G10, CRF29*, W53*, UBD50, CR63*, H76, CARB78*)):**

0. roughly perpendicular to skull roof (ventrolateral)

1. forming markedly acute angle to skull roof (rostral or rostrolateral)

**73.** **Cross-sectional morphology of basipterygoid processes (UBD51, H77, MUB101):**

0. elliptical or subtriangular

1. subcircular

**74.** **Ratio of rostrocaudal depth:dorsoventral height of basal tubera (CRF27*, W48*, UBD48*, CR70*, H71, GRPP16*, GA16*, CARB70*, GROD19*, SGP16*):**

0. >=0.25

1. <0.25 (sheet-like)

**75.** **Ratio of transverse width of paired basal tubera:transverse width of occipital condyle (W49*, H72, CARB71*, D7*, MUB8*):**

0. <1.0

1. 1.0-1.2

2. >1.2

**76.** **Basisphenoid fossa/foramen between foramen magnum and basal tubera (CRF28, W50, CR68, H73, CARB74):**

0. absent

1. present

**77.** **Region between basipterygoid processes (U44, CRF188, W51*, UBD55, CR65, H74, CARB75*):**

0. shallowly concave

1. deep pit

**78.** **Basisphenoid-quadrate contact (W52, H75, CARB76, M100):**

0. absent

1. present

**79.** **Morphology of parasphenoid rostrum (U45, CRF189, UBD56, CR69, H78):**

0. broadly triangular in lateral view and with groove on dorsal margin

1. slender, spike-like, and lacking dorsal groove

**80.** **Rostral end of dentary ramus (U57, WS30*, G30*, CRF30*, W55*, UBD69, CR84*, H79, CARB81*):**

0. decreases in or maintains dorsoventral height rostrally

1. increases in dorsoventral height and robustness rostrally

**81.** **Morphology of rostroventral margin of dentary (U58, CRF31, W56, UBD70, CR88, H80, CARB82):**

0. gently rounded

1. sharply projecting triangular process ('chin')

**82.** **Angle between dentary symphysis and long axis of jaw ramus (U60*, CRF195*, W57*, UBD71*, CR90*, H81, CARB83*):**

0. <75°

1. >75° (close to perpendicular)

**83.** **Ratio of length of external mandibular fenestra:length of mandible (U62-63*, WS77*, G29*, CRF33*, W58*, UBD76-77*, H82, CARB87*, MUB102*):**

0. >=0.10

1. <0.10

2. absent

**84.** **Ratio of surangular dorsoventral depth:maximum depth of angular (WS91*, CRF34*, W59*, UBD75, CR91*, H83, CARB88*, D11*, MUB10*):**

0. <2.0

1. >=2.0

**85.** **Ridge on surangular separating adductor and articular fossae (W60, H84, CARB89):**

0. absent

1. present

**86.** **Depth of medial wall of adductor fossa (CRF35*, W61, H85, CARB90):**

0. shallow

1. deep, with prearticular expanded dorsoventrally

**87.** **Position of rostral end of splenial relative to mandibular symphysis (U61, UBD72, H86):**

0. caudal to symphysis

1. participates in symphysis

**88.** **Position of caudal process of splenial (CRF36, W62, UBD74, CR97, H87, CARB91):**

0. overlaps angular medially

1. intervenes between rostral portions of prearticular and angular

**89.** **Caudodorsal process of splenial (CRF37, W63, UBD73, CR98, H88, CARB92):**

0. present, approaching margin of adductor chamber

1. absent

**90.** **Size of coronoid (W64, H89, CARB93):**

0. extends to dorsal margin of jaw

1. reduced, does not extend to splenial

2. absent

**91.** **Plate of bone lying lateral to teeth on premaxilla, maxilla, and dentary (U9, UBD9, H90, CARB98):**

0. absent

1. present

**92.** **Position in tooth row of largest teeth (U65*, UBD78, H91):**

0. in maxilla, at midlength

1. rostral end of jaws

**93.** **Number of dentary teeth (WS67*, G31*, CRF45*, W73*, UBD91, CR100, H92, CARB96*, D12*, MUB13*):**

0. >=18

1. <=17

**94.** **Length of tooth rows (U73-74*, G28*, CRF40*, W66, UBD94-95*, H93, CARB95*, MUB104*):**

0. extends to orbit

1. rostral to orbit but caudal to subnarial foramen

2. restricted rostral to subnarial foramen

**95.** **Occlusal pattern (WS35-36*, G35*, CRF41*, W67-68*, CR103-104*, H94, CPG9*, GRPP20*, GA20*, CARB101-102*, MUB105*, GROD23*, SGP20*):**

0. absent

1. interlocking (creating V-shaped wear facets)

2. planar facets at markedly acute angle to long axis of tooth (high-angled)

3. planar facets at roughly 90° angle to long axis of tooth (low-angled)

**96.** **Orientation of tooth crowns (WS34, CRF42*, W69, UBD80, CR102*, H95, CARB100, D13*, MUB106*):**

0. aligned along jaw axis (crowns do not overlap)

1. aligned slightly mesiolingually (tooth crowns overlap)

**97.** **Contact between adjacent tooth crowns (UBD81, H96):**

0. present

1. absent

**98.** **Ratio of apicobasal length of tooth crown:mesiodistal width of lingual face (= 'slenderness index') (U69-71*, SLL1*, CRF197*, UBD87-89*, CR104*, CR108*, H97, CPG8*, GRPP19*, GA18*, CARB108*, D16*, MUB11*, MUB108*, GROD21*, SGP18*):**

0. <=3.0 (crowns very expanded; teeth spatulate)

1. 3.01-3.99

2. >=4.0

**99.** **Cross-sectional shape of tooth crowns at mid-crown (CS2*, U67*, WS32, S1*, SZ1*, G32*, SLL2*, CRF43, W70, P1*, UBD84-85*, UBD92*, CR105, H98, CPG10*, GRPP21*, GA19*, CARB103*, MUB109-110*, GROD22*, SGP19*):**

0. elliptical (convex both labially and lingually)

1. D-shaped (convex labially, flat or concave lingually)

2. circular (= 'pencil-' or 'peg-like' tooth morphology)

**100.** **Enamel surface texture (WS33, G33, W71, UBD79, H99, CARB104):**

0. smooth

1. wrinkled

**101.** **Marginal tooth denticles (CS3*, U66*, WS78*, G34, CRF44*, W72, UBD82-83*, CR106*, H100, CARB106, D14*, MUB113*):**

0. present

1. absent on distal margin only

2. absent on both mesial and distal margins

**102.** **Number of replacement teeth per alveolus (CRF46*, W74*, UBD90*, CR109*, H101, CARB97*):**

0. <=2

1. >=3

**103.** **Orientation of teeth (U72*, CRF47*, W75*, UBD93*, CR101*, H102*, CARB99*):**

0. perpendicular to jaw margin

1. oriented rostrally with respect to jaw margin (procumbent)

2. oriented rostrally in upper jaw (procumbent) and caudally in lower jaw (recumbent)

**104.** **Longitudinal grooves on lingual face of tooth (W76, H103, CARB107):**

0. absent

1. present

**105.** **Prominent grooves near mesial and distal margins of labial surface of tooth crowns (U68, CRF196*, UBD86, CR107*, H104):**

0. absent

1. present

**106.** **Semicamellate or camellate (= 'cancellous,' 'somphospondylous') osseous tissue structure in presacral vertebrae (U205, WS102, SZ16*, G47, SLL3, CRF48, W77, GR12, P15*, UBD309, CR110, CPG30, GRPP23, GA41, CARB120*, CARB139*, D18*, MUB115*, MUB141*, GROD44, :**

0. absent

1. present

**107.** **Number of cervical vertebrae (U75-79*, WS37*, S25*, G36*, CRF51*, W80*, UBD96-100*, CR113*, H105, CPG11*, GRPP24*, GA21*, CARB109*, D19*, MUB14*, GROD25*, SGP21*):**

0. <=9

1. 10

2. 11

3. 12

4. 13

5. 14

6. >=15

**108.** **Shape of occipital facet of atlantal intercentrum (CRF50, W79, UBD101*, CR112, H106, CARB110, MUB116):**

0. rectangular in lateral view (dorsal and ventral lengths subequal)

1. wedge-shaped (craniocaudal length of ventral margin greater than that of dorsal margin)

**109.** **Morphology of articular facets of cervical centra (SCC1*, U81, WS38, G49, W82, UBD103, H107, CARB111):**

0. amphicoelous/amphiplatyan

1. opisthocoelous

**110.** **Morphology of cervical lateral pneumatic fossae ('pleurocoels') (CS15*, SCC8*, U87*, WS69*, S33*, G50-51*, CRF53*, W78*, W83*, GR9*, UBD110*, CR120*, CR133*, H108, CPG12-13*, GRPP25-26*, GA22-23*, CARB115*, D17*, D21*, MUB122*, SGP22*):**

0. absent

1. simple, undivided

2. simple and undivided in cranial cervicals but becoming complex (divided by bony septa) in caudal cervicals

3. complex in all cervicals

**111.** **Morphology of ventral surface of cervical centra (U83-84*, G40-41*, CRF198-199*, UBD106-107*, CR118-119*, CR132*, H109, CARB112-113*, MUB117-120*):**

0. with prominent sagittal keels

1. flat or mildly convex ventrally

2. concave ventrally

**112.** **Fossae on dorsal surface of parapophyses of cervical vertebrae (U86, CRF201, UBD109, CR115*, H110, MUB121*):**

0. absent

1. present and confluent with lateral pneumatic fossa ('pleurocoel')

2. present but separated from lateral pneumatic fossa ('pleurocoel') by ridge

**113.** **Lamination (especially centrodiapophyseal) of cervical neural arches (U90*, WS101, G52, CRF52, W81, UBD115-116*, CR114, CR137*, H111, CARB118):**

0. well developed, with well defined laminae and fossae

1. rudimentary; diapophyseal laminae only feebly developed or absent

**114.** **Bifurcation of cervical neural spines (CS17*, U92*, WS106*, S13*, S34*, CRF54*, CRF57*, W85*, UBD118*, CR123*, CR141*, H119, CPG17*, GRPP28*, GA27*, GRPP31*, CARB122*, D22*, MUB132*, GROD30*, SGP26*):**

0. absent

1. present only on caudal cervicals

2. present on middle cervicals (to C6)

3. present on cranial cervicals (cranial to C6)

**115.** **Neural spine height in cervical vertebrae (U91, G37*, G44*, UBD117, H118, CPG16*, CPG18*, GRPP29*, GRPP32*, GA26*, GA28*, D30*, MUB19*, GROD29*, GROD31*, SGP25*, SGP27*):**

0. low (dorsoventral height of vertebra less than or subequal to craniocaudal centrum length)

1. high (developed into craniocaudally abbreviate, dorsoventrally elongate processes)

**116.** **Ratio of caudal articular surface height:width of cranial cervical centra (U85*, G42*, CRF200*, W84*, UBD108, CR116*, H112, CARB116*, MUB16*):**

0. <1.25

1. >=1.25

**117.** **Orientation of cranial cervical neural spines (CS18, WS103*, G48*, CR125*, H113):**

0. dorsal or craniodorsal

1. caudodorsal

**118.** **Ratio of craniocaudal centrum length:dorsoventral height of caudal face of middle cervical centra (U80*, WS107*, G46*, CRF55*, W86*, UBD102, CR130*, H114, CARB126, D23*, MUB15*):**

0. <4.0

1. >=4.0

**119.** **Ratio of dorsoventral height of middle cervical neural arches:dorsoventral height of caudal articular facet of centrum (CS16*, WS39*, G53, CRF56, W87, UBD111-112*, H115, CARB125):**

0. <1.0

1. >=1.0

**120.** **Morphology of centroprezygapophyseal lamina on middle and caudal cervical neural arches (U88*, G43*, CRF202*, W88*, UBD113*, CR129*, H116, CARB127, MUB130*):**

0. single

1. divided (= cranial infrazygapophyseal fossae present)

2. consists of two parallel laminae

**121.** **Morphology of articular surfaces of prezygapophyses on middle and caudal cervical neural arches (U89, UBD114, H117, CARB129):**

0. flat

1. transversely convex

**122.** **Orientation of caudal margin of caudal cervical neural spines (U93, UBD119, H121, CARB130*):**

0. nearly vertical

1. slopes craniodorsally-caudoventrally

**123.** **Morphology of caudal cervical and cranial dorsal neural spines (CS17*, CS19*, U92*, WS106*, S13-14*, G55*, CRF58*, W89-90*, GR3*, UBD118, CR141-142*, CR150*, H120, CPG22*, GRPP36*, GA32*, CARB132-133*, D32, MUB132-133*, MUB138*, GROD35*, SGP31*):**

0. single

1. bifid but lacking sagittal tubercle

2. bifid with sagittal tubercle ('trifid')

**124.** **Number of dorsal vertebrae (U95*, WS70*, G56*, CRF59*, W91, UBD122-125*, CR143*, H122, CPG21*, GRPP35*, GA31*, CARB134*, MUB20*, GROD34*, SGP30*):**

0. 15

1. 14

2. 13

3. 12

4. 11

5. <=10

**125.** **Lateral pneumatic fossae ('pleurocoels') in majority of dorsal centra (CS22*, SCC8*, U97-98*, SZ4*, G60*, CRF203-204*, W78*, GR9*, P4*, UBD128-129*, CR111*, CR146-147*, H123, CARB135*, D17*, MUB143-144*):**

0. absent

1. present as deep but simple pits

2. present as deep excavations that ramify into centrum and base of neural arch (leaving only thin midline septum)

**126.** **Lateral position of pneumatic fossae ('pleurocoels') on dorsal centra (UBD130, H124*, MUB145):**

0. flush with lateral surface (no 'pleurocoelous fossa')

1. set within 'pleurocoelous fossa'

**127.** **Ratio of dorsoventral height of dorsal neural arch:dorsoventral height of corresponding dorsal centrum (CS20*, CS24*, CS28*, U100*, S9*, SZ13*, CRF203-204*, W93*, P13*, UBD132*, H137, D30*):**

0. <=1.0

1. >1.0

**128.** **Pneumatic cavity within some or all dorsal neural arches (U106, UBD141, CR172*, H147):**

0. absent

1. present but not open externally

2. present and open externally via foramen immediately ventral to the diapophysis

**129.** **Cranial face of dorsal neural arches (U101, UBD136, H125, CARB155*):**

0. flat or shallowly excavated

1. deeply excavated

**130.** **Hyposphene-hypantrum articulations on dorsal neural arches (CS23*, SCC25*, U109*, S15*, SZ6*, G64*, SLL10*, CRF71*, W106*, P6*, GR8*, UBD145*, CR162*, CR170*, H126, CPG28*, GRPP46*, GA39*, CARB151-152*, D48*, MUB149*, GROD42*, SGP38*):**

0. absent

1. present on middle and/or caudal dorsals only

2. present on cranial-caudal dorsals

**131.** **Single midline hyposphenal lamina on dorsal neural arches (U110, UBD146, CR163*, CR191*, H127, CARB152*, CARB154*):**

0. absent

1. present

**132.** **Infradiapophyseal excavation on dorsal neural arches (U108, CRF68, W103, UBD144, H145):**

0. absent

1. present

**133.** **Orientation of diapophyses on dorsal neural arches (U102, S35, G67, CRF206*, UBD138, CR154-155*, CR168*, H131, CARB136, D41*, MUB153*, MUB155*):**

0. lateral or slightly dorsal

1. strongly dorsolateral (approximately 45º to horizontal)

**134.** **Dorsal neural arches with spinodiapophyseal lamina (U118*, SZ9*, W99*, P9*, UBD156-157*, H128, CARB164*, MUB166*):**

0. none

1. on caudal dorsals only

2. on middle and caudal dorsals

**135.** **Accessory spinodiapophyseal lamina on dorsal neural arches (SCC30*, SZ12*, SLL14*, P12*, UBD151, H129, CARB165*, D31*, MUB136*):**

0. absent

1. present

**136.** **Postspinal lamina on dorsal neural arches (CS27*, U114*, G72*, SLL6*, CRF209*, UBD149, CR161*, CR183*, CR186*, H130, GRPP41*, MUB169*):**

0. absent

1. present

**137.** **Triangular (= aliform) processes projecting laterally from distal ends of dorsal neural spines (U116*, WS86*, SZ10*, G59*, CRF67*, CRF211*, W102*, P10*, UBD153-154*, CR171*, CR177*, H148*, GRPP39*, CARB163*, MUB162-163*):**

0. absent

1. present but do not project far laterally (not as far as postzygapophyses)

2. present and project very far laterally (as far as postzygapophyses)

**138.** **Morphology of articular face of cranial dorsal centra (WS59, G57, W94, UBD104, CR145*, H132*, CARB146, D38):**

0. amphicoelous/amphiplatyan

1. opisthocoelous

**139.** **Morphology of caudal margins of lateral pneumatic fossae ('pleurocoels') on cranial dorsal centra (CS29*, SCC20*, U96, SZ11*, SLL4*, GR10*, P11*, UBD127, H133, CPG29*, GRPP47*, GA40*, CARB147*, MUB146, GROD43*, SGP39*):**

0. rounded

1. acute

**140.** **Morphology of ventral surfaces of cranial dorsal centra (SZ3*, P3*, UBD126*, H134, MUB142*):**

0. ventrally convex

1. flat

2. with sagittal crest (creating two ventrolaterally-facing surfaces)

3. ventrally concave with sagittal crest in resultant sulcus

**141.** **Centroprezygapophyseal lamina on cranial dorsal neural arches (SZ2*, CRF61*, P2*, UBD134*, CR157*, H135*):**

0. consists of single lamina

1. bifurcate toward dorsal end

2. consists of two subparallel laminae

3. absent

**142.** **Orientation of neural spines of cranial dorsal neural arches (GR4*, UBD158, CR151*, H136, CPG23*, GRPP37*, GA34*, MUB160*, GROD37*, SGP33*):**

0. dorsal or caudodorsal

1. craniodorsal

**143.** **Morphology of centroprezygapophyseal lamina on middle and caudal dorsal neural arches (UBD135, H138):**

0. single

1. divided dorsally

**144.** **Centropostzygapophyseal lamina on middle and caudal dorsal neural arches (W95, CARB158):**

0. single

1. divided

**145.** **Cranial (= anterior) centroparapophyseal lamina on middle and caudal dorsal neural arches (CRF63*, W96, UBD133, CR178*, H139, GA37*, CARB159, SGP36*):**

0. absent

1. present

**146.** **Prezygoparapophyseal lamina on middle and caudal dorsal neural arches (CRF64*, W97, CR179*, H140, CARB160):**

0. absent

1. present

**147.** **Caudal (= posterior) centroparapophyseal lamina on middle and caudal dorsal neural arches (SCC22, U105, G63, SLL12, CRF65, W98, GR6*, UBD137, CR180, H141, CPG26*, GRPP44*, GA37*, CARB161, MUB148*, GROD40*, SGP36*):**

0. absent

1. present

**148.** **Lamination on cranial face of neural spine of middle and caudal dorsal neural arches (CS26*, SCC14*, U113*, SZ15*, G71*, SLL5*, CRF208*, GR5*, P14*, UBD148*, CR182*, CR185*, H143, CPG25*, GRPP40*, GA36*, CARB140-142*, D45*, MUB168*, GROD39*, SGP35*):**

0. none

1. prespinal lamina present, spinoprezygapophyseal laminae absent or indistinct

2. prespinal lamina absent, spinoprezygapophyseal laminae present

3. both prespinal and spinoprezygapophyseal laminae present and connected to each other either directly (merging) or via accessory laminae

4. both prespinal and spinoprezygapophyseal laminae present but unconnected to each other

**149.** **Spinopostzygapophyseal laminae on middle and caudal dorsal neural arches (U112*, CRF66*, W100*, UBD147*, UBD150*, CR181*, H144*, GRPP43*, CARB168*, D37*, MUB165*):**

0. absent

1. present and single throughout length

2. present and ventrally bifurcate

**150.** **Accessory lamina in the infrapostzygapophyseal cavity of middle and caudal dorsal neural arches (U107, G66, UBD143):**

0. absent

1. present

**151.** **Spinodiapophyseal-spinopostzygapophyseal lamina contact on middle and caudal dorsal neural arches (WS60, G58, W101, H146, CARB169):**

0. absent

1. present

**152.** **Orientation of middle and caudal dorsal neural spines (CRF69, W104, CR169*, H149*, CARB157*, D46*, MUB164*):**

0. vertical or craniodorsal

1. caudodorsal (distal end approaches level of diapophyses)

**153.** **Morphology of articular face of caudal dorsal centra (CS25, SCC9*, U82*, WS92, SZ7*, G73, CRF70, W105*, GR2, P7*, UBD105, CR166, H150, CARB174*, D39, MUB147*):**

0. amphicoelous/amphiplatyan

1. opisthocoelous

**154.** **Cross-sectional morphology of caudal dorsal centra (UBD131, CR144*, H151, CARB162*, MUB22*):**

0. subcircular

1. dorsoventrally compressed

**155.** **Ventral end of posterior centrodiapophyseal lamina of caudal dorsal neural arches (SCC21*, G62, SLL11*, GR7, UBD142, CR193*, H152, CPG27, GRPP45, GA38, CARB176, D43*, MUB151*, GROD41, SGP37):**

0. unexpanded

1. expands and may bifurcate

**156.** **Position of diapophysis on caudal dorsal neural arches (U103, SZ14, G68, SLL8, CRF207, UBD139, CR175, H153, CARB173, MUB157):**

0. caudal or caudodorsal to parapophysis

1. dorsal to parapophysis

**157.** **Distal end of the diapopophysis in caudal dorsal neural arches (SZ5, SLL9, P5, UBD140, H142*, CARB137*, D47*, MUB156*):**

0. grades smoothly into the dorsal surface of the diapophysis

1. is set off from the dorsal surface, the latter having a distinct dorsally facing flattened area

**158.** **Ratio of transverse width:craniocaudal length of caudal dorsal neural spines (U111*, U115, WS 40*, CRF210, W92*, UBD152, CR176, H154, CARB175, D44*):**

0. <=1.0 (longer than wide)

1. >1.0 (wider than long)

**159.** **Morphology of caudal dorsal neural spines in cranial view (U117*, CRF72, W107, UBD155, CR174, H155, CARB138*):**

0. rectangular for most of its length with little or no lateral expansion (except at distal end)

1. progressively expanding transversely through most or all of its length ('petal' or 'paddle' shaped)

**160.** **Ratio of dorsoventral height of neural spine:dorsoventral height of caudal articular facet of centrum in caudal dorsal, sacral and cranial caudal vertebrae (CS33*, U125-126*, S36*, G70*, G77*, GR11*, UBD166-167*, CR153*, CR167*, CR199*, CR216*, H164, CPG24:**

0. <2.0

1. 2.0-3.0

2. >3.0

**161.** **Number of sacral vertebrae (SCC2*, U120-122*, WS2*, G76, SLL15*, CRF73*, W108, GR13*, UBD161-163*, CR194*, H156*, CPG31*, GRPP48*, GA42*, CARB181*, D50*, MUB24*, GROD45*, SGP41*):**

0. <=3

1. 4

2. 5

3. 6

4. 7

**162.** **Sacricostal yoke (WS61, G78, W109, H157, CARB182, D51):**

0. absent

1. present

**163.** **Participation of the sacricostal yoke in the dorsal margin of the acetabulum (UBD169):**

0. absent

1. present

**164.** **Ratio of maximum transverse width across sacral vertebrae and ribs:average length of sacral centrum (U123*, UBD164, H158):**

0. <4.0

1. >=4.0

**165.** **Lateral pneumatic fossae ('pleurocoels') and/or foramina in sacral centra (U124, SLL16, CRF213, UBD165, CR198, H159, CARB186, MUB173):**

0. absent

1. present

**166.** **Ratio of length of sacral neural spines:craniocaudal length of centrum (CRF75*, W111*, CR199*, H160, CARB184*):**

0. <2.0

1. 2.0-3.49

2. >=3.50

**167.** **Dorsoventral height of sacral ribs (U127, CRF76*, W112, UBD168, CR205*, H161, CARB185):**

0. low (not projecting beyond dorsal margin of ilium)

1. high (extending to or beyond dorsal margin of ilium)

**168.** **Caudal vertebral internal tissue structure (SZ16*, SLL17*, CRF77, W113, P15*, CR206, H162, GRPP50, CARB188, MUB176*):**

0. solid

1. camellate/'somphospondylous' (with large internal cells)

**169.** **Number of caudal vertebrae (U128*, CRF78*, W114*, UBD170*, CR207*, H163, GRPP49*, CARB187*, D53*, MUB175*):**

0. <=35

1. 36-60

2. >=61

**170.** **Caudal vertebral transverse processes (ribs) (U144*, CRF79*, W115, UBD193*, CR208*, H165, CPG34*, GRPP71*, GA45*, CARB189, MUB201*, GROD48*, SGP44*):**

0. persist through caudal vertebra 20 or further distally

1. disappear by caudal 15

2. disappear by caudal 10

**171.** **Length of caudal centra (U133, CRF84, W120, UBD178, CR213, H171, CARB204):**

0. ~same over first 20

1. doubling over first 20

**172.** **Morphology of articular face of first caudal centrum (CS30*, SCC32*, G79*, SLL20-21*, CRF80, W116, UBD171*, CR209, H166, CPG32, GRPP51, GA43, CARB190*, GROD46, SGP42):**

0. flat (amphiplatyan)

1. procoelous

2. opisthocoelous

3. biconvex

**173.** **Coel or fossa on lateral aspect of first caudal neural arch (CRF81, W117, CR210, H167*, CARB191):**

0. absent

1. present

**174.** **Morphology of articular face of cranial caudal centra (excluding first) (CS30*, SCC23*, U129-130*, S16*, SZ18*, G80, SLL20-21*, CRF82*, W118*, GR16*, P17*, UBD173-174*, CR211*, H168, CPG37*, GRPP52*, GA48*, CARB193*, D55*, MUB27*, MUB177*, GROD51*, SGP47*):**

0. amphiplatyan or platycoelous

1. weakly procoelous

2. strongly procoelous

3. opisthocoelous

**175.** **Morphology of articular surfaces in cranial caudal centra (SCC38*, SLL19*, GR14*, UBD172, H169, CPG35*, GRPP55*, GA46*, MUB25*, GROD49*, SGP45*):**

0. subcircular

1. dorsoventrally compressed

2. transversely compressed

**176.** **Lateral pneumatic fossae ('pleurocoels') or deep depressions on cranial caudal centra (CS31, U135, CRF83, W119, UBD181, CR212, H170, CARB194, D56*, MUB178):**

0. absent

1. present

**177.** **Craniocaudally elongate foramina ventral to transverse processes on cranial caudal centra (W Alamosaurus 1):**

0. absent

1. present

**178.** **Ratio of craniocaudal length (without articular condyle if present):dorsoventral height of cranial caudal centra (U132*, SLL18*, UBD177, H172, MUB26*):**

0. >=0.6

1. <0.6

**179.** **Spinoprezygapophyseal lamina on cranial caudal neural arches (U140*, CRF85, W121, UBD188*, CR217, H173, CARB205*):**

0. absent

1. present and extending onto lateral aspect of neural spine

**180.** **Spinoprezygapophyseal-spinopostzygapophyseal lamina contact on cranial caudal neural arches (U140*, CRF86, W122, UBD188*, CR218, H174*, GRPP65, CARB206, MUB198):**

0. absent

1. present, forming prominent lamina on lateral aspect of neural spine

**181.** **Prespinal lamina on cranial caudal neural arches (U140*, SZ24*, G87*, SLL24*, CRF87, W123, P23*, UBD188*, CR219, H175, GRPP63, CARB207):**

0. absent

1. present

**182.** **Postspinal lamina on cranial caudal neural arches (U140*, SZ24*, SLL24*, CRF88, W124, P23*, UBD188*, CR220, H176, GRPP64):**

0. absent

1. present

**183.** **Postspinal fossa on cranial caudal neural arches (CRF89, W125, UBD188*, CR221, H177):**

0. absent

1. present

**184.** **Hyposphenal ridge on cranial caudal neural arches (U139, UBD187, H178, CARB203, MUB187*):**

0. absent

1. present

**185.** **Transverse process morphology on cranial caudal vertebrae (CS32*, U143*, S11*, G84*, CRF91*, W128*, UBD190-192*, CR223*, H179, GRPP72*, CARB198*, MUB204*):**

0. simple, flattened processes

1. triangular or aliform process (connected via laminae to neural arch) on caudal 1 only

2. triangular or aliform processes through caudal 3

3. triangular or aliform processes on or beyond caudal 4

**186.** **Dorsoventral extent of proximal end of transverse processes on cranial caudal vertebrae (U142*, W127, H180, CARB200):**

0. shallow (on centrum only)

1. deep (extending from centrum to neural arch)

**187.** **Diapophyseal laminae (= cranial [anterior] and caudal [posterior] centrodiapophyseal, prezygodiapophyseal, and postzygodiapophyseal laminae) on cranial caudal transverse processes (CRF92, W129, CR224, H181, CARB201, MUB189*):**

0. absent

1. present

**188.** **Morphology of cranial (= anterior) centrodiapophyseal lamina on cranial caudal transverse processes (CRF93, W130, CR225, H182, CARB202):**

0. single

1. divided

**189.** **Ratio of transverse width:craniocaudal length of cranial caudal neural spines (U141, SZ19, SLL23, CRF90*, CRF218, W126*, P18*, UBD189, CR215*, CR222*, H183, CPG42*, GRPP60*, GA53*, CARB199*, MUB33*, GROD56*, SGP52*):**

0. <=1.0 (subequal or longer than wide)

1. >1.0 (wider than long)

**190.** **Lateral surfaces of cranial and middle caudal centra (CRF94, W131, CR229*, H186*, CARB208*):**

0. rounded (centrum cylindrical or subcylindrical)

1. flat (centrum quadrangular)

**191.** **Ventral longitudinal sulcus on cranial and middle caudal centra (U136*, SLL22*, CRF95, CRF215*, W132, UBD182-183*, CR214*, CR230*, H187, CARB195*, CARB209, D54, MUB181*):**

0. absent

1. present

**192.** **Ratio of length:height of middle caudal centra (UBD179, H184, CARB215, MUB29*):**

0. <2.0

1. >=2.0

**193.** **Sharp ridge on lateral surface of middle caudal vertebrae at neural arch-centrum junction (SCC35, SLL27, GR19, UBD186, H185):**

0. absent

1. present

**194.** **Morphology of articular surfaces in middle caudal centra (U137*, CRF216*, GR15*, UBD184, H186*, CPG35*, GRPP55*, GA46*, MUB28*, GROD49*, SGP45*):**

0. subcircular

1. dorsoventrally compressed

2. transversely compressed

**195.** **Position of neural arches over centra on middle caudal vertebrae (CS35, SCC15, U138, G86, SLL28, CRF217, GR18*, UBD185, CR235, H189, CARB211, D58*, MUB192):**

0. roughly equal amounts on either side of centrum midpoint

1. over cranial half of centrum

**196.** **Orientation of middle caudal neural spines (CRF96, W133, CR232*, H190, CARB213*, D60*):**

0. distodorsal

1. vertical

**197.** **Morphology of cranial articular face of middle and distal caudal centra (SCC23*, U131, G82-83*, SLL25*, CRF98, CRF214*, W134, GR17*, UBD175*, CR231*, CR237*, H188, CPG38*, GRPP53-54*, GA49*, CARB210*, CARB217*, D61, MUB184*, GROD52-53*, SGP48*):**

0. amphicoelous/amphiplatyan

1. procoelous (conical)

**198.** **Ratio of transverse width:dorsoventral height of distal caudal centra (SCC34*, G81*, CRF99, W135, GR15*, CR236, H191*, CARB218, D62, MUB30*):**

0. <2.0 (centra cylindrical or subcylindrical)

1. >=2.0 (centra strongly compressed dorsoventrally)

**199.** **Number of biconvex distal caudal centra (WS108*, G83*, CRF100*, CRF102*, W136*, W138*, UBD176*, CR237*, CR239*, H192-193*, CARB220-221*, MUB186*):**

0. 0

1. 1-10

2. >=30

**200.** **Ratio of distalmost caudal centra length:width (CS36*, U134*, S8*, CRF101*, W137*, UBD180*, CR238, H194, CARB219*, CARB222*, MUB31*):**

0. <=4.0

1. 4.01-4.99

2. >=5.0

**201.** **Angle between tuberculum and capitulum of cervical ribs (WS71, G45, W139, UBD121, H195, CARB178):**

0. greater than 90°

1. less than 90° (rib ventrolateral to centrum)

**202.** **Length of cervical rib shafts (U94, G39*, CRF103, W140, UBD120*, CR240, H196, CARB177, MUB139*):**

0. much longer than centrum (overlapping as many as three subsequent vertebrae)

1. shorter than centrum (little or no overlap)

**203.** **Pneumatization of dorsal ribs (WS97*, G74*, CRF104*, W141*, UBD160, CR241, H197, CARB179*, D63*, MUB170):**

0. absent

1. present

**204.** **Morphology of proximal ends of cranial dorsal ribs (U119, CRF212, UBD159, H198):**

0. shallowly concave on both cranial and caudal faces

1. strongly convex cranially and deeply concave caudally

**205.** **Cross-sectional shape of cranial dorsal ribs (CRF105, W142, CR242, H199, CARB180, D64, MUB171):**

0. subcircular

1. 'plank-like' (craniocaudal dimension > three times mediolateral dimension)

**206.** **Hemal arch persistence (CRF109*, W147, CR246*, H200, CARB227):**

0. throughout at least 80% of tail

1. disappearing by caudal 30

**207.** **Morphology of hemal arches on middle and distal caudal vertebrae (CS37*, U147-148*, WS41*, S17*, G89*, CRF106*, CRF 219*, W143-144*, UBD197-198*, CR243*, CR248*, H201, GRPP73*, CARB223-224*, MUB210-211*):**

0. simple or curve caudoventrally (forming caudal process)

1. develop small cranial process

2. cranial and caudal processes elongate so arch is proximodistally much longer than tall dorsoventrally ('skid-like')

**208.** **Ratio of hemal canal dorsoventral height:total hemal arch length (CRF108*, W146*, UBD196, CR245*, H202, CARB226*, D67*, MUB35*):**

0. <0.30

1. >=0.30

**209.** **'Crus' bridging proximal margin of hemal canal (C34*, SCC11*, U145-146*, WS87*, S18*, G88*, CRF107*, W145*, GR24*, UBD194-195*, CR244*, H203, CARB225*, D65*, MUB208*):**

0. present in cranial through distal hemal arches

1. present in cranial hemal arches but absent in middle and distal hemal arches

2. absent in cranial through distal hemal arches

**210.** **Distal ends of distal hemal arches (U149*, CRF110, CRF220*, W148, UBD199*, CR247, H204, CARB228):**

0. fused

1. unfused (open)

**211.** **Ratio of forelimb:hind limb length (U158-159*, WS1*, W149*, UBD214-215*, H205, CARB229*):**

0. <=0.6

1. 0.6-0.74

2. >=0.75

**212.** **Ratio of humerus:femur proximodistal length (CS48*, S12*, CRF130*, W172*, UBD216*, CR290*, H206, CPG62*, GRPP93*, GA73*, CARB252*, D81*, MUB40*, GROD77*, SGP72*):**

0. <0.60

1. 0.60-0.89

2. >=0.90

**213.** **Size of scapular acromion (U150*, WS72, G90, W150, UBD200*, H208, CARB230, MUB36*):**

0. small and narrow

1. broad (dorsoventral width more than 150% minimum width of scapular blade)

**214.** **Deltoid crest (= crest of acromial process) (U151, UBD201, H209):**

0. absent

1. present

**215.** **Morphology of portion of acromion caudal to deltoid crest (UBD202, H210, MUB212):**

0. flat or convex and decreases in mediolateral thickness towards caudal margin

1. forms distinct fossa

**216.** **Orientation of scapular blade with respect to coracoid articulation (CRF111, W151, CR250, H211, CARB231):**

0. roughly perpendicular

1. roughly 45° angle

**217.** **Morphology of scapular blade (CS38*, WS109*, S19*, G92*, CRF112, W152, UBD206*, CR251-252*, H212, CARB232, D71*, MUB37*):**

0. acromial (dorsal) edge not expanded (parallels long axis of blade)

1. acromial edge with rounded dorsal expansion

2. racquet-shaped (dorsoventrally expanded)

**218.** **Orientation of scapular glenoid fossa (WS104, CRF113, W153, GR25, UBD203, CR256, H213, CPG48, GRPP74, GA59, CARB240, D68, MUB213, GROD63, SGP58):**

0. flat or facing laterally

1. strongly bevelled medially

**219.** **Cross-sectional shape of proximal end of scapular blade (CRF114, W154, CR257, H214, CARB241, D70*, MUB217):**

0. flat or rectangular

1. D-shaped

**220.** **Dorsal ridge or eminence on medial surface of scapular blade (SCC33, SZ31, G91, SLL33, P30, UBD204, H215):**

0. absent

1. present

**221.** **Ventral ridge on medial surface of scapular blade (SZ32, P31, UBD205, H216):**

0. absent

1. present

**222.** **Ratio of craniocaudal length of coracoid:maximum length of scapula-coracoid articulation (CRF115*, W155*, CR258*, H217*, CARB242*, D73*, MUB38*):**

0. <1.0 (articular surface longer)

1. >=1.0 (craniocaudal length longer)

**223.** **Morphology of craniodorsal margin of coracoid (SCC29*, U153, SZ33*, G95*, SLL35*, CRF116*, W156, GR30*, P32*, UBD208, CR259, H218, CPG54*, GRPP85*, GA65*, CARB243, MUB218, D74, GROD69*, SGP64*):**

0. rounded (cranial and dorsal margins grade into one another)

1. rectangular (meet at abrupt angle)

**224.** **Position of dorsal margin of coracoid with respect to dorsal margin of scapula (U152, SLL34*, UBD207, H219, CARB244, D72*, MUB219*):**

0. equal or dorsal to acromion

1. ventral to acromion and separated from it by V-shaped notch

**225.** **Infraglenoid lip of coracoid (CRF117, W157, CR261, H220, CARB246, MUB220):**

0. absent

1. present

**226.** **Morphology of sternal plate (CS39*, U155*, SZ29*, G93*, SLL30*, CRF118*, W158*, GR28*, P28*, UBD210, CR263*, H221, CPG52*, GRPP83*, GA63*, CARB247*, D75*, MUB221, GROD67*, SGP62*):**

0. ovoid

1. triangular due to presence of acute craniolateral projection

2. elliptical with concave lateral margin

**227.** **Prominent parasagittally oriented ridge on the dorsal surface of the sternal plate (U157, UBD212, CARB249):**

0. absent

1. present

**228.** **Ridge on cranioventral surface of sternal plate (SZ30*, P29*, UBD213, H223, CARB250):**

0. absent

1. present

**229.** **Prominent caudolateral expansion of sternal plate producing reniform profile in dorsal view (SCC26*, U156*, SLL31*, UBD211, H224, CARB248, MUB222):**

0. absent

1. present

**230.** **Ratio of maximum length of sternal plate:length of humerus (U154*, G94*, SLL32*, CRF221*, UBD209, CR264*, H222, CARB251, D77*, MUB39*):**

0. <0.75

1. >=0.75

**231.** **Proximal and lateral margins of humerus (CRF119, W159, GR27*, UBD218, CR271, H225*, CPG50*, GRPP76*, GA61*, CARB260, MUB223, GROD65*, SGP60*):**

0. merge smoothly with each other (humeral proximolateral corner rounded)

1. meet at an abrupt angle (humeral proximolateral corner sharp or squared)

**232.** **Supracoracoideus tuberosity on proximolateral portion of humerus (U160, SLL37*, GR27*, UBD217, H226, CPG50*, GRPP76*, GA61*, GROD65*, SGP60*):**

0. absent

1. present

**233.** **Development of humeral deltopectoral crest (WS3, SZ35*, G96, CRF120*, W160, P34*, UBD219, CR268*, H227, GRPP77*, CARB253):**

0. prominent

1. reduced to low crest or ridge

**234.** **Position of deltopectoral crest (SZ35*, P34*, UBD220, H228, MUB225):**

0. restricted to lateral edge of humerus

1. expanded medially across cranial face of humerus

**235.** **Morphology of humeral deltopectoral crest (CRF121*, SLL38, W161, CR269, H229, CARB254, D82):**

0. relatively narrow throughout length

1. markedly expanded distally

**236.** **Cross-sectional shape of humerus at mid-shaft (W162, H230, CARB255, MUB43*):**

0. circular

1. elliptical with long axis oriented mediolaterally

**237.** **Extent of distal articular surface of humerus (CRF122*, W163*, UBD222, CR273*, H231, CARB257*, MUB229):**

0. restricted to distal end (articular surface flat)

1. exposed on cranial and caudal portions of humeral shaft (forming convex articular surface)

**238.** **Caudodistal surface of humerus (SZ36, SLL39, P35, UBD221, H232, MUB228):**

0. shallowly concave

1. deeply concave, bounded by prominent vertical ridges

**239.** **Morphology of distal humeral articular surface (CRF123*, W164, CR274*, H233, CARB258, D84*, MUB230*):**

0. divided

1. flat (separate condyles indistinct)

**240.** **Morphology of proximal ulna (WS5, G98, W165, UBD223, H234, CARB261):**

0. subtriangular

1. triradiate with deep radial fossa

**241.** **Morphology of proximal articular surface of craniomedial process of ulna (U161, SLL41, CRF222, UBD224, CR277, H235, GRPP80, MUB234):**

0. flat

1. strongly concave

**242.** **Relative length of proximal ulnar condylar processes (CRF124, W166, CR278, H236, CARB262, D85*, MUB51*):**

0. subequal

1. unequal (with craniolateral process longer)

**243.** **Development of ulnar olecranon process (WS4, G97, CRF125*, W167, CR279, H237, CPG51, GRPP79*, GA62, CARB263, MUB233*, GROD66*, SGP61):**

0. prominent (projecting beyond proximal articular surface)

1. rudimentary (level with proximal articular surface)

**244.** **Ratio of proximodistal length:proximal breadth of ulna (CRF126, W168, CR276*, H238, CARB264, MUB50*):**

0. gracile

1. stout

**245.** **Ratio of maximum diameter of proximal end of radius:radius length (U162*, G99*, SLL40*, CRF223*, UBD225, CR285*, H239, GRPP82*, MUB45):**

0. <0.30

1. >=0.30

**246.** **Morphology of distal condyle of radius (WS6, G100, CRF127*, W169, UBD226, CR286-287*, H240, CARB265, MUB48*):**

0. round

1. subrectangular (flattened caudally and articulating on cranial side of ulna)

**247.** **Ratio of distal:midshaft mediolateral breadth of radius (CRF128*, W170*, CR288*, H241, GRPP82*, CARB266*, MUB47*):**

0. <1.50

1. 1.50-1.90

2. >1.90

**248.** **Orientation of distal radial condyle with respect to long axis of shaft (CRF129, W171, CR289, H242, CARB267, D87, MUB49*):**

0. perpendicular

1. bevelled approximately 20° proximolaterally

**249.** **Number of ossified carpal bones (U163-165*, WS79*, SLL42*, CRF131*, W173*, UBD228-230*, H243, CARB268*, D88*, MUB237-238*):**

0. >=3

1. 2

2. 1

3. 0

**250.** **Morphology of carpal bones (WS42, W174, UBD227, H244, CARB269):**

0. round

1. blocky (with flattened proximal and distal articular surfaces)

**251.** **Morphology of metacarpus (U169, WS80, G101, CRF132, W175, UBD235, H245, CARB270, D95):**

0. spreading

1. bound (metacarpals with subparallel shafts and articular surfaces extending half their length)

**252.** **Morphology of proximal surface of metacarpals in articulation (WS81, G102, CRF133, W176, H246, CARB271):**

0. gently curving to form 90° arc

1. U-shaped (subtending arc of 270°)

**253.** **Triangular, striated areas for ligament attachment on proximal parts of metacarpal shafts (UBD236, H247):**

0. absent

1. present

**254.** **Ratio of length of longest metacarpal:length of radius (CS49*, SCC5*, SCC12*, U167*, WS93*, G103*, CRF134*, W177*, GR31*, UBD233*, CR291*, H248, CARB272*, D89*, MUB52*):**

0. <0.35

1. 0.35-0.45

2. >0.45

**255.** **Ratio of length of metacarpal I:length of metacarpal II or III (whichever is longer) (U166, SLL43*, CRF224*, UBD232, CR292*, H249, MUB54*):**

0. <=1.0

1. >1.0 (metacarpal I is longest metacarpal)

**256.** **Ratio of length of metacarpal I:length of metacarpal IV (WS94, G105, CRF135, W178, UBD231, CR293, H250, CARB273, D90, MUB55):**

0. <1.0

1. >=1.0

**257.** **Morphology of distal condyle of metacarpal I (WS98, G104, CRF136, W179, CR296, H251, CARB274, D91):**

0. divided

1. undivided

**258.** **Orientation of mediolateral axis of metacarpal I distal condyle with respect to axis of shaft (CRF137, W180, CR297, H253, CARB275, D92, MUB240*):**

0. bevelled ~20° proximodistally

1. perpendicular

**259.** **Ratio of length of metacarpal V:length of longest metacarpal (U168, UBD234, H252):**

0. <0.90

1. >=0.90

**260.** **Manual phalangeal formula (SCC27*, U170-171*, WS43*, G130*, SLL44*, CRF138, W181, GR32*, UBD238-239*, UBD241*, CR308, H254, CPG55*, GRPP86*, GA66*, CARB276, D96*, MUB242*, GROD71*, SGP65*):**

0. 2-3-4-3-2 or more

1. reduced to 2-2-2-2-2 or fewer

2. completely or almost completely absent or unossified (distal ends of metacarpals lack articular surfaces)

**261.** **Morphology of manual phalanx I-1 (CRF139, W182, CR309, H255, CARB277, MUB243):**

0. rectangular

1. wedge-shaped

**262.** **Ratio of proximodistal length:mediolateral width of manual nonungual phalanges (WS44, CRF140*, W183, UBD237, CR310*, H256, CARB278):**

0. >1.0 (longer than wide)

1. <1.0 (wider than long)

**263.** **Size of ungual on manual digit I (SCC16*, UBD240, H257, MUB56*):**

0. large (at least 50% length of metacarpal I)

1. reduced (<25% length of metacarpal I) or absent

**264.** **Cranial transverse dimension of pelvis (WS82, CRF141, W184, CR311, H258, CARB279):**

0. narrow (ilia longer craniocaudally than distance separating preacetabular processes)

1. wide (distance between preacetabular processes exceeds craniocaudal dimension of ilia)

**265.** **Morphology of dorsal margin of ilium body (in lateral view) (U175, G111, W186, UBD247, H259, CARB281):**

0. flat, sigmoid, or gently convex

1. semicircular (markedly convex)

**266.** **Position of dorsalmost point on ilium (G110*, UBD245, CR313*, H260, CARB284, MUB248):**

0. caudal or dorsal to base of pubic peduncle

1. cranial to base of pubic peduncle

**267.** **In lateral view, the cranioventralmost point on the iliac preacetabular process (CS40*, SCC17*, U173, WS99, G106, G108*, SLL45*, CRF144, CRF225, W188, UBD244, CR312*, CR318*, H261, GA70*, CARB282, D97*, MUB246, SGP69*):**

0. is also the cranialmost point (preacetabular process is pointed)

1. is caudal to the cranialmost part of process (process is semicircular with caudoventral excursion of cartilage cap)

**268.** **Orientation of preacetabular process of ilium with respect to axis of body (SCC28*, U172*, SZ38*, G107*, SLL46*, CRF143*, W187*, GR38*, P37*, UBD242-243*, CR317*, H262, CPG60*, GRPP91*, GA71*, CARB283*, D98*, MUB244-245*, GROD75*, SGP70*):**

0. cranial in vertical plane

1. craniolateral in vertical plane

2. craniolateral and cranial edge curls laterally into horizontal plane

**269.** **Projected line (chord) connecting articular surfaces of pubic and ischial peduncles of ilium (U176*, UBD249, H264):**

0. passes ventral to ventral margin of postacetabular portion of ilium

1. passes through or dorsal to ventral edge of postacetabular portion of ilium

**270.** **Size of ischial peduncle of ilium (SCC3, U176, WS7, G112, CRF142*, W185, UBD248, CR316*, H263, CARB280):**

0. large and prominent (long axis of ilium roughly horizontal)

1. low and rounded (long axis of ilium oriented craniodorsally-caudoventrally)

**271.** **Brevis fossa on postacetabular process of ilium (U174, UBD246, H265):**

0. present

1. absent

**272.** **Development of ambiens process of pubis (CS42, U177, S20, CRF145, W189, UBD250, CR319, H266, CARB286):**

0. absent or small, striated area confluent with cranial margin of pubis

1. prominent and projecting cranial to cranial margin of pubis ('hook-like')

**273.** **Morphology of pubic 'apron' (U179, WS46*, W190, UBD252, H267, CARB287):**

0. flat (with straight symphysis, proximal end in parasagittal plane but middle and distal ends in mediolateral plane)

1. canted craniomedially (middle and distal ends in same plane as proximal end; gently sigmoid symphysis and V-shaped in cross section at body midlength)

**274.** **Ratio of length of puboischiadic contact:proximodistal length of pubis (CS41*, SCC13*, U180*, WS95*, G116*, SLL49*, CRF146*, W191*, UBD253, CR323*, H268, CARB288*, D101*, MUB59*):**

0. <0.40

1. >=0.40

**275.** **Ratio of proximodistal length of ischium:length of pubis (CS45*, SCC24*, U178, WS8*, G113*, SLL48*, CRF147*, CRF226*, W192*, GR33*, UBD251, CR324-325*, H269, CPG56*, GRPP87*, GA67*, CARB285*, D102*, MUB60*, GROD70*, SGP66*):**

0. <0.90

1. >=0.90

**276.** **Tuberosity on lateral surface of iliac peduncle of ischium (UBD255, H270):**

0. absent

1. present

**277.** **Projected line (chord) of long axis of ischium in articulation with ilium (U185*, UBD259, H271, MUB254):**

0. passes through ventral part of acetabulum or dorsal part of pubic articular surface (long axis of ischium ~60º to horizontal)

1. passes through dorsal part of acetabular margin or approaches rim of iliac articulation (long axis of ischium ~80º to horizontal)

**278.** **Morphology of ischium shaft distal to pubic peduncle (CS46*, SLL51*, CRF148, W193, UBD254*, CR326, H272, CARB292, D103, MUB253):**

0. emarginate (sagittal notch present between articulated ischia)

1. not emarginate

**279.** **Morphology of distal shaft of ischium (WS9, G114, CRF149, W194, CR327, H273, CARB295):**

0. craniocaudal depth increases medially but not laterally

1. blade-like (craniocaudal depths on both medial and lateral sides subequal)

**280.** **Ratio of mediolateral width of distal end of ischium:proximodistal length of ischium (U182*, SLL50*, CRF227*, UBD256, H274, MUB63*):**

0. <=0.15

1. >0.15

**281.** **Ratio of mediolateral width:craniocaudal thickness of distal end of ischium body (UBD260*, H275):**

0. <2.0

1. >=2.0

**282.** **Cross-sectional morphology of distal ischial shafts (CS43, U184*, WS88, S22*, G115, CRF150, W195, UBD258*, CR328, H276, CARB296):**

0. V-shaped (forming marked angle to one another)

1. flat (nearly coplanar)

**283.** **Expansion of distal end of ischium shaft (CS44*, U183, S21*, UBD257, H277, CARB297, MUB64*):**

0. slight

1. strong dorsoventrally

**284.** **Morphology of cranial face of femoral shaft in lateral view (SCC4*, U186, UBD266, H278):**

0. convex

1. straight

**285.** **Orientation of femoral head in cranial view (UBD263, H279, CARB304*):**

0. medial or ventromedial

1. dorsomedial

**286.** **Position of fourth trochanter on femoral shaft (U189, UBD268, H280, CARB308):**

0. on caudal surface, near midline

1. on caudomedial margin

**287.** **Development of fourth trochanter of femur (U190, WS11, SZ41, G117, SLL53, W196, P40, UBD269, H281, CARB299*, D109*):**

0. prominent and blade-like

1. reduced to low crest or ridge

**288.** **Position of distal margin of fourth trochanter (UBD267, H282):**

0. lies proximal to midshaft

1. lies at or distal to midshaft

**289.** **Morphology of lesser trochanter of femur (SCC6*, U188*, W197*, UBD261-262*, H283, CARB300*):**

0. well-developed ridge or plate

1. weakly developed ridge or plate

2. absent

**290.** **Ratio of mediolateral:craniocaudal diameter of femur at midshaft (U191*, WS10*, CRF151*, W198*, UBD270*, CR336*, H284*, CARB301*, MUB65*):**

0. <1.25 (~1.0)

1. 1.25-1.50

2. >1.50 (usually approximately 1.85 or greater)

**291.** **Morphology of lateral margin of femoral shaft (CS47*, SCC19*, WS100, SZ40*, G118, SLL52*, CRF152, W199, P39*, UBD265, CR335, H285, D107, MUB255*):**

0. straight

1. proximal 1/3 deflected medially

**292.** **Morphology of middle and distal portion of femoral shaft in cranial view (UBD264, H286):**

0. sigmoid

1. straight

**293.** **Relative mediolateral breadth of distal femoral condyles (CRF153*, W200*, UBD271*, CR337*, H287*, CARB305*, M66*):**

0. subequal

1. tibial condyle much broader than fibular condyle

2. fibular condyle much broader than tibial condyle

**294.** **Orientation of femoral distal condyles with respect to femoral shaft (CRF154, W201, CR338, H288, CARB306, D110, MUB259*):**

0. perpendicular or slightly bevelled dorsolaterally

1. bevelled dorsomedially ~10°

**295.** **Morphology of articular surface of femoral distal condyles (CRF155*, W202*, UBD272, CR339*, H289, CARB307*, SGP74*):**

0. restricted to distal portion of femur

1. expanded onto cranial and caudal portion of femoral shaft (surfaces visible in cranial and caudal views)

**296.** **Ratio of tibia:femur length (U192*, UBD273, H290):**

0. >=0.70

1. <0.70

**297.** **Morphology of tibial proximal condyle (WS83*, SZ42*, CRF156*, W203*, P41*, UBD274, CR340*, H291, CARB309*):**

0. longer craniocaudally than mediolaterally by at least 15%

1. expanded mediolaterally (craniocaudal and mediolateral dimensions <15% each other)

**298.** **Tibial cnemial crest (UBD275, H292):**

0. prominent

1. reduced to low ridge

**299.** **Orientation of tibial cnemial crest (WS47, G119, W204, UBD276, CR341*, H293*, CARB310, MUB260*):**

0. projects cranially or craniolaterally

1. projects laterally

**300.** **Ratio of distal mediolateral dimension of tibia:midshaft mediolateral dimension (CRF157*, W205*, CR344*, H294, CARB311*, MUB67*):**

0. <2.0

1. >=2.0

**301.** **Size of distal caudoventral process of tibia (WS48, G120, W206, UBD278, H295, CARB312):**

0. broad mediolaterally (covering caudal fossa of astragalus)

1. shortened mediolaterally (caudal fossa of astragalus visible caudally)

**302.** **Distal end of tibia (SCC7, SZ42*, SLL54*, P41*, UBD277, CR343, H296, CPG64*, GRPP96*, GA75*, MUB68*, GROD79*, SGP75*):**

0. wider mediolaterally than craniocaudally

1. roughly equal mediolateral and craniocaudal dimensions

**303.** **Development of proximal tibial scar on fibula (WS62, W207, UBD279, H297, CARB313):**

0. not well marked

1. well marked and deepening cranially

**304.** **Morphology of fibular lateral trochanter (U193*, WS49*, G121-122*, SLL55*, CRF158*, CRF228*, W208*, UBD280-281*, CR347*, H298, CARB314*; MUB263*):**

0. absent

1. present and ovoid

2. present, comprised by two vertically elongate, parallel ridges

**305.** **Size of distal condyle of fibula (CRF159, W209, CR349*, H299, CARB315, D114*, MUB69*):**

0. subequal to shaft

1. expanded mediolaterally (> twice midshaft mediolateral dimension)

**306.** **Morphology of astragalus (WS85, CRF160, W210, UBD284, H300, CARB316, MUB265):**

0. rectangular

1. wedge-shaped (with reduced craniomedial corner)

**307.** **Craniocaudal dimension of astragalus as seen in dorsal view (U195, UBD285, H301):**

0. widens medially

1. narrows medially

**308.** **Morphology of ventral surface of astragalus (U194, UBD282, H302):**

0. flat or slightly concave mediolaterally

1. convex mediolaterally

**309.** **Vascular foramina at base of ascending process of astragalus (WS12, W211, UBD286, H303, CARB318):**

0. present

1. absent

**310.** **Extent of ascending process of astragalus (WS84*, G124*, CRF161*, W212*, UBD283, H304, CARB319, MUB266):**

0. terminates cranial to caudal edge

1. extending to caudal margin

**311.** **Morphology of caudal fossa of astragalus (WS63, CRF162*, W213, UBD287, CR350*, H305, CARB320):**

0. undivided

1. divided by vertical, caudomedially-oriented crest

**312.** **Ratio of mediolateral width:maximum craniocaudal length of astragalus (CRF163*, W214*, H306, CARB321*, D115*, MUB71*):**

0. <=1.25 (dimensions subequal)

1. >1.25

**313.** **Ossified calcaneum (U196, G123, CRF164, W215, UBD288, CR352, H307, GRPP98, CARB322, MUB270, GROD81):**

0. present

1. absent

**314.** **Ossified distal tarsals 3 and 4 (U197, WS13, W216, UBD289*, H308, CARB323):**

0. present

1. absent

**315.** **Posture of metatarsus (WS52, G125, W217, UBD291*, H309, CARB324):**

0. bound

1. spreading

**316.** **Angle between long axis of shaft of metatarsal I and plane of proximal articular surface as seen in cranial view (W218, H310, CARB325):**

0. perpendicular

1. angled ventromedially

**317.** **Angle between long axis of shaft of metatarsal I and plane of distal articular surface as seen in cranial view (CRF165*, W219, CR355*, H311, CARB326):**

0. perpendicular

1. angled dorsomedially

**318.** **Caudolateral projection of distal condyle of metatarsal I (U199, S23, G127, CRF166*, W220, UBD293, CR356*, H312, CARB327, MUB272):**

0. absent

1. present

**319.** **Size of metatarsal I (U198, G126*, W221*, UBD292, H313, CARB328*):**

0. slender, reduced, or absent

1. robust (ratio of length:mediolateral width of proximal end <=1.5)

**320.** **Rugosities on distal parts of dorsolateral portions of bodies of metatarsals I-III (UBD294, H314):**

0. absent

1. present

**321.** **Size of proximal condyles of metatarsals I and V compared to metatarsals II, III, and IV (WS14*, CRF167*, W222*, UBD296*, CR357*, H315, CARB329*):**

0. smaller than

1. subequal to

**322.** **Ratio of length of metatarsal III:length of tibia (SCC5*, WS50*, G128*, W223*, UBD290*, UBD295*, H316, CARB330*, MUB73*):**

0. >=0.40

1. 0.26-0.39

2. <=0.25

**323.** **Ratio of minimum mediolateral shaft diameters of metatarsals III and IV:minimum mediolateral shaft diameters of metatarsals I or II (WS51*, WS73*, CRF168*, W224*, UBD297, CR359*, H317, CARB331*):**

0. >=0.65

1. <0.65

**324.** **Ratio of length of metatarsal V:length of metatarsal IV (WS15, W225, UBD298*, H318, CARB332):**

0. <0.70 (usually <<0.70)

1. >=0.70

**325.** **Relationship of plantar to proximal surface of pedal phalanx I-1 (UBD301, H319):**

0. meet at ~90° angle

1. meet at acute angle (area drawn into thin plate that projects caudal to distal condyles of metatarsal I)

**326.** **Collateral ligament fossae on non-ungual pedal phalanges (U202, G129, UBD302, H320):**

0. present

1. absent

**327.** **Ratio of maximum proximodistal:mediolateral dimensions of pedal nonungual phalanges (WS53, W226, UBD304, H321, CARB333):**

0. >1.0 (longer than wide)

1. <=1.0 (wider than long)

**328.** **Development of penultimate phalanges of pedal digits II-IV (WS55, W227, UBD305, H322, CARB334):**

0. subequal in size to more proximal phalanges

1. rudimentary or absent

**329.** **Morphology of pedal phalanx II-2 (U203, UBD303, H323):**

0. square or rectangular in dorsal view

1. reduced craniocaudally, irregular in shape, and semicircular in dorsal view

**330.** **Number of phalanges on pedal digit IV (U200-201*, UBD299-300*, H324, MUB277*):**

0. >=4

1. 3

2. <=2

**331.** **Orientation of pedal unguals with respect to digit axis (WS64, W228, UBD306, H325, CARB335):**

0. aligned

1. deflected ventrolaterally

**332.** **Length of pedal ungual I relative to pedal ungual II (WS16, CRF169*, W229, CR362*, H326, CARB336):**

0. subequal

1. 25% larger than

**333.** **Ratio of length of pedal ungual I:length of metatarsal I (WS54, W230, UBD307*, H327, CARB337):**

0. <1.0

1. >=1.0

**334.** **Ratio of mediolateral width:dorsoventral height of pedal ungual I (WS17, W231, H328, CARB338):**

0. >1.0 (wider than tall)

1. <=1.0 (sickle-shaped, much taller than wide)

**335.** **Morphology of pedal unguals II-III (WS56, W232, UBD308, H329, CARB339):**

0. broader mediolaterally than dorsoventrally

1. sickle-shaped (much deeper dorsoventrally than broad mediolaterally)

**336.** **Development of pedal ungual IV (WS57, W233, H330, CARB340):**

0. subequal in size to unguals of pedal digits II and III

1. rudimentary or absent

**337.** **Osteoderms (SZ43, G75, SLL56, CRF170, W234, P42, CR363, H331, CPG65, GRPP101, GA77, D119, MUB279, GROD82*):**

0. absent

1. present

**References for Appendix S1**

1. Carballido JL, Salgado L, Pol D, Canudo JI and Garrido A (2012) A new basal rebbachisaurid (Sauropoda, Diplodocoidea) from the Early Cretaceous of the Neuquén Basin; evolution and biogeography of the group. Historical Biology 24: 631–654.
2. Carballido JL and Sander PM (2014) Postcranial axial skeleton of *Europasaurus holgeri* (Dinosauria, Sauropoda) from the Upper Jurassic of Germany: implications for sauropod ontogeny and phylogenetic relationships of basal Macronaria. Journal of Systematic Palaeontology 12: 335–387.
3. Calvo JO, Porfiri JD, González-Riga BJ and Kellner AWA (2007) A new Cretaceous terrestrial ecosystem from Gondwana with the description of a new sauropod dinosaur. Anais da Academia Brasileira de Ciências 79: 529–541.
4. Calvo JO, González-Riga BJ and Porfiri JD (2007) A new titanosaur sauropod from the Late Cretaceous of Neuquén, Patagonia, Argentina. Arquivos do Museu Nacional, Rio de Janeiro 65: 485–504.
5. Curry Rogers K (2005) Titanosauria: a phylogenetic overview. In: K. Curry Rogers and J. A. Wilson, editors. The Sauropods: Evolution and Paleobiology. Berkeley: University of California Press. pp. 50–103.
6. Curry Rogers K and Forster CA (2001) The last of the dinosaur titans: a new sauropod from Madagascar. Nature 412: 530–534.
7. Calvo JO and Salgado L (1995) *Rebbachisaurus tessonei* sp. nov. a new Sauropoda from the Albian–Cenomanian of Argentina; new evidence on the origin of the Diplodocidae. Gaia 11: 13–33.
8. D'Emic MD (2012) The early evolution of titanosauriform sauropod dinosaurs. Zoological Journal of the Linnean Society 166: 624–671.
9. Gomani EM (1999) Dinosaurs of the Cretaceous Sedimentary Rocks of Northern Malawi, Africa. Dallas: Southern Methodist University. pp. 257.
10. Gallina PA and Apesteguía S (2011) Cranial anatomy and phylogenetic position of the titanosaurian sauropod *Bonitasaura salgadoi*. Acta Palaeontologica Polonica 56: 45–60.
11. González Riga BJ (2003) A new titanosaur (Dinosauria, Sauropoda) from the Upper Cretaceous of Mendoza Province, Argentina. Ameghiniana 40: 155–172.
12. González Riga BJ and Ortiz David L (2014) A new titanosaur (Dinosauria, Sauropoda) from the Upper Cretaceous (Cerro Lisandro Formation) of Mendoza Province, Argentina. Ameghiniana 51: 3–25.
13. González Riga BJ, Previtera E and Pirrone CA (2009) *Malarguesaurus florenciae* gen. et sp. nov., a new titanosauriform (Dinosauria, Sauropoda) from the Upper Cretaceous of Mendoza, Argentina. Cretaceous Research 30: 135–148.
14. Harris JD (2006) The significance of *Suuwassea emilieae* (Dinosauria: Sauropoda) for flagellicaudatan intrarelationships and evolution. Journal of Systematic Palaeontology 4: 185–198.
15. Mannion PD, Upchurch P, Barnes RN and Mateus O (2013) Osteology of the Late Jurassic Portuguese sauropod dinosaur *Lusotitan atalaiensis* (Macronaria) and the evolutionary history of basal titanosauriforms. Zoological Journal of the Linnean Society 168: 98–206.
16. Powell JE (2003) Revision of South American titanosaurid dinosaurs: palaeobiological, palaeobiogeographical and phylogenetic aspects. Records of the Queen Victoria Museum 111: 1–173.
17. Salgado L (1999) The macroevolution of the Diplodocimorpha (Dinosauria; Sauropoda): a developmental model. Ameghiniana 36: 203–216.
18. Salgado L, Coria RA and Calvo JO (1997) Evolution of titanosaurid sauropods. I: phylogenetic analysis based on the postcranial evidence. Ameghiniana 34: 3–32.
19. Salgado L, Gallina PA and Paulina Carabajal A (2015) Redescription of *Bonatitan reigi* (Sauropoda: Titanosauria), from the Campanian–Maastrichtian of the Río Negro Province (Argentina). Historical Biology 27: 525–548.
20. Smith JB, Lamanna MC, Lacovara KJ, Dodson P, Smith JR, et al. (2001) A giant sauropod dinosaur from an Upper Cretaceous mangrove deposit in Egypt. Science 292: 1704–1706.
21. Sanz JL, Powell JE, Le Loeuff J, Martínez R and Pereda-Suberbiola X (1999) Sauropod remains from the Upper Cretaceous of Laño (northcentral Spain). Titanosaur phylogenetic relationships. In: H. Astibia, J. C. Corral, X. Murelaga, X. Orue-Etxebarria and X. Pereda-Suberbiola, editors. Geology and Palaeontology of the Upper Cretaceous Vertebrate-Bearing Beds of the Laño Quarry (Basque-Cantabrian Region, Iberian Peninsula). Alava: Museo de Ciencias Naturales de Alava. pp. 235–255.
22. Upchurch P (1998) The phylogenetic relationships of sauropod dinosaurs. Zoological Journal of the Linnean Society 124: 43–103.
23. Upchurch P, Barrett PM and Dodson P (2004) Sauropoda. In: D. B. Weishampel, P. Dodson and H. Osmólska, editors. The Dinosauria, Second Edition. Berkeley: University of California Press. pp. 259–322.
24. Wilson JA (2002) Sauropod dinosaur phylogeny: critique and cladistic analysis. Zoological Journal of the Linnean Society 136: 217–276.
25. Wilson JA and Sereno PC (1998) Early evolution and higher-level phylogeny of sauropod dinosaurs. Society of Vertebrate Paleontology Memoir 5: 1–68.
