# Supplementary figures and images for: A Basal Lithostrotian Titanosaur (Dinosauria: Sauropoda) with a Complete Skull: Implications for the Evolution and Paleobiology of Titanosauria
Source: PLoS One. 2016 Apr 26;11(4):e0151661. doi: 10.1371/journal.pone.0151661 (PMC4846048; doi:10.1371/journal.pone.0151661)

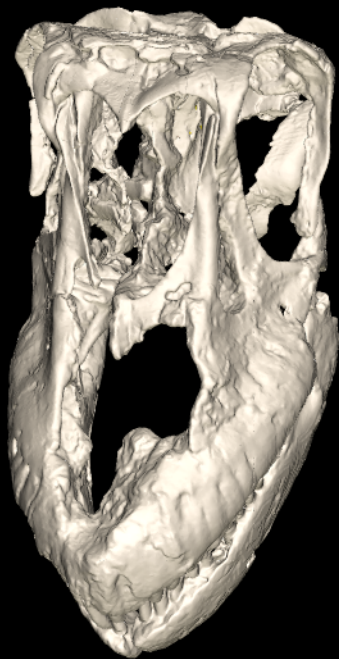

Supplement: S1 Fig — (Zaher et al. [14]) Color coding of reconstructed endocranial soft-tissues is as follows: endocast, lighter blue; endosseous inner ear labyrinth, pink; cranial nerves, yellow; arterial structures, red; venous structures, darker blue. (PDF) [file pone.0151661.s008.pdf]
